# The telomeric DNA damage response as a therapeutic target in idiopathic pulmonary fibrosis

**DOI:** 10.1038/s44321-026-00500-x

**Published:** 2026-08-12

**Authors:** Francesca Rossiello, Giada Cicio, Sara Sepe, Alexandra Mancheno-Ferris, Valeria Cancila, Arianna Castellini, Federica Rey, Giorgio Bertolazzi, Alessia Oppezzo, Eugenia Marinelli, Matteo Cabrini, Alessia di Lillo, Sara Tavella, Ilaria Rosso, Francesca M Buffa, Claudio Tripodo, Fabrizio d’Adda di Fagagna

**Affiliations:** 1 IFOM ETS - The AIRC Institute of Molecular Oncology, Milan, Italy; 2 Tumor Immunology Unit, Department of Health Sciences, University of Palermo, Palermo, Italy; 3 Bocconi Institute for Data Sciences and Analytics, Department of Computing Sciences, Bocconi University, Milan, Italy; 4 Department of Medicine and Surgery, University of Enna “Kore”, Enna, Italy; 5 Institute of Molecular Genetics IGM-CNR “Luigi Luca Cavalli-Sforza”, Pavia, Italy; 6 Present Address: Thermo Fisher Scientific – Patheon, mRNA Department, Monza, Italy; 7 Present Address: PSI CRO Italy S.r.l., Milan, Italy

**Keywords:** Respiratory System

## Abstract

Telomere dysfunction and the telomeric DNA damage response (tDDR) activation correlate with aging and age-related diseases, including idiopathic pulmonary fibrosis (IPF). However, a causal role for tDDR in IPF pathogenesis has not been determined. IPF patients frequently bear germline mutations in telomerase genes, critically short telomeres, and markers of tDDR and cellular senescence. We previously demonstrated that telomeric antisense-oligonucleotides (tASOs) targeting telomeric non-coding RNAs are selective tDDR inhibitors. Here, we employed late-generation telomerase knockout mice as a genetic model of IPF. Systemic tASOs treatment reduces DDR—including in stem/progenitor cells—inflammation, and lung fibrosis in young, adult, and old mice. Markers of DDR correlate with lung pathology, and tDDR inhibition normalizes molecular and pathological phenotypes, uncoupling telomere lengths from their deleterious consequences. Transcriptomic changes in telomerase knockout mice recapitulate those observed in normal aged mice and in IPF patients, and they are reversed upon tDDR inhibition. These results highlight the pathogenic causative relevance of tDDR activation in IPF pathogenesis and support tASOs as a promising therapeutic strategy for IPF and for telomere biology diseases.

## Synopsis

Idiopathic pulmonary fibrosis (IPF) is a fatal lung disease with no curative therapy. Telomere shortening contributes to IPF, but its pathogenic mechanisms remain unclear. Our study demonstrates that telomeric DNA damage response (tDDR) is a causal and targetable driver of lung fibrosis.

- Late generation telomerase knockout mice develop spontaneous lung fibrosis associated with elevated tDDR markers, collagen accumulation, inflammation, and emphysema‑like architectural defects.
- tDDR is selectively inhibited in lungs by telomeric antisense oligonucleotides (tASOs) targeting telomeric damage-induced non-coding RNAs (tdincRNAs) without changing telomere lengths.
- tDDR inhibition markedly reduces lung fibrosis, tissue damage, and profibrotic signaling pathways, also in setting of old age and advanced fibrosis.
- tDDR inhibition reverses age-related and telomere dysfunction-induced transcriptional programs, including those shared with IPF patients.

## Abstract (web summary)

Idiopathic pulmonary fibrosis (IPF) is a fatal lung disease with no curative therapy. Telomere shortening contributes to IPF, but its pathogenic mechanisms remain unclear. Our study demonstrates that telomeric DNA damage response (tDDR) is a causal and targetable driver of lung fibrosis.

## Graphical abstract

**Figure:**
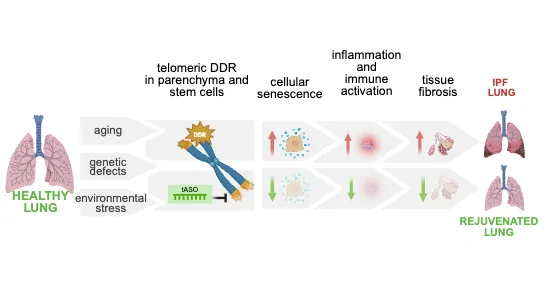

## Introduction

Aging is a complex biological process characterized by a gradual decline of cellular functions, including impaired genome integrity maintenance and accumulation of DNA damage over time (López-Otín et al, 2022). Among the various forms of genomic instability occurring with aging, DNA damage at telomeres is particularly consequential. Telomeres are the ends of linear chromosomes, made of TTAGGG repeats in vertebrates, whose length is maintained by telomerase, an RNA-dependent DNA polymerase composed of a protein (Tert) and an RNA (Terc) component. In the absence of sufficient expression of telomerase or of other telomere maintenance mechanisms, telomeres shorten at each cell division due to inherently incomplete DNA replication and nucleolytic activities (Harley et al, 1990). When critically short, telomere ends are sensed as damaged DNA and activate a DNA damage response (DDR) that permanently inhibits cell proliferation and enforces cellular senescence (d’Adda di Fagagna et al, 2003; Herbig et al, 2004). Due to the poor repairability of DNA breaks within telomeric repeats, as well as lesions within not-critically short telomeres, can cause a protracted DDR that promotes cellular senescence (Fumagalli et al, 2012; Hewitt et al, 2012). Overall, this points to the loss of telomere integrity as a crucial apical event in cellular senescence establishment mediated by telomeric DDR (tDDR) activation (d’Adda di Fagagna, 2008; Di Micco et al, 2020).

The DDR is a signaling cascade triggered by DNA discontinuities that employs post-translational protein modifications. The phosphorylation of the histone H2AX at serine 139, called γH2AX, by phosphatidylinositol-3-kinase-related kinases, such as ataxia telangiectasia mutated (ATM), promotes the recruitment on damaged chromatin of DDR factors, including p53-binding protein 1 (53BP1), the phosphorylated KRAB-associated protein 1 (pKAP1), and phosphorylated ATM (pATM) itself (Dantuma and van Attikum, 2016). We previously reported that DNA double-strand breaks (DSBs) in the genome trigger the transcription of both DNA strands (Francia et al, 2012; Michelini et al, 2017). Similarly, upon telomere dysfunction, telomeric damage-induced non-coding RNAs (tdincRNAs) are synthesized and are essential to recruit and activate DDR factors at damaged telomeres, thus fueling DNA damage signaling and repair (Rossiello et al, 2017). Sequence-specific telomeric antisense oligonucleotides (tASOs) that target tdincRNAs generated from either DNA strand selectively suppress tDDR and effectively uncouple telomere dysfunction from its downstream consequences, in cell culture and in vivo in mice (Rossiello et al, 2017). We previously employed this approach to demonstrate that tDDR inhibition by tASOs can ameliorate the progeroid pathological phenotypes and increase the survival of a skin-specific mouse model of Hutchinson-Gilford progeria syndrome (HGPS) characterized by telomere dysfunction and premature cellular senescence (Aguado et al, 2019), and that it can reduce telomere dysfunction-driven increased expression of ACE2, the SARS-CoV2 receptor, in mice (Sepe et al, 2022). tDDR inhibition by tASOs also reduces neurotoxicity in human and mouse neurons caused by Aβ42-induced tDDR activation, and restores transcriptional pathways dysregulated in Alzheimer’s disease patients (Sepe et al, 2025).

Telomeropathies, also known as telomere biology disorders (TBDs) or short telomere syndromes (STSs), are a group of pathologies caused by genetic mutations that lead to telomere dysfunction in the form of prematurely shortened and damaged telomeres (Armanios and Blackburn, 2012). Among them, idiopathic pulmonary fibrosis (IPF) is the most common manifestation, typically with a late onset *(*Armanios, 2012*)*. IPF is an interstitial lung disease characterized by progressive loss of alveolar epithelial cell integrity and stromal remodeling towards fibrotic changes, eventually causing a decline in respiratory function. IPF patients generally have a poor prognosis, with four years median survival after diagnosis. The only presently available therapies are anti-fibrotic treatments, which however are not curative and can only slow down disease progression, often at the cost of significant side effects (Hughes et al, 2016; Hujjat et al, 2026). While novel therapeutic options are currently being tested in clinical trials at different stages (Libra et al, 2024; Vernimmen, 2026), at present, a cure can only be provided by lung transplantation, with the inherent risks and costs. IPF affects ~3 million people worldwide, and its incidence is expected to increase because of the current aging population (Maher et al, 2021). Thus, there is a pressing need for safer and more efficacious approaches to treat an increasing number of IPF patients.

IPF incidence increases with age, and it is considered an age-related disease strongly associated with telomere dysfunction (Rossiello et al, 2022). Although the term idiopathic may suggest an unknown cause, 37% of familial cases and 25% of sporadic ones show leukocyte telomere lengths below the tenth percentile for their age (Cronkhite et al, 2008). One third of the familial cases show germline mutations in genes involved in telomere maintenance, particularly in the telomerase genes *TERT* and *TERC* (Alonso-Gonzalez et al, 2023; Borie et al, 2023), and patients bearing these mutations have a reduced transplant-free survival (Borie et al, 2016). Lung cells in IPF patients show short telomeres, and markers of tDDR activation and of cellular senescence, which increase with disease severity (Schafer et al, 2017; Lee et al, 2021). In independent cohorts, shorter telomere length is a robust independent predictor of disease progression, response to therapy, and death, and it is associated with a shorter time to allograft dysfunction following lung transplant (Stuart et al, 2014; Adegunsoye et al, 2023).

In a model of bleomycin-induced lung fibrosis, genetic or pharmacological clearance of senescent cells improves lung function and health (Schafer et al, 2017), providing additional evidence that cellular senescence is crucial for IPF pathogenesis. However, the acute and spontaneously reversible fibrosis that characterizes this model notoriously does not accurately recapitulate the progressive, chronic, and irreversible fibrosis observed in IPF patients (Moeller et al, 2008).

The development and characterization of transgenic mouse models demonstrated that telomere dysfunction recapitulates several human IPF features. Similar to IPF patients, mice with genetic inactivation of telomerase components and short telomeres show tDDR activation, inflammation, and fibrosis in the lungs (Chen et al, 2015; Piñeiro-Hermida et al, 2020). Furthermore, telomerase-deficient mice are more sensitive to bleomycin-induced fibrosis than wild-type mice (Povedano et al, 2015). Telomere elongation by forced re-expression of telomerase protein component TERT through adeno-associated vectors reduces tDDR, decreases inflammation and fibrosis, and improves lung function (Povedano et al, 2018; Piñeiro-Hermida et al, 2020). Importantly, telomere dysfunction induced by the genetic deletion of the telomere-binding proteins TRF1 or TRF2 specifically in lung alveolar epithelial type II cells, thought to have stem/progenitor functions, is sufficient to cause inflammation and fibrosis (Alder et al, 2015; Povedano et al, 2015), highlighting their crucial role in IPF pathogenesis.

Overall, the published evidence supports the notion that telomere shortening drives IPF in humans and that this can be modeled in animals with telomere dysfunction, such as telomerase-inactivated mouse models. A shared consequence of telomere dysfunction, independent from its initial genetic defect or cause, is the activation of tDDR pathways, which trigger cellular senescence, inflammation, and fibrosis. Therefore, tDDR appears to be an appealing nodal target to interfere with the downstream consequences of telomere dysfunction. However, it is unknown whether tDDR inhibition is sufficient to reduce the pathological consequences of telomere dysfunction, whether it can be successfully achieved in the lung, and whether it is safe. In this study, we investigated the impact of tDDR inhibition to assess its contribution to IPF pathology in a mouse model characterized by telomere dysfunction, and to test its therapeutic potential.

## Results

### Late generation *Terc*^*−/−*^ mice show increased DDR activation and fibrosis in the lung

Mutations in telomerase genes are the most common in IPF patients, occurring in 8–15% of familial and 1–3% of sporadic cases (Armanios, 2012). Mice lacking telomerase function undergo progressive telomere shortening in successive generations until they become short to the point of recapitulating features observed in IPF patients, including telomeric DDR (tDDR), cellular senescence, inflammation, and lung fibrosis (Chen et al, 2015; Liu et al, 2018; Piñeiro-Hermida et al, 2020).

**Figure EV1:**
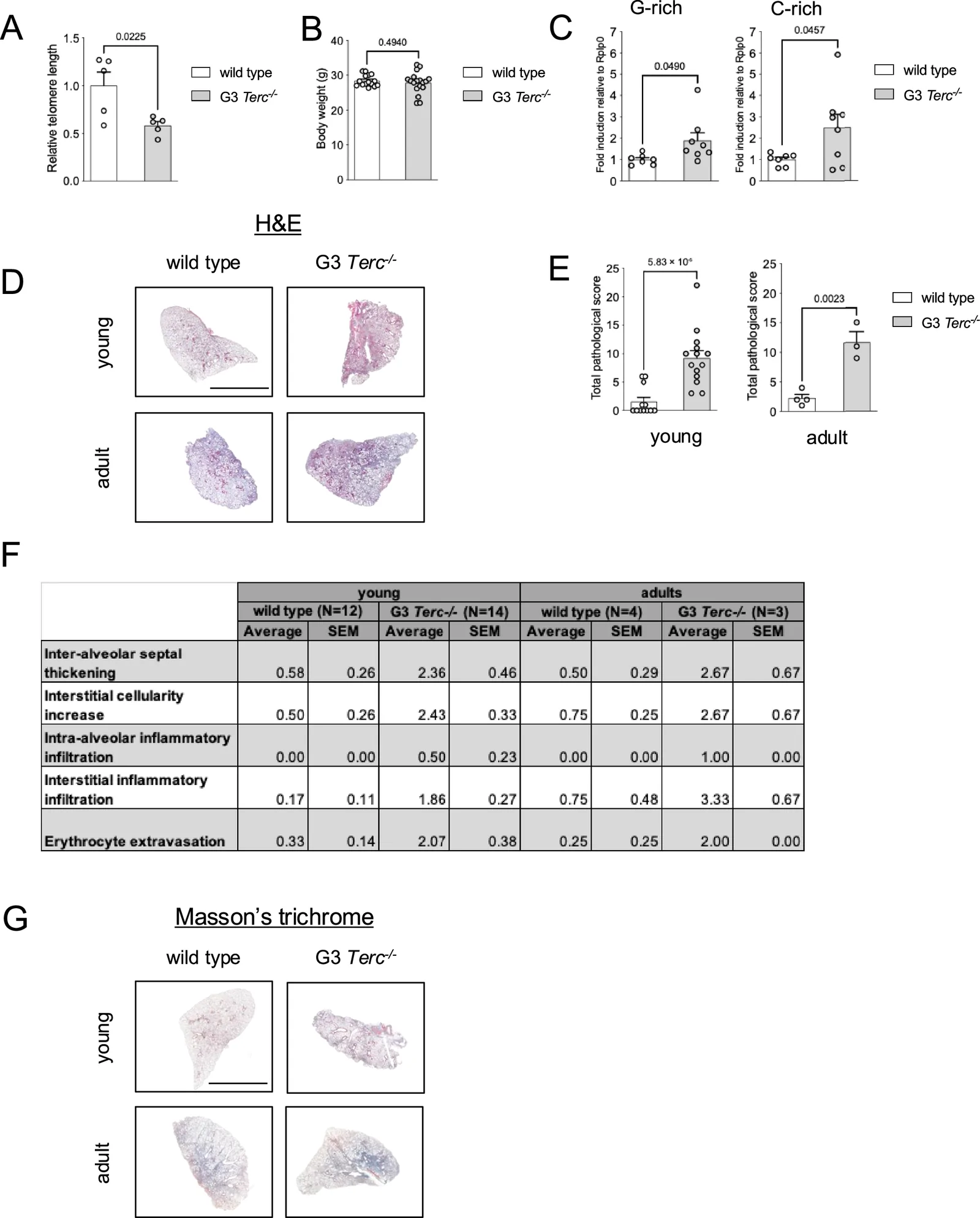
G3 *Terc*^*−/−*^ mice exhibit telomere shortening, increased tdincRNAs, and lung pathology without changes in body weight. (**A**) Lungs from wild-type or G3 *Terc*^*−/−*^ mice were collected at 3 months of age. Relative telomere length in lung samples from mice, as measured by qPCR (*n*  =  5 mice per group). The mouse actin gene was used as a normalizer. (**B**) Body weight of mice of the indicated genotype at 3 months of age (wild-type *n*  =  15 mice, G3 *Terc*^*−/−*^ *n*  =  18 mice). (**C**) Quantification of telomeric damage‑induced non-coding RNAs (tdincRNAs), both G‑rich and C‑rich strands, measured by strand‑specific RT–qPCR, in the lungs of young (5-month-old) mice of the indicated genotype (wild-type *n*  =  7 mice, G3 *Terc*^*−/−*^ *n*  =  8 mice). (**D**) Representative Hematoxylin and Eosin (H&E)-stained whole‑lung sections from mice of the indicated age and genotype. Scale bar, 1000 μm. (**E**, **F**) For each of the indicated morphological parameters evaluated by H&E staining, a score was blindly assigned by a chartered pathologist and used to calculate the total pathological score (young: wild-type *n*  =  12 mice, G3 *Terc*^*−/−*^ *n*  =  14 mice; adult: control *n*  =  4 mice, G3 *Terc*^*−/−*^ *n*  =  3 mice). (**G**) Representative Masson’s Trichrome-stained whole‑lung sections from mice of the indicated age and genotype. Scale bar, 1000 μm. For all graphs, a two-tailed unpaired *t* test was applied. All data are presented as mean ± SEM.

**Figure 1:**
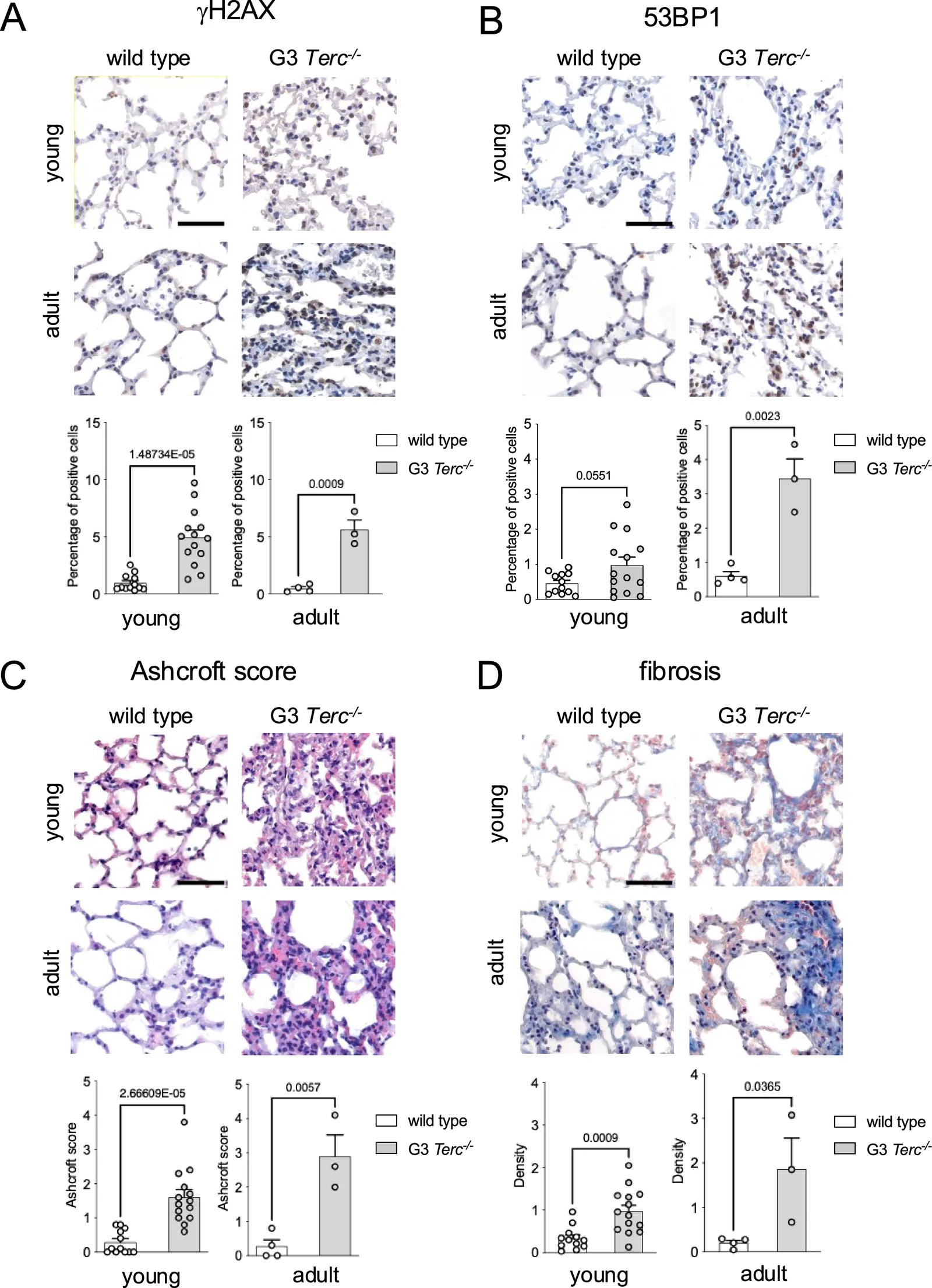
Lungs in G3 *Terc*^*−/−*^ mice show high levels of markers of DDR activation and fibrosis compared to those in age-matched wild-type mice. Lungs of wild-type or G3 *Terc*^*−/−*^ mice were collected at 3 (young) or 12 (adult) months of age. (**A**, **B**) Representative images and quantification of the percentage of γH2AX- or 53BP1-positive cells (brown signal) as detected by immunohistochemistry (IHC) staining and evaluated by image software analysis (young: wild-type *n*  =  12 mice, G3 *Terc*^*−/−*^ *n*  =  14 mice; adult: control *n*  =  4 mice, G3 *Terc*^*−/−*^ *n*  =  3 mice). Original magnification, ×200. Scale bar, 100 µm. (**C**) Representative images and quantitative analyses of the Ashcroft score, evaluated by histopathological analysis of hematoxylin and eosin-stained sections (young: wild-type *n*  =  12 mice, G3 *Terc*^*−/−*^ *n*  =  14 mice; adult: control *n*  =  4 mice, G3 *Terc*^*−/−*^ *n*  =  3 mice). Original magnification, ×200. Scale bar, 100 µm. (**D**) Representative images and quantitative analyses of fibrosis as measured by Masson’s Trichrome staining density (young: wild-type *n*  =  12 mice, G3 *Terc*^*−/−*^ *n*  =  14 mice; adult: control *n*  =  4 mice, G3 *Terc*^*−/−*^ *n*  =  3 mice). Original magnification, ×200. Scale bar, 100 µm. For all graphs, a two-tailed unpaired *t* test was applied. All data are presented as mean ± SEM. Source data are available online for this figure.

In this study, we adopted a mouse model lacking the RNA component of telomerase (*Terc*^*−/−*^) (Blasco et al, 1997). When we analyzed the lungs of the third-generation (G3) of successive intercrosses, they displayed reduced telomere length compared to age-matched wild-type animals (Fig. EV1A) without obvious phenotypic alterations and body weight loss (Fig. EV1B). To study DDR activation, we performed a quantitative, software-aided analysis of in situ immunohistochemistry (IHC) stainings for γH2AX and 53BP1, two distinct markers of DDR activation. When we analyzed lungs from young (3-month-old) and adult (12-month-old) G3 *Terc*^*−/−*^ and wild-type mice, we observed a higher percentage of positive cells for both markers in the lungs of G3 *Terc*^*−/−*^ mice compared to age-matched wild-type animals (Fig. 1A,B). Consistent with the observed telomeric shortening and DDR activation, lungs from G3 *Terc*^*−/−*^ mice had increased levels of telomeric damage-induced non-coding RNAs (tdincRNAs) generated from the transcription of both telomeric DNA strands (Fig. EV1C). Histopathological analyses of hematoxylin and eosin (H&E)-stained sections of the same lung samples, performed in a blinded manner by a certified pathologist, showed increased severity of fibrosis in G3 *Terc*^*−/−*^ mice (Figs. 1C and Fig. EV1D) as evaluated by the semiquantitative modified Ashcroft score (Hübner et al, 2008). In addition, a blinded semiquantitative scoring of individual pathological parameters allowed us to calculate a cumulative pathological score (Fig. EV1E,F), which showed that G3 *Terc*^*−/−*^ mice had a more severe pathology compared to age-matched wild-type mice, with increased interstitial cellularity and thickening of the inter-alveolar septa, associated with overt inflammatory infiltration. Importantly, when we quantified the degree of parenchymal fibrosis by Masson’s Trichrome histochemical staining and software-based density quantification, we measured a substantially higher degree of collagen fiber deposition in lungs of G3 *Terc*^*−/−*^ mice compared to wild-type animals, which is apparent already in young mice (Figs. 1D and  EV1G).

These results indicate that G3 *Terc*^*−/−*^ mice bearing short telomeres have increased lung DDR activation, inflammatory damage, and parenchymal fibrosis.

### tDDR inhibition is effective in the lungs and reduces fibrosis in young mice

Although telomere shortening and dysfunction are associated with IPF, the causative contribution of tDDR to pathogenesis remains currently unexplored. We previously developed telomeric antisense oligonucleotides (tASOs) that, by targeting tdincRNAs, specifically suppress tDDR in vivo in mouse tissues (Rossiello et al, 2017; Aguado et al, 2019).

**Figure EV2:**
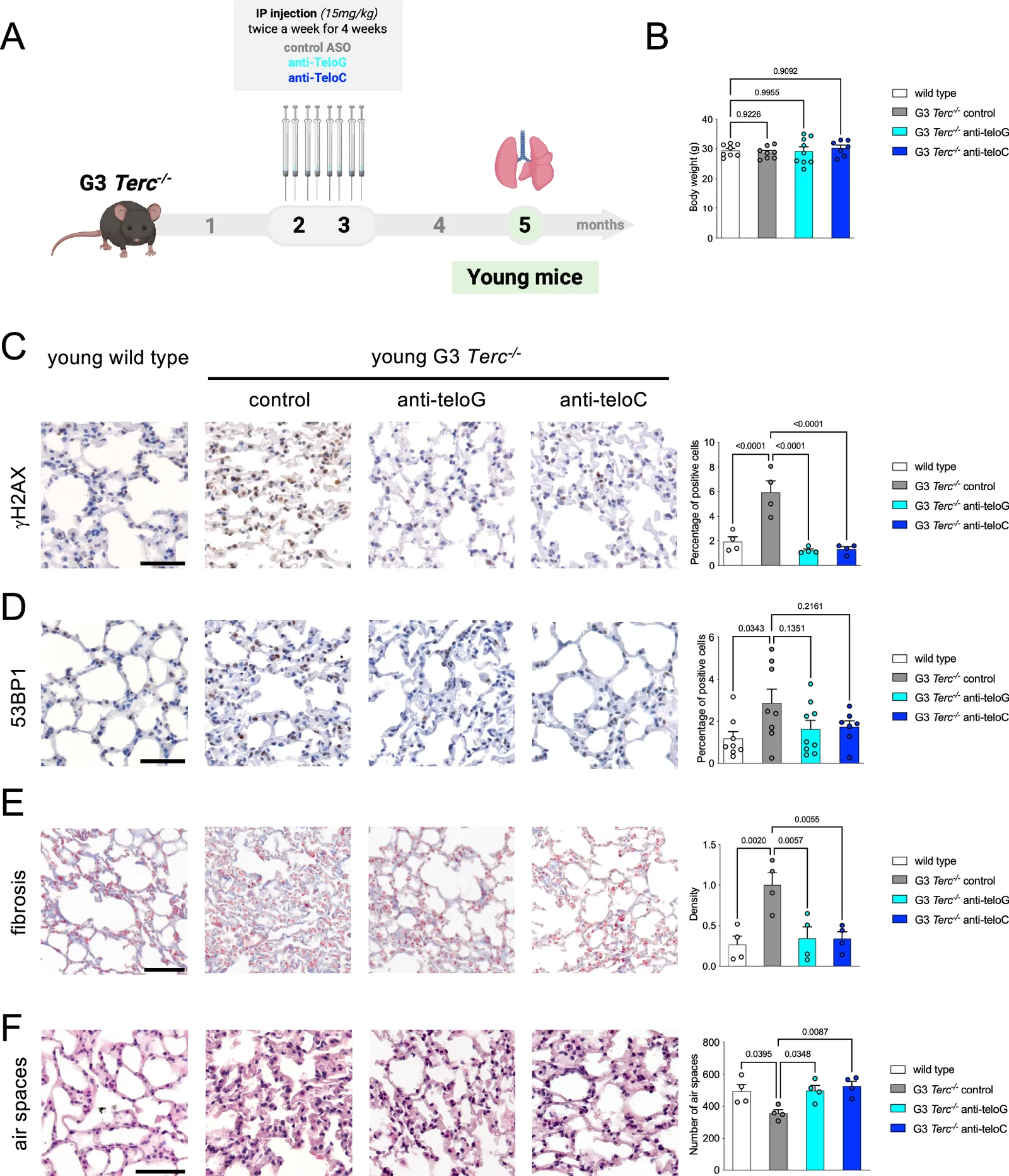
tASOs treatment reduces markers of DDR activation and ameliorates tissue histopathology of the lungs of young G3 *Terc*^*−/−*^ mice. (**A**) Schematic of in vivo ASOs treatments. 2-month-old G3 *Terc*^*−/−*^ mice were intraperitoneally injected with 15 mg/kg of control, anti-teloG, or anti-teloC ASOs twice a week for 4 weeks (eight injections in total), and sacrificed to collect lungs 2 months after the last injection, at 5 (young) months of age, together with untreated age-matched wild-type mice. (**B**) Body weight of mice with the indicated genotype and treatment (wild-type *n*  =  8 mice, G3 *Terc*^*−/−*^ control *n*  =  8 mice; G3 *Terc*^*−/−*^ anti-teloG *n*  = 9 mice, G3 *Terc*^*−/−*^ anti-teloC *n*  = 7 mice). (**C**, **D**) Representative images and quantification of the percentage of γH2AX- or 53BP1-positive cells as detected by IHC staining (γH2AX: wild-type *n*  =  4 mice, G3 *Terc*^*−/−*^ control *n*  =  4 mice; G3 *Terc*^*−/−*^ anti-teloG *n*  =  4 mice, G3 *Terc*^*−/−*^ anti-teloC *n*  =  4 mice; 53BP1: wild-type *n*  =  8 mice, G3 *Terc*^*−/−*^ control *n*  =  8 mice; G3 *Terc*^*−/−*^ anti-teloG *n*  =  9 mice, G3 *Terc*^*−/−*^ anti-teloC *n*  =  7 mice). Original magnification, ×200. Scale bar, 100 µm. (**E**) Representative images and quantification of fibrosis as measured by Masson’s Trichrome staining density (*n* = 4 mice per group). Original magnification, ×200. Scale bar, 100 µm. (**F**) Representative images and quantification of the number of air spaces per field (*n* = 4 mice per group). Original magnification, ×200. Scale bar, 100 µm. For all graphs, ordinary one-way ANOVA followed by Dunnett’s multiple comparison test was applied. All data are presented as mean ± SEM.

To test the impact of tDDR inhibition in an in vivo setting of critically short telomeres, we treated G3 *Terc*^*−/−*^ mice at 2–3 months of age with two independent tASOs (anti-teloG or anti-teloC, complementary to either tdincRNA strand) with intraperitoneal injections twice a week for four weeks. An ASO with an unrelated sequence (control, not complementary to any locus in the mammalian genome) served as a negative control for the treatment. Mice were sacrificed at 5 months of age (young, see schematic in Fig. EV2A) and lung tissues were collected. tASOs treatment was safe and had no significant effect on the overall health or well-being of the animals, as indicated by unaltered body weight (Fig. EV2B). Analyses of lungs collected from young mice revealed reduced levels of γH2AX and 53BP1 DDR markers in tASOs-treated G3 *Terc*^*−/−*^ mice, compared to their controls (Fig. EV2C,D), demonstrating that tASOs can effectively inhibit DDR in the lungs in this model.

To explore the consequences of such reduced DDR signaling on lung fibrosis, we quantified collagen deposition as measured by Masson’s Trichrome staining. We observed reduced lung fibrosis in tASOs-treated mice, compared to their controls, with levels comparable to those of age-matched wild-type mice (Fig. EV2E). The analysis of lung parenchyma architecture of control G3 *Terc*^*−/−*^ mice revealed the destruction of alveolar walls and consequent enlargement of air spaces and number reduction, suggestive of emphysema-like morphological features, which were significantly reduced by tASOs treatment (Fig. EV2F).

These results collectively show that tASOs-mediated tDDR inhibition is effective in the lungs and reduces lung pathology in a genetic model of murine pulmonary fibrosis.

### tDDR inhibition reduces lung fibrosis and pathology in adult mice

**Figure 2:**
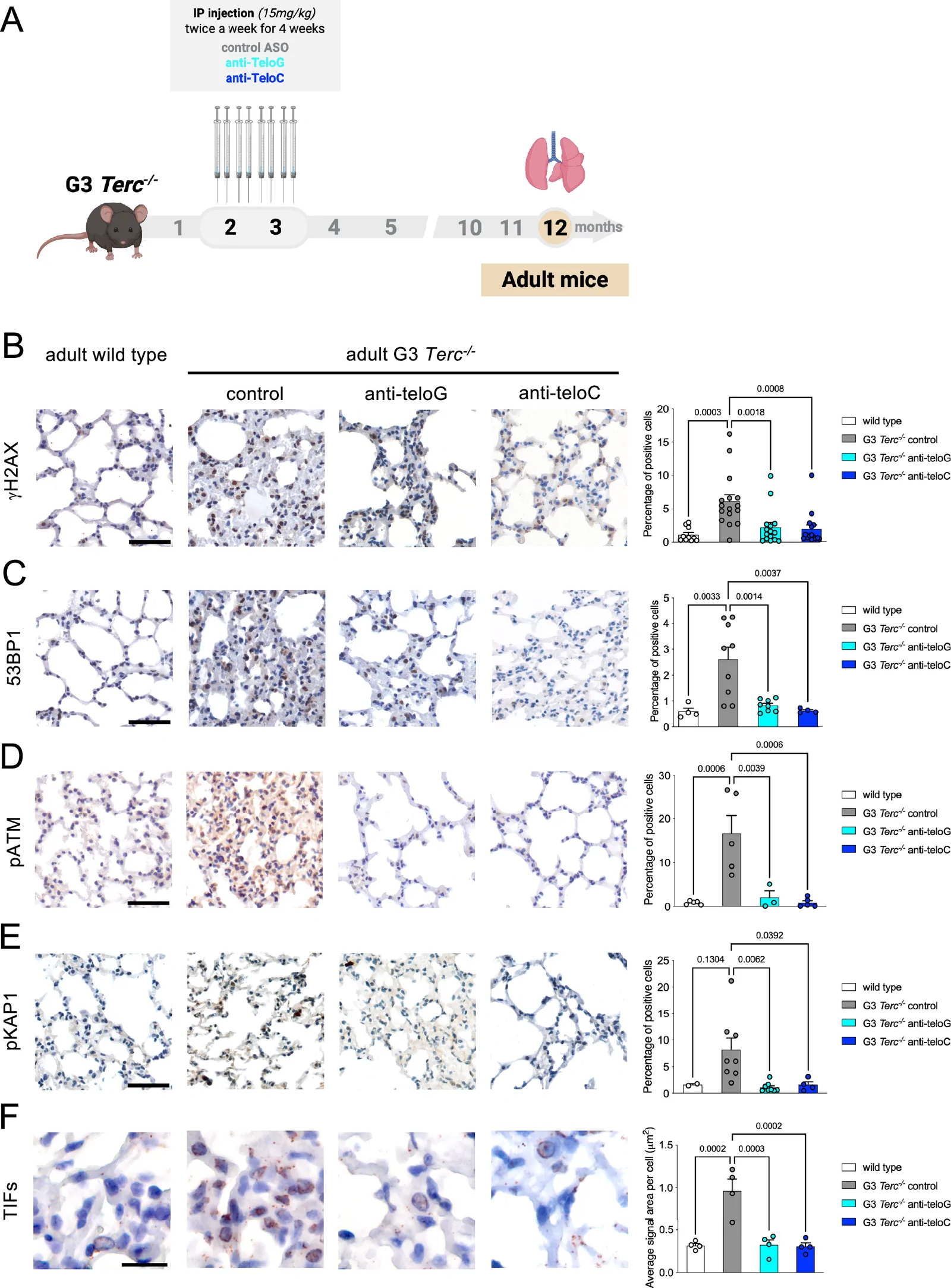
tASOs treatment reduces markers of tDDR activation in the lungs of adult G3 *Terc*^*−/−*^ mice. (**A**) Schematic of in vivo ASOs treatments. 2-month-old G3 *Terc*^*−/−*^ mice were intraperitoneally injected with 15 mg/kg of control, anti-teloG or anti-teloC ASOs twice a week for 4 weeks (eight injections in total), and sacrificed to collect lungs 9 months after the last injection, at 12 (adult) months of age, together with untreated age-matched wild-type mice. (**B**–**E**) Representative images and quantification of the percentage of γH2AX-, 53BP1-, pATM-, or pKAP1-positive cells as detected by IHC staining in the lungs of adult mice treated as indicated (γH2AX: wild-type *n*  =  10 mice, G3 *Terc*^*−/−*^ control *n*  =  16 mice; G3 *Terc*^*−/−*^ anti-teloG *n*  =  15 mice, G3 *Terc*^*−/−*^ anti-teloC *n*  =  15 mice; 53BP1: wild-type *n*  =  4 mice, G3 *Terc*^*−/−*^ control *n*  =  9 mice; G3 *Terc*^*−/−*^ anti-teloG *n*  =  8 mice, G3 *Terc*^*−/−*^ anti-teloC *n*  =  4 mice; pATM: wild-type *n*  =  5 mice, G3 *Terc*^*−/−*^ control *n*  =  5 mice; G3 *Terc*^*−/−*^ anti-teloG *n*  =  3 mice, G3 *Terc*^*−/−*^ anti-teloC *n*  =  5 mice; pKAP1: wild-type *n*  =  2 mice, G3 *Terc*^*−/−*^ control *n*  =  8 mice; G3 *Terc*^*−/−*^ anti-teloG *n*  =  8 mice, G3 *Terc*^*−/−*^ anti-teloC *n*  =  4 mice). Original magnification, ×200. Scale bar, 100 µm. (**F**) Representative images and quantification of telomere-induced foci (TIFs), quantified as average signal area per cell (*n*  =  4 mice per group), detected by chromogenic PLA between γH2AX and TRF2, in the lungs of adult mice treated as indicated. Original magnification, ×400. Scale bar, 30 µm. For all graphs, ordinary one-way ANOVA followed by Dunnett’s multiple comparison test was applied. All data are presented as mean ±  SEM. Source data are available online for this figure.

**Figure EV3:**
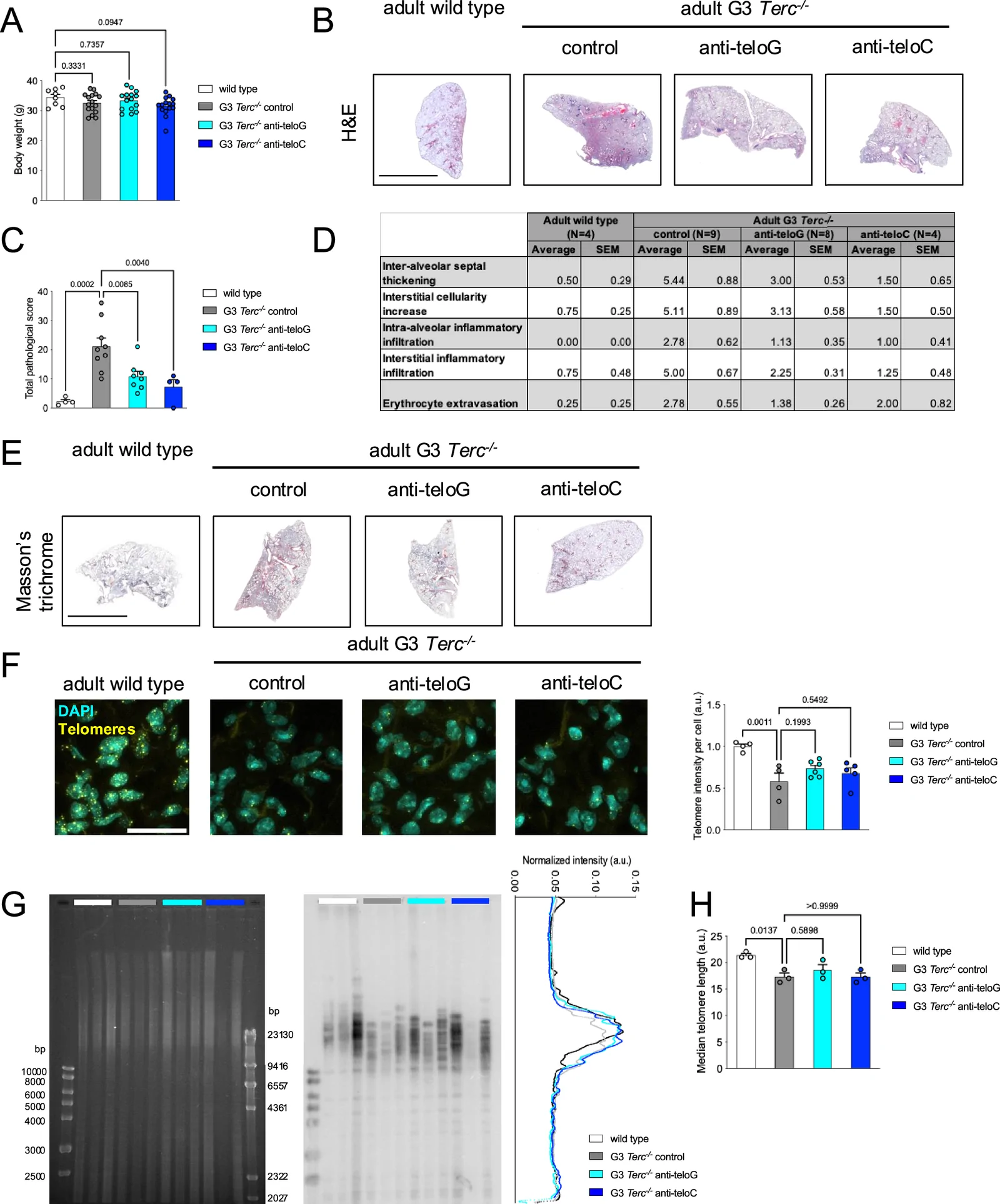
tDDR inhibition ameliorates the pathological score of the lungs of adult G3 *Terc*^*−/−*^ mice without changes in body weight and without affecting telomere length. G3 *Terc*^*−/−*^ mice were treated as described in Fig. 2A and sacrificed at 12 (adult) months of age, together with age-matched wild-type mice. Lungs were collected for analysis. (**A**) Body weight of mice with the indicated genotype and treatment. (wild-type *n*  =  8 mice, G3 *Terc*^*−/−*^ control *n*  =  16 mice; G3 *Terc*^*−/−*^ anti-teloG *n*  =  15 mice, G3 *Terc*^*−/−*^ anti-teloC *n*  =  15 mice). (**B**) Representative Hematoxylin and Eosin (H&E)-stained whole‑lung sections from mice treated as indicated. Scale bar, 1000 μm. (**C**, **D**) For each of the indicated morphological parameters evaluated by H&E, a score was assigned blindly and used to calculate the pathological score (see “Methods” section) (wild-type *n*  = 4 mice, G3 *Terc*^*−/−*^ control *n*  = 9 mice; G3 *Terc*^*−/−*^ anti-teloG *n*  =  8 mice, G3 *Terc*^*−/−*^ anti-teloC *n*  = 4 mice). (**E**) Representative Masson’s trichrome-stained whole‑lung sections from mice treated as indicated. Scale bar, 1000 μm. (**F**) Representative images and quantification of the fluorescence in situ hybridization (FISH) staining and telomeric PNA probe signal quantification expressed in arbitrary units, a.u. (wild-type *n*  =  4 mice, G3 *Terc*^*−/−*^ control *n*  = 4 mice; G3 *Terc*^*−/−*^ anti-teloG *n*  =  6 mice, G3 *Terc*^*−/−*^ anti-teloC *n*  =  5 mice). Scale bar, 20 µm. (**G**) Telomere restriction fragment (TRF) analysis of genomic DNA. Left: ethidium bromide staining showing total DNA. Right: telomere‑specific hybridization using a radiolabeled telomeric probe. The quantification shows the normalized telomeric probe signal intensity (**H**) Median telomere length expressed in arbitrary units (a.u.), as determined from TRF analysis (*n* = 3 mice per group). For all graphs, ordinary one-way ANOVA followed by Dunnett’s multiple comparison test was applied. All data are presented as mean ± SEM.

Since IPF is a progressive disease and most common in adults above 50 years of age, we analyzed in further depth the effects of tDDR inhibition in adult (12-month-old) mice, treated as described above (see schematic in Fig. 2A). Body weight remained unchanged in adult mice following tASOs treatment, consistent with a good long‑term tolerability (Fig. EV3A). Quantitative IHC analyses revealed that tASOs-mediated tDDR inhibition reduced the fraction of cells positive for DDR markers, including γH2AX, 53BP1, pATM, and pKAP1, also in adult mice (Fig. 2B–E). To assess whether tASOs treatment reduced DDR at telomeres specifically, we performed an in situ proximity ligation assay (PLA) between the telomere-specific protein TRF2 and the DDR marker γH2AX on lung sections from adult mice—this approach enables the detection of close spatial proximity between telomeres and activated DDR signaling, indicating the presence of telomere-induced foci (TIFs). Adult G3 *Terc*^*−/−*^ mice displayed a significant increase in TIFs compared with age-matched wild-type controls, indicating tDDR activation in fibrotic lungs. Importantly, tASOs treatment markedly reduced the signals, demonstrating a robust reduction of DDR at telomeres (Fig. 2F), further demonstrating that tASOs effectively inhibit DNA damage signaling at telomeres in adult fibrotic lungs.

**Figure 3:**
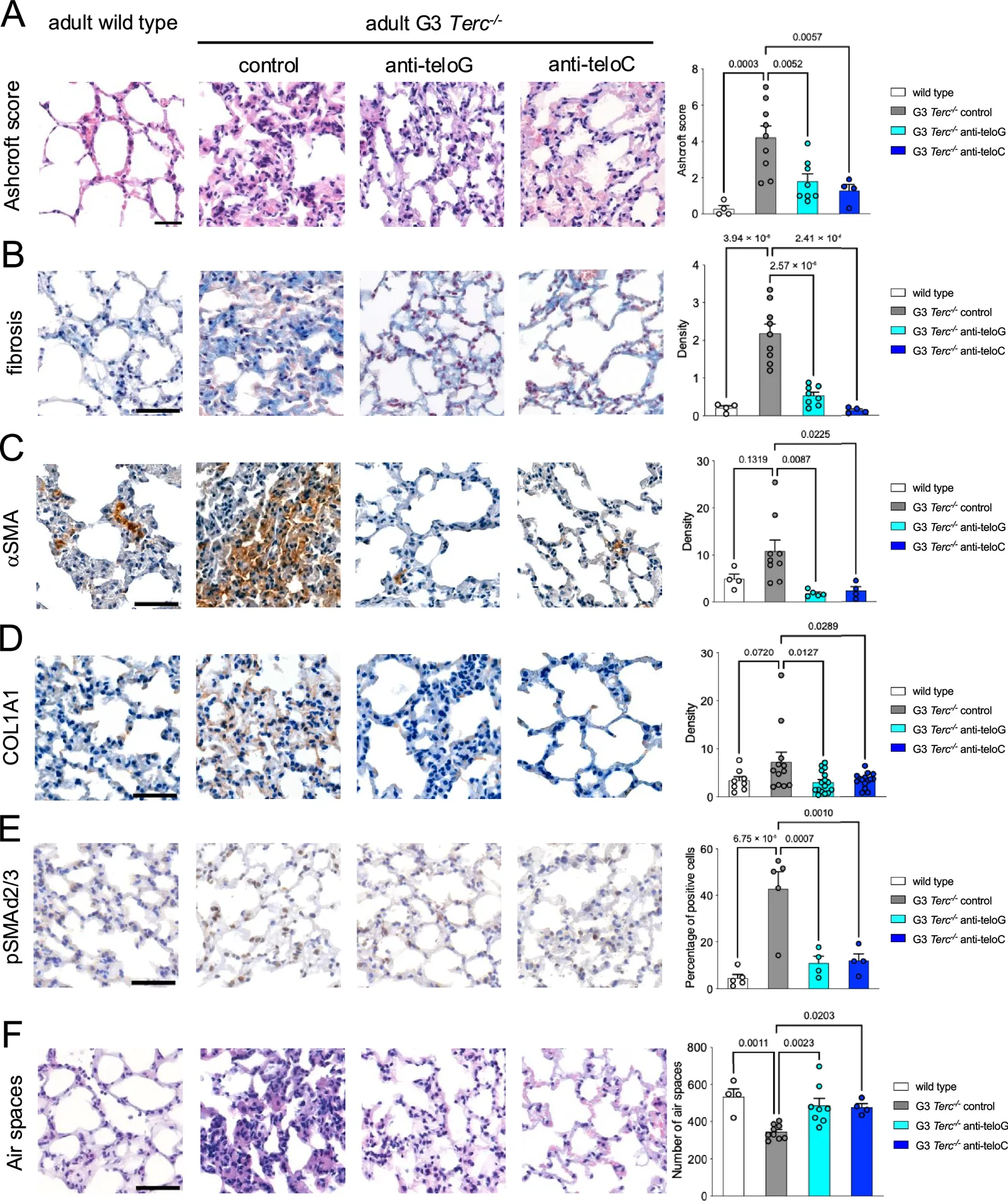
tASOs treatment ameliorates tissue histopathology and fibrosis markers in the lungs of adult G3 *Terc*^*−/−*^ mice. (**A**) Representative images and quantitative analyses of the Ashcroft score in the lungs of adult mice treated as indicated (wild-type *n*  =  4 mice, G3 *Terc*^*−/−*^ control *n*  =  9 mice; G3 *Terc*^*−/−*^ anti-teloG *n*  =  8 mice, G3 *Terc*^*−/−*^ anti-teloC *n*  =  4 mice). Original magnification, x200. Scale bar, 100 µm. (**B**) Representative images and quantitative analysis of fibrosis as measured by Masson’s Trichrome staining density in the lungs of adult mice treated as indicated (wild-type *n*  =  4 mice, G3 *Terc*^*−/−*^ control *n*  =  9 mice; G3 *Terc*^*−/−*^ anti-teloG *n*  =  8 mice, G3 *Terc*^*−/−*^ anti-teloC *n*  =  4 mice). Original magnification, ×200. Scale bar, 100 µm. (**C**–**E**) Representative images and quantification of αSMA density, COL1A1 density and pSMAD2/3-positive cells as detected by IHC staining in the lungs of adult mice treated as indicated (αSMA: wild-type *n*  =  4 mice, G3 *Terc*^*−/−*^ control *n*  =  9 mice; G3 *Terc*^*−/−*^ anti-teloG *n*  =  5 mice, G3 *Terc*^*−/−*^ anti-teloC *n*  =  4 mice; COL1A1: wild-type *n*  =  8 mice, G3 *Terc*^*−/−*^ control *n*  =  12 mice; G3 *Terc*^*−/−*^ anti-teloG *n*  =  16 mice, G3 *Terc*^*−/−*^ anti-teloC *n*  =  15 mice; pSMAD2/3: wild-type *n*  =  5 mice, G3 *Terc*^*−/−*^ control *n*  =  5 mice; G3 *Terc*^*−/−*^ anti-teloG *n*  =  4 mice, G3 *Terc*^*−/−*^ anti-teloC *n*  =  4 mice). Original magnification, ×200. Scale bar, 100 µm. (**F**) Representative images and quantification of the number of air spaces per field in the lungs of adult mice treated as indicated (wild-type *n*  =  4 mice, G3 *Terc*^*−/−*^ control *n*  =  9 mice; G3 *Terc*^*−/−*^ anti-teloG *n*  =  8 mice, G3 *Terc*^*−/−*^ anti-teloC *n*  =  4 mice). Original magnification, ×200. Scale bar, 100 µm. (**A**, **B**) The wild-type group data are the same as in Fig. 1, and were included here for comparison. For all graphs, Ordinary one-way ANOVA followed by Dunnett’s multiple comparison test was applied. All data are presented as mean ± SEM. Source data are available online for this figure.

Consistent with the observed reduction of DDR activation, tASOs treatment resulted in a substantial decrease in lung parenchymal damage, as shown by the reduction of both Ashcroft score and the pathological score (Figs. 3A and  EV3B–D). Importantly, tASOs-treated mice displayed a significant reduction in fibrosis, as supported by decreased Masson’s Trichrome staining (Figs. 3B and  EV3E). Since extracellular matrix (ECM) deposition by activated myofibroblasts is a central driver of fibrotic progression (Wynn, 2008), we assessed alpha-smooth muscle actin (α-SMA) expression, a marker of myofibroblast activation. Consistent with attenuated fibrogenesis, tASOs-treated mice exhibited a lower level of α-SMA (Fig. 3C), indicating reduced myofibroblast activation. Similarly, quantification of COL1A1 signal by immunohistochemistry revealed a significant reduction in collagen-producing cells in tASOs-treated mice (Fig. 3D), in line with the decreased extracellular matrix deposition. Consistent with attenuation of profibrotic signaling, the percentage of phosphorylated SMAD2/3 (pSMAD2/3)-positive cells, a readout of profibrotic Transforming Growth Factor-beta (TGF-β) signaling in IPF (Fernandez and Eickelberg, 2012), was also reduced following tASOs treatment (Fig. 3E). As a consequence, tASOs-treated mice also showed improved lung architecture with a significant reduction of emphysema-like features (Fig. 3F).

In summary, treatment with two distinct tASOs is consistently effective in tDDR inhibition in adult mice and leads to significantly reduced lung fibrosis and pathology, without altering telomere length in lung cells, as demonstrated by quantitative FISH and telomere restriction fragment analyses (Fig. EV3F–H).

### tDDR inhibition reverses telomere dysfunction-induced aging-associated transcriptional changes

**Figure 4:**
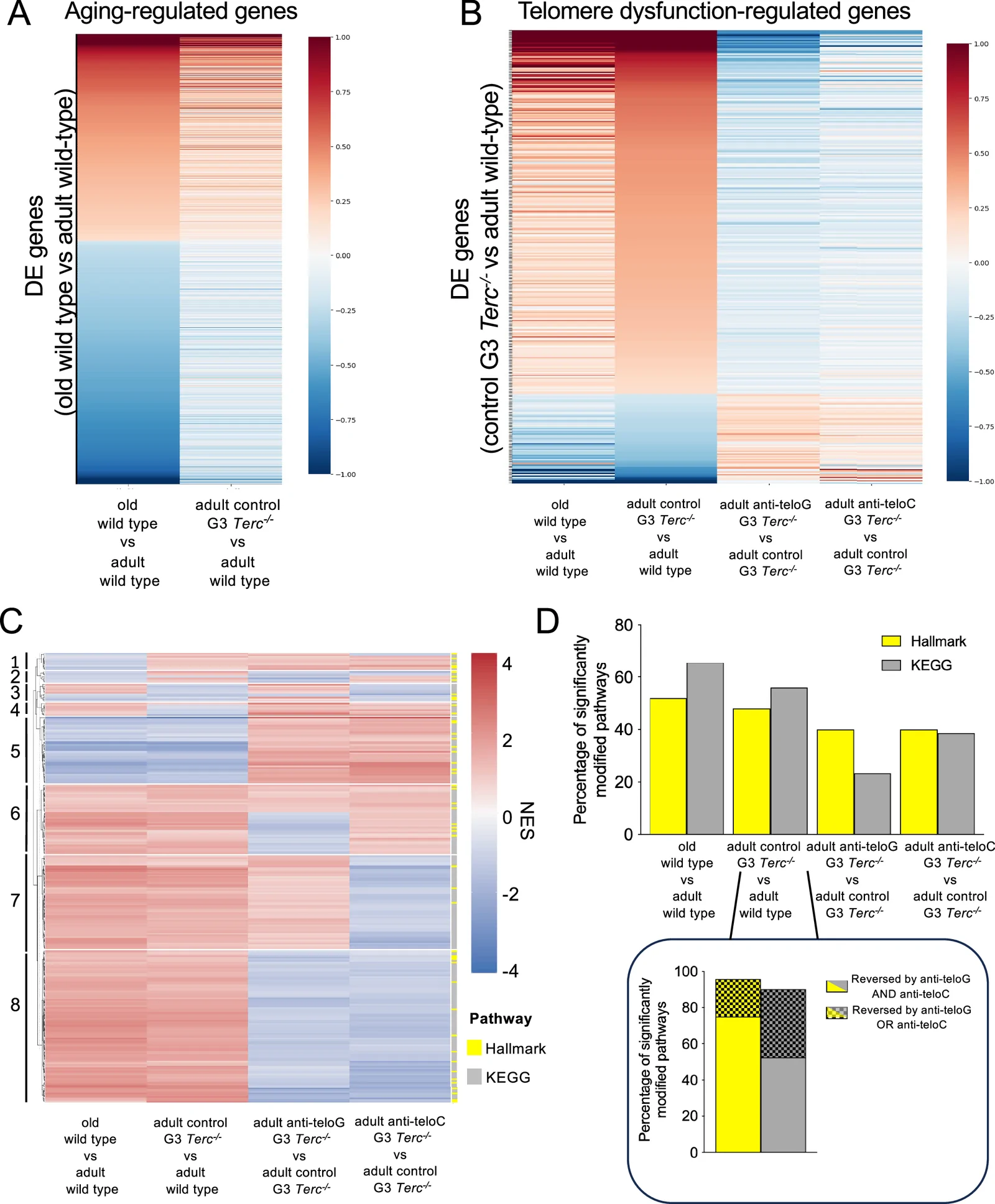
Telomere dysfunction drives an aged-like transcriptional profile in lungs, which is reversed by tDDR inhibition. G3 *Terc*^*−/−*^ mice were treated as in Fig. 2A and sacrificed at 12 (adult) months of age; untreated wild-type mice were sacrificed at 12 (adult) or 22 (old) months of age, and lungs were collected (old wild-type *n* = 3 mice, adult wild-type *n* = 5 mice, control G3 *Terc*^*−/−*^ *n* = 5 mice, anti-teloG G3 *Terc*^*−/−*^ *n* = 5 mice, anti-teloC G3 *Terc*^*−/−*^ *n* = 5 mice). (**A**) Heatmap of log fold-change (logFC) gene expression level of significantly differentially expressed genes associated with aging (*n* = 1350 genes with adjusted *P* value < 0.05 for the old vs adult wild-type groups) for the indicated comparisons. (**B**) Heatmap of log fold-change (logFC) gene expression level of significantly differentially expressed genes associated with telomere dysfunction (*n* = 332 genes with adjusted *P* value < 0.05 for the control G3 *Terc*^*−/−*^ vs adult wild-type groups) for the indicated comparisons. (**C**) Heatmap of the Normalized Enrichment Score for KEGG (yellow) and Hallmark (gray) pathways, for the indicated comparisons. After a hierarchical and k-means clustering, eight clusters were observed (indicated by numbers on the left). (**D**) Significance of pathway deregulation (KEGG are in gray, Hallmark in yellow). Top, percentage of significantly modified pathways (*P* value adjusted <0.05) for each comparison; bottom, the percentage of significantly deregulated pathways in control G3 *Terc*^*−/−*^ vs adult wild-type mice, which are reversed in anti-teloG and/or anti-teloC vs control G3 *Terc*^*−/−*^ mice.

To broaden our investigations, we examined the transcriptional landscape of lungs from G3 *Terc*^*−/−*^ mice. Given the increased incidence of IPF among the elderly and the fibrotic phenotypes reported in the lungs of aged mice (Piñeiro-Hermida et al, 2020), we included in our analyses lungs of aged wild-type mice. We therefore analyzed lungs from adult (12-month-old) wild-type mice and old (22-month-old) wild-type mice using Illumina RNA sequencing (RNA-seq), and we compared them. We identified a set of 1350 genes with a significant differential expression based on mean log2-fold change, defining a lung aging-associated transcriptional signature that reflects age-driven transcriptional remodeling (Figs. 4A, first column and  EV4A, left; Dataset EV1). Next, we examined how the expression of these same genes varied in control G3 *Terc*^*−/−*^ compared to aged-matched wild-type mice (Fig. 4A, second column). Strikingly, the expression profiles in these two comparisons were highly consistent, indicating that telomere shortening in this model recapitulates, in an accelerated manner, the gene expression changes observed during normal aging.

**Figure EV4:**
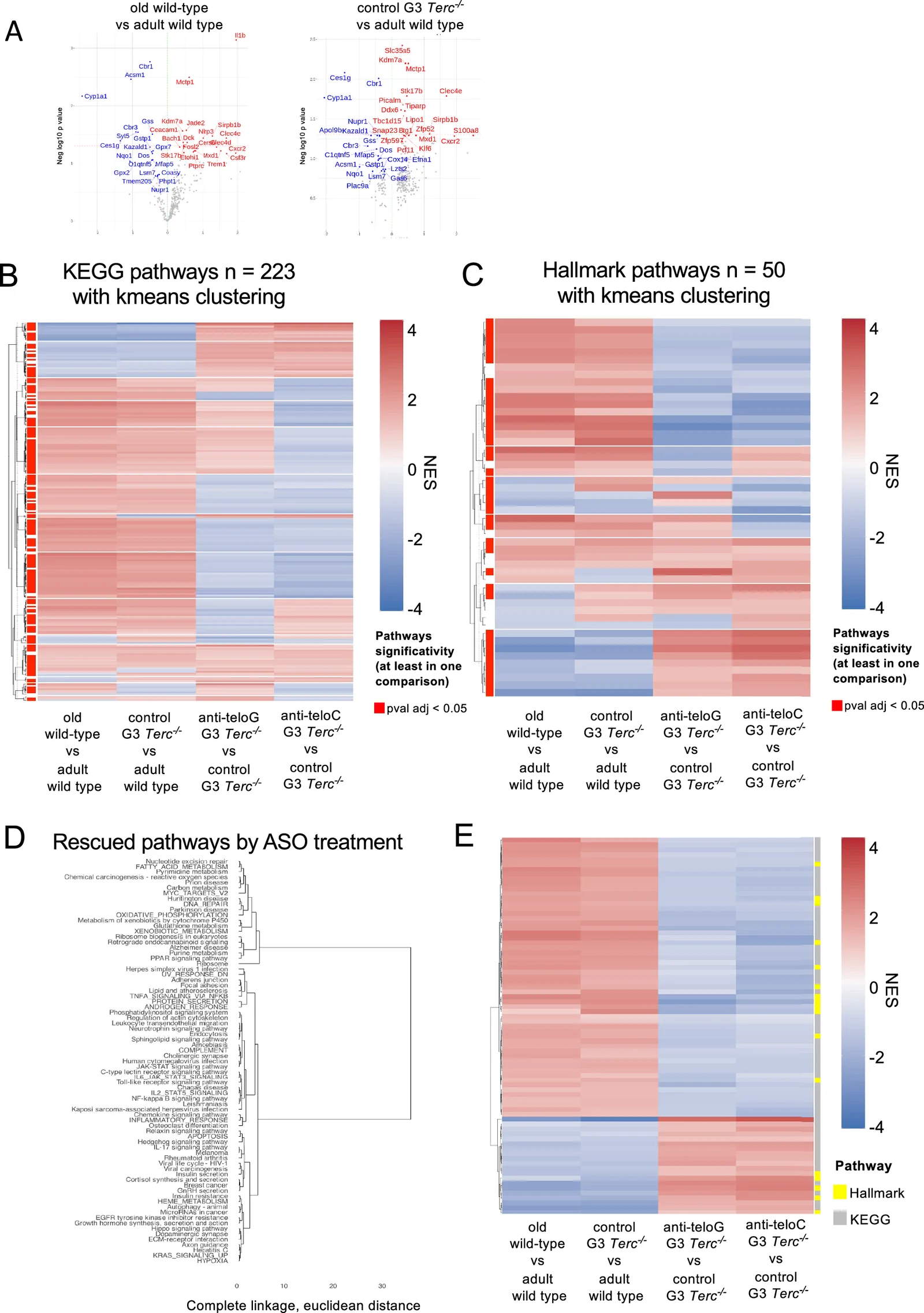
tDDR inhibition reverses the altered transcriptional profiles of lungs of adult G3 *Terc*^*−/−*^ mice. G3 *Terc*^*−/−*^ mice were treated as described in Fig. 2A and sacrificed at 12 (adult) months of age; untreated wild-type mice were sacrificed at 12 (adult) or 22 (old) months of age. Lungs were collected for analysis. (**A**) Volcano plots highlighting the top 20 upregulated and downregulated genes in the indicated comparisons (logFC and *P* value were calculated with DESeq2 using the Wald test). (**B**, **C**) Heatmaps of the Normalized Enrichment Score for KEGG (**B**, *n* = 223) and Hallmark (**C**, *n* = 50) pathways. Pathway enrichment analysis for the indicated comparisons, and a hierarchical clustering and k-means (*k* = 14) clustering were performed. Pathways were annotated on the left in red when significantly modified (*P* value adjusted by Benjamini-Hochberg test) in at least one comparison. (**D**, **E**) Dendogram and heatmap of the pathways (Hallmark: yellow, uppercase; KEGG: gray, lowercase) reversed by tASOs treatments. A reversed pathway is a pathway significantly deregulated (*P* value adjusted <0.05) in old wild-type vs adult wild-type mice and reversed in anti-teloG G3 *Terc*^*−/−*^ vs control G3 *Terc*^*−/−*^ and anti-teloC G3 *Terc*^*−/−*^ vs control G3 *Terc*^*−/−*^ (*n* = 3 mice for old wild-type, *n* = 5 mice for adult wild-type, *n* = 5 mice for control G3 *Terc*^*−/−*^, *n* = 5 mice for anti-teloG G3 *Terc*^*−/−*^, *n* = 5 mice for anti-teloC G3 *Terc*^*−/−*^).

By a complementary approach, we compared adult control G3 *Terc*^*−/−*^ and adult wild-type mice, identifying a subset of 332 genes with a significant differential expression, which we defined as a telomere dysfunction-associated transcriptional signature (Figs. 4B, second column and  EV4A, right; Dataset EV2). Using this signature as a reference, we then compared its expression pattern with the changes observed between old and adult wild‑type mice, and we observed that the two profiles were highly similar (Fig. 4B, first two columns). Excitingly, both independent tASOs treatments reversed the expression of most of these genes (Fig. 4B, last two columns).

**Figure EV5:**
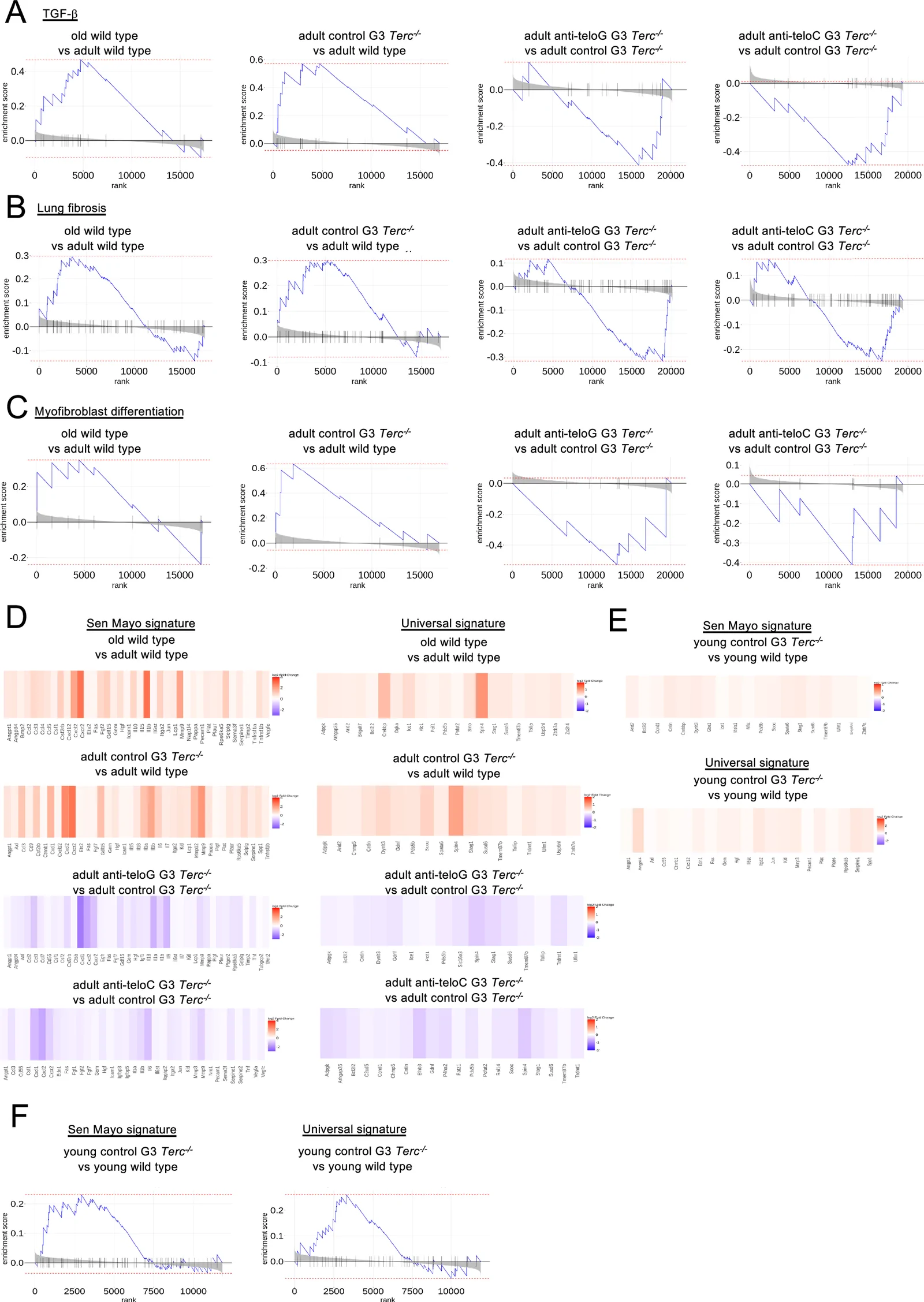
tDDR inhibition reverses fibrosis and senescence signatures of lungs of adult G3 *Terc*^*−/−*^ mice. (**A**–**C**) GSEA enrichment plot for the TGF-β, lung fibrosis, and myofibroblast differentiation pathways for the indicated comparisons. (**D**, **E**) Differential Gene Expression (logFC) of Sen Mayo and Universal signature. (**F**) GSEA enrichment plots for the Sen Mayo and Universal signatures for the indicated comparisons.

In order to identify the biological pathways and processes affected, we performed a pathway analysis followed by an unsupervised clustering on the Normalized Enrichment Score using KEGG and Hallmark pathway sets. We observed remarkably similar profiles when comparing old wild-type to adult wild-type mice, and adult control G3 *Terc*^*−/−*^ to adult wild-type mice, across both pathway sets (Fig. 4C, first two columns), further supporting the notion that telomere dysfunction alters pathways that are similarly deregulated in normal aging. Importantly, these pathway changes were largely reversed following tDDR inhibition by either tASOs treatment (Fig. 4C, last two columns). In particular, among the different clusters, cluster 5 and cluster 8 exhibited similar pathway patterns in old wild-type and control G3 *Terc*^*−/−*^ mice compared to adult wild-type mice, and were reversed by tASOs treatments—a pathway was considered reversed if it was significantly modified in control G3 *Terc*^*−/−*^ mice compared to wild-type mice and regulated in the opposite direction upon treatment (Fig. 4C; clustering for KEGG and Hallmark separately is shown in Fig. EV4B,C). Quantification of these analyses revealed that more than 80% of pathways were significantly reversed by one or both tASOs treatments (Figs. 4D and  EV4D,E). In particular, gene set enrichment analysis (GSEA) of pathways related to fibrosis (Strunz et al, 2020; Panagiotidis et al, 2025; Lingampally et al, 2025; El Agha et al, 2017), revealed upregulation of TGF-β signaling, lung fibrosis, and myofibroblast differentiation pathways in the lungs of aged wild-type and control G3 *Terc*^*−/−*^ mice and tDDR inhibition by either tASO effectively reversed these changes (Fig. EV5A–C). Collectively, these results indicate that telomere dysfunction in G3 *Terc*^*−/−*^ mice recapitulates the transcriptional changes observed in naturally aged lungs, including fibrosis-related pathways, supporting the notion that telomere shortening phenocopies accelerated lung aging. tDDR inhibition counteracts age-associated changes in gene expression and pathways and overall promotes a rejuvenated transcriptional profile in G3 *Terc*^*−/−*^ mice.

### Aging and telomere dysfunction cause a cellular senescence phenotype, increased inflammation, and immune activation in a tDDR-dependent manner

**Figure 5:**
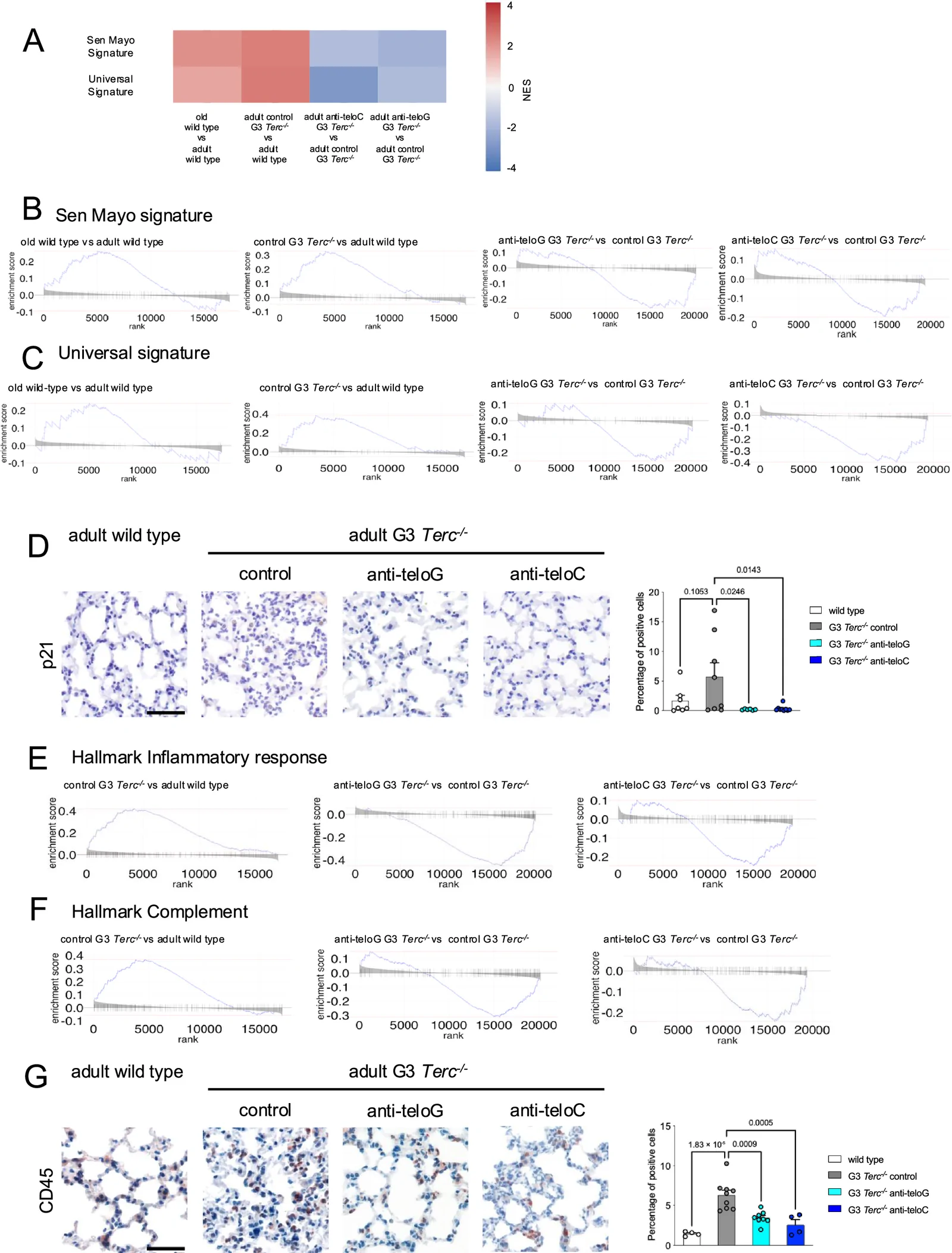
tDDR inhibition reduces cellular senescence, inflammation, and immune activation in the lungs of G3 *Terc*^*−/−*^ mice. G3 *Terc*^*−/−*^ mice were treated as in Fig. 2A and sacrificed at 12 (adult) months of age, untreated wild-type mice were sacrificed at 12 (adult) or 22 (old) months of age, and lungs were collected (old wild-type *n* = 3 mice, adult wild-type *n* = 5 mice, control G3 *Terc*^*−/−*^ *n* = 5 mice, anti-teloG G3 *Terc*^*−/−*^ *n* = 5 mice, anti-teloC G3 *Terc*^*−/−*^ *n* = 5 mice). (**A**) Heatmap of the Normalized Enrichment Score of Senescence Pathways (Sen Mayo and Universal Signature) for indicated comparisons. (**B**, **C**) GSEA enrichment plots for the senescence Sen Mayo and Universal signatures for the indicated comparisons. (**D**) Representative images and quantification of the percentage of p21-positive cells (brown signal) as detected by immunohistochemistry (IHC) staining and evaluated by image software analysis (wild-type *n*  =  7 mice, G3 *Terc*^*−/−*^ control *n*  =  8 mice; G3 *Terc*^*−/−*^ anti-teloG *n*  =  6 mice, G3 *Terc*^*−/−*^ anti-teloC *n*  =  9 mice). Original magnification, ×200. Scale bar, 100 µm. Ordinary one-way ANOVA followed by Dunnett’s multiple comparison test was applied. Data are presented as mean ± SEM. (**E**, **F**) GSEA enrichment plots for the Hallmark Inflammatory response and Complement pathways for the indicated comparisons. (**G**) Representative images and quantification of the percentage of CD45-positive cells (brown signal) as detected by immunohistochemistry (IHC) staining and evaluated by image software analysis (wild-type *n*  =  4 mice, G3 *Terc*^*−/−*^ control *n*  =  9 mice; G3 *Terc*^*−/−*^ anti-teloG *n*  =  8 mice, G3 *Terc*^*−/−*^ anti-teloC *n*  =  4 mice). Original magnification, ×200. Scale bar, 100 µm. Ordinary one-way ANOVA followed by Dunnett’s multiple comparison test was applied. Data are presented as mean ± SEM. Source data are available online for this figure.

Cellular senescence, a permanent state of growth arrest elicited by protracted DDR activation, involves a profound transcriptional rewiring of gene expression. Leveraging from two defined signatures of cellular senescence, the Sen Mayo signature and the Universal signature (Hernandez-Segura et al, 2017a; Saul et al, 2022), we probed our RNA-seq dataset, and we observed an enrichment for both of these signatures in lungs from old wild-type mice compared to adult ones (Figs. 5A–C and  EV5D), thus showing that cellular senescence increases in aging lungs and validating our datasets. Also, adult control G3 *Terc*^*−/−*^ compared to age-matched wild-type mice displayed an enrichment for both signatures (Figs. 5A–C and  EV5D), indicating that telomere‑driven DNA damage precipitates senescence‑associated transcriptional changes observed in naturally aged lungs—notably, senescence‑associated transcriptional programs were already elevated in the lungs of young G3 *Terc*^*−/−*^ mice (Fig. EV5E,F). Most importantly, treatments of G3 *Terc*^*−/−*^ mice with either tASO reversed both senescence signatures (Figs. 5A–C and  EV5D). Consistent with these observations, quantitative IHC of p21 (Cdkn1a)—a cyclin‑dependent kinase inhibitor and key mediator of the DNA damage-induced cell‑cycle arrest response—revealed an increased percentage of positive cells in control G3 *Terc*^*−/−*^ lungs, which was reduced by tASOs treatment (Fig. 5D).

**Figure EV6:**
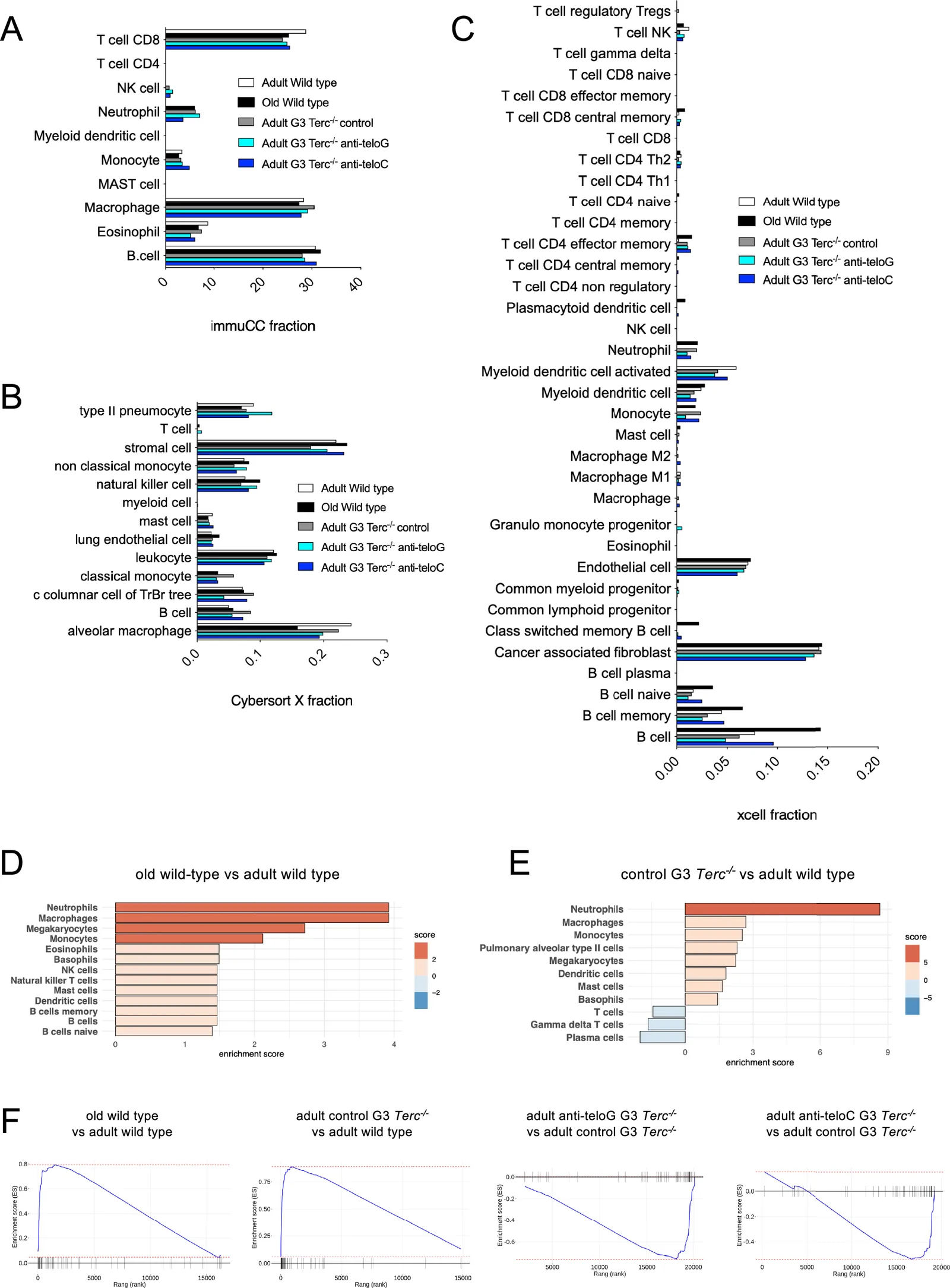
Neutrophil transcriptional signatures are elevated in old and G3 *Terc*^*−/−*^ lungs and reduced by tASOs. (**A**–**C**) Average deconvolution fractions across samples. Bars represent mean deconvolution scores within each group (old wild-type, adult wild-type, control G3 *Terc*^*−/−*^, anti-teloG G3 *Terc*^*−/−*^, and anti-teloC G3 *Terc*^*−/−*^). (**D**) Enriched cytotypes from the rank-based gene set enrichment analysis (GSEA, clusterProfiler, permutation-based enrichment test) on old wild-type and adult wild-type. Bar length reflects the significance level (log10 adjusted *P* value). The significant cytotypes are all positively associated with the old wild-type condition. (**E**) Enriched cytotypes from the rank-based gene set enrichment analysis (GSEA, clusterProfiler, permutation-based enrichment test) on old wild-type and control G3 *Terc*^*−/−*^ compared to adult wild-type. Bar length reflects the significance level (log10 adjusted *P* value). (**F**) GSEA plots showing the Neutrophils associations across different DEG lists. Neutrophils are positively enriched in old wild-type and control G3 *Terc*^*−/−*^ samples (both versus adult wild-type). This is in contrast with the downregulation of neutrophil-related transcriptional activity observed in the anti-teloG G3 *Terc*^*−/−*^, and anti-teloC G3 *Terc*^*−/−*^ (both versus control G3 *Terc*^*−/−*^).

Cellular senescence is known to promote tissue inflammation. So-called inflammaging is a chronic, low-grade inflammation that develops with aging and age-related diseases, driven by the accumulation of senescent cells, pro-inflammatory signaling, and immune dysregulation (Desai et al, 2018; Franceschi et al, 2000). To explore whether these inflammatory features were reflected in our transcriptomic data, we next inferred cell‑type composition across experimental groups. Cell‑type deconvolution analyses revealed comparable estimated proportions (Fig. EV6A–C) with, notably, increased neutrophil‑associated signatures, consistent with an enhanced inflammatory state, in old wild‑type and control G3 *Terc*^*−/−*^ lungs, which were reduced by tASOs treatment (Fig. EV6D–F).

We next examined gene expression profiles specifically associated with the inflammatory response and complement pathways, and we observed that their upregulation in the lungs of control G3 *Terc*^*−/−*^ mice was reversed by tDDR inhibition with either tASO (Fig. 5E,F), indicating tDDR as a key driver of activation of these pathways. To independently support these conclusions, we measured immune cell infiltration in the lungs through quantitative IHC with the pan-leukocyte marker CD45. A significantly higher percentage of immune cells was detected in the lungs of control G3 *Terc*^*−/−*^ mice, confirming increased immune activation and inflammation, which was significantly reduced by tDDR inhibition (Fig. 5G).

In summary, transcriptomic and immunodetection analyses supported increased levels of cellular senescence, inflammation, and immune cell recruitment in the lungs of G3 *Terc*^*−/−*^ mice, and the efficacy of tDDR inhibition in lowering them.

### tDDR inhibition by tASOs is also effective in Alveolar Type II cells and restores lung cell population homeostasis

**Figure 6:**
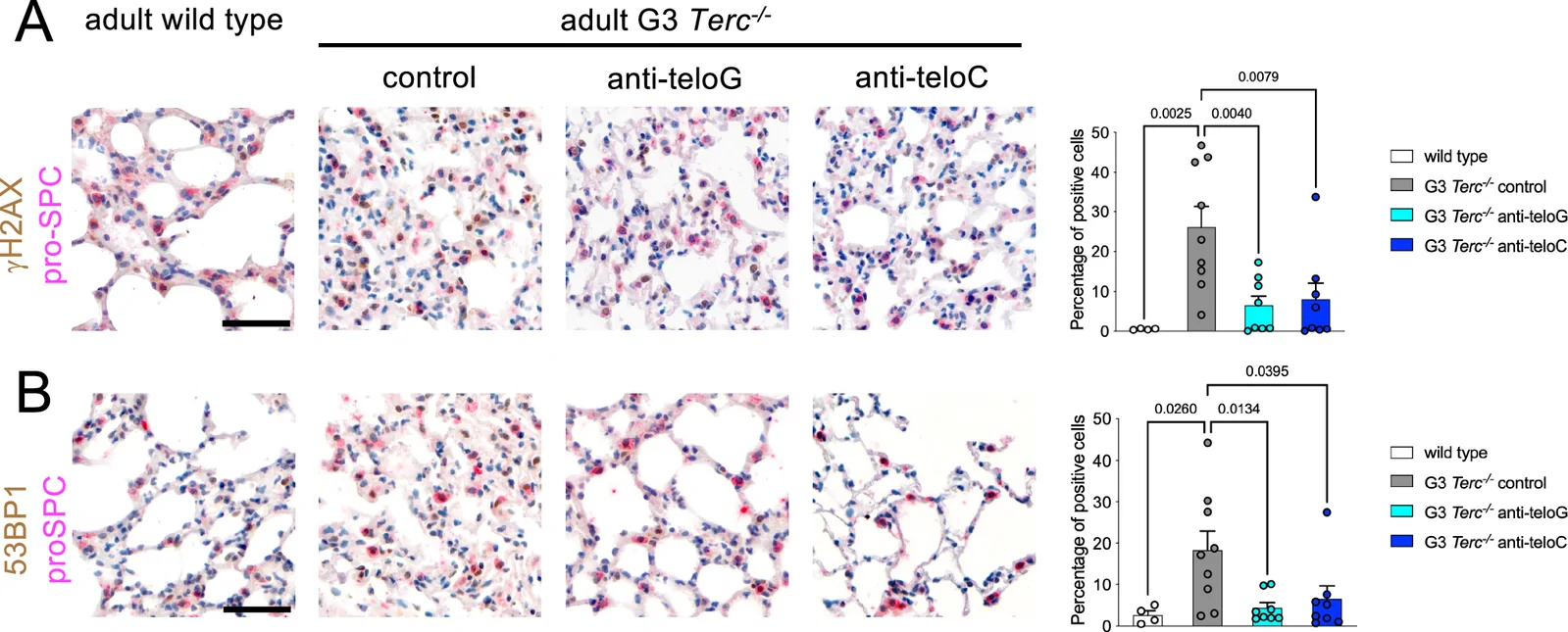
tASOs treatment reduces markers of DDR activation in ATII cells. G3 *Terc*^*−/−*^ mice were treated as in Fig. 2A and sacrificed at 12 (adult) months of age, in parallel with age-matched wild-type controls. Lungs were collected and stained for prosurfactant protein C (pro-SPC) to label alveolar type II (ATII) cells. Representative images and quantification of the percentage of γH2AX- (**A**) or 53BP1- (**B**) positive cells (brown signal) among all ATII-positive cells (pink signal), as determined by double IHC staining (wild-type *n*  =  4 mice, G3 *Terc*^*−/−*^ control *n*  =  9 mice; G3 *Terc*^*−/−*^ anti-teloG *n*  =  8 mice, G3 *Terc*^*−/−*^ anti-teloC *n*  =  8 mice). Original magnification, ×200. Scale bar, 100 µm. For all graphs, ordinary one-way ANOVA followed by Dunnett’s multiple comparison test was applied. All data are presented as mean ± SEM. Source data are available online for this figure.

**Figure EV7:**
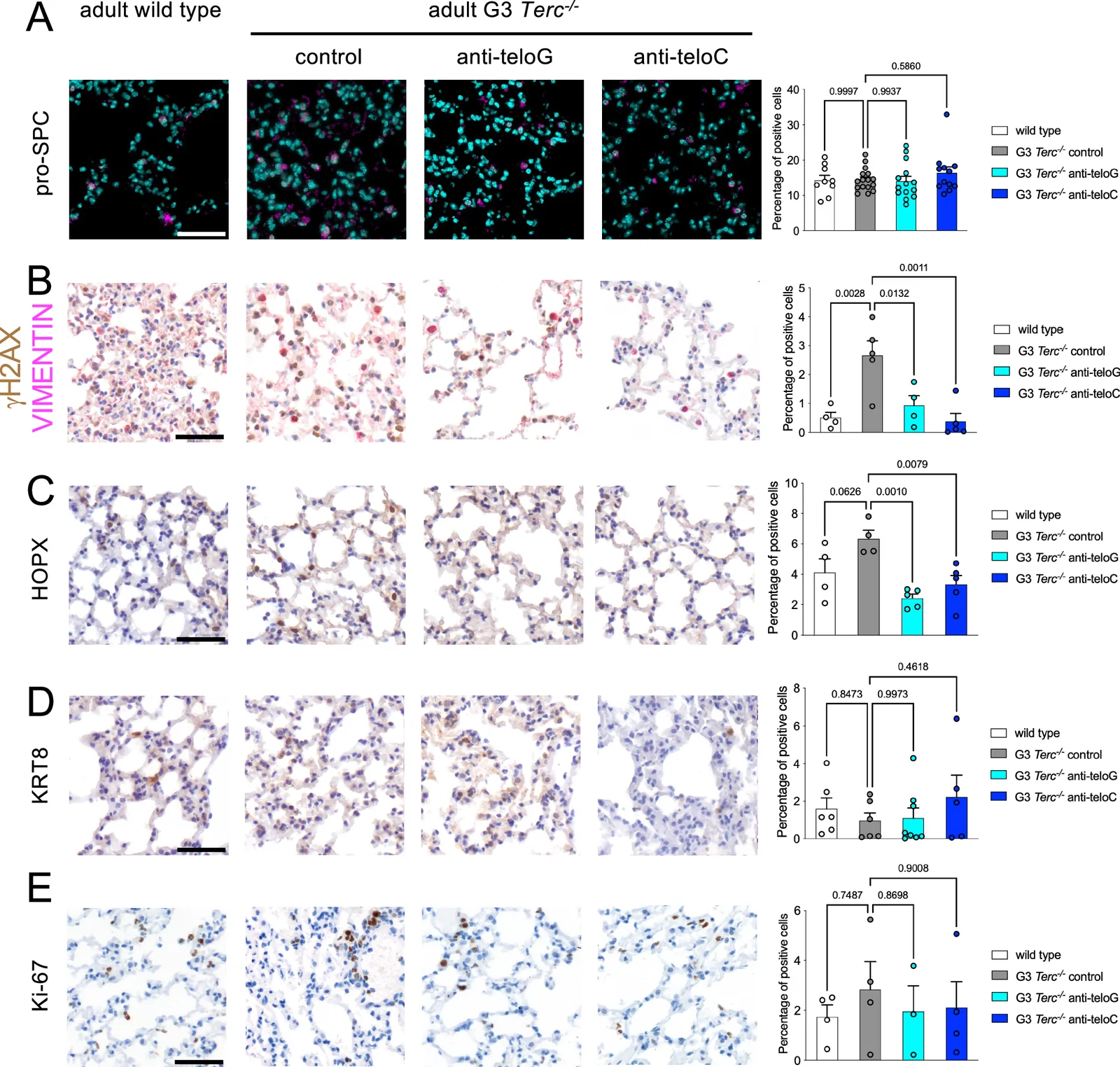
tASOs treatment attenuates DDR also in mesenchymal cells and promotes restoration of alveolar homeostasis. (**A**) Representative images and quantification of the percentage of ATII cells, as detected by the pro-SPC marker by immunofluorescence (wild-type *n*  = 9 mice, G3 *Terc*^*−/−*^ control *n*  = 16 mice; G3 *Terc*^*−/−*^ anti-teloG *n*  =  14 mice, G3 *Terc*^*−/−*^ anti-teloC *n*  = 12 mice). (**B**) Representative images and quantification of the percentage of γH2AX-positive cells (brown signal) among all VIMENTIN-positive cells (pink signal), as determined by double IHC staining (wild-type *n*  =  4 mice, G3 *Terc*^*−/−*^ control *n*  = 5 mice; G3 *Terc*^*−/−*^ anti-teloG *n* = 4 mice, G3 *Terc*^*−/−*^ anti-teloC *n* = 5 mice). (**C**–**E**) Representative images and quantification of the percentage of HOPX-, KRT8-, or Ki-67-positive cells as detected by IHC staining in the lungs of adult mice treated as indicated (HOPX: wild-type *n*  =  4 mice, G3 *Terc*^*−/−*^ control *n*  =  4 mice; G3 *Terc*^*−/−*^ anti-teloG *n*  =  5 mice, G3 *Terc*^*−/−*^ anti-teloC *n*  =  5 mice; KRT8: wild-type *n*  =  6 mice, G3 *Terc*^*−/−*^ control *n*  =  6 mice; G3 *Terc*^*−/−*^ anti-teloG *n*  =  8 mice, G3 *Terc*^*−/−*^ anti-teloC *n*  =  5 mice; KI67: wild-type *n*  =  4 mice, G3 *Terc*^*−/−*^ control *n*  =  4 mice; G3 *Terc*^*−/−*^ anti-teloG *n*  =  3 mice, G3 *Terc*^*−/−*^ anti-teloC *n*  =  4 mice). Original magnification, ×200. Scale bar, 100 µm. For all graphs, ordinary one-way ANOVA followed by Dunnett’s multiple comparison test was applied. All data are presented as mean ± SEM.

Alveolar type II (ATII) cells are considered lung stem/progenitor cells with a key role in lung function and regeneration (Barkauskas et al, 2013). Dysfunction in this cell type is emerging as a driving mechanism of IPF pathogenesis (Confalonieri et al, 2022). Indeed, induction of telomere dysfunction selectively in ATII cells has been reported to be sufficient to increase lung fibrosis susceptibility (Alder et al, 2015; Povedano et al, 2015; Liu et al, 2019). To assess the impact of tDDR inhibition by tASOs treatment on this crucial cell population, ATII cells were labeled in lung sections with antibodies against the prosurfactant protein C (pro-SPC) and probed for markers of DDR activation. We observed a significant increase in the percentage of γH2AX- and 53BP1-positive ATII cells in the lungs of control G3 *Terc*^*−/−*^ mice compared to age-matched wild-type animals (Fig. 6A,B), with no significant differences in their total number (Fig. EV7A). Notably, tASOs-treated G3 *Terc*^*−/−*^ mice showed a strong reduction in the fraction of γH2AX- and 53BP1-positive cells (Fig. 6A,B). Similarly, VIMENTIN-positive mesenchymal cells, a population that contributes to extracellular matrix deposition and is frequently activated during fibrosis (Guo et al, 2025; Bell and Belz, 2026), showed increased γH2AX levels in G3 *Terc*^*−/−*^ mice lungs and decreased ones upon tASOs treatments (Fig. EV7B), indicating tASOs' effectiveness in a broader cellular context, beyond the progenitor compartment.

Alveolar type I (ATI) cells, identified by HOPX, represent the terminally differentiated epithelial population responsible for gas exchange and derive from ATII cells during tissue repair (Aspal and Zemans, 2020). We observed that the percentage of ATI cells was higher in G3 *Terc*^*−/−*^ mice, pointing to a shift toward aberrant differentiation often linked to defective progenitor function. tASOs treatment reversed this increase (Fig. EV7C), indicating a restoration of epithelial homeostasis and supporting the notion that reducing tDDR in ATII cells prevents maladaptive differentiation trajectories. In contrast, the frequency of transitional KRT8-positive cells, an intermediate progenitor-like population that emerges during alveolar regeneration and can expand under chronic injury (Aspal and Zemans, 2020), remained unchanged across conditions (Fig. EV7D). Similarly, the proportion of proliferating Ki-67-positive cells did not vary significantly between groups (Fig. EV7E), indicating that the observed effects on epithelial populations are not driven by changes in proliferation but rather by modulation of DDR and differentiation programs.

These results indicate that tASOs treatment is efficacious to reduce DDR in different lung cell populations, including ATII and mesenchymal cells, and to restore alveolar cell differentiation and homeostasis.

### tDDR activation and lung pathology are strongly correlated

**Figure 7:**
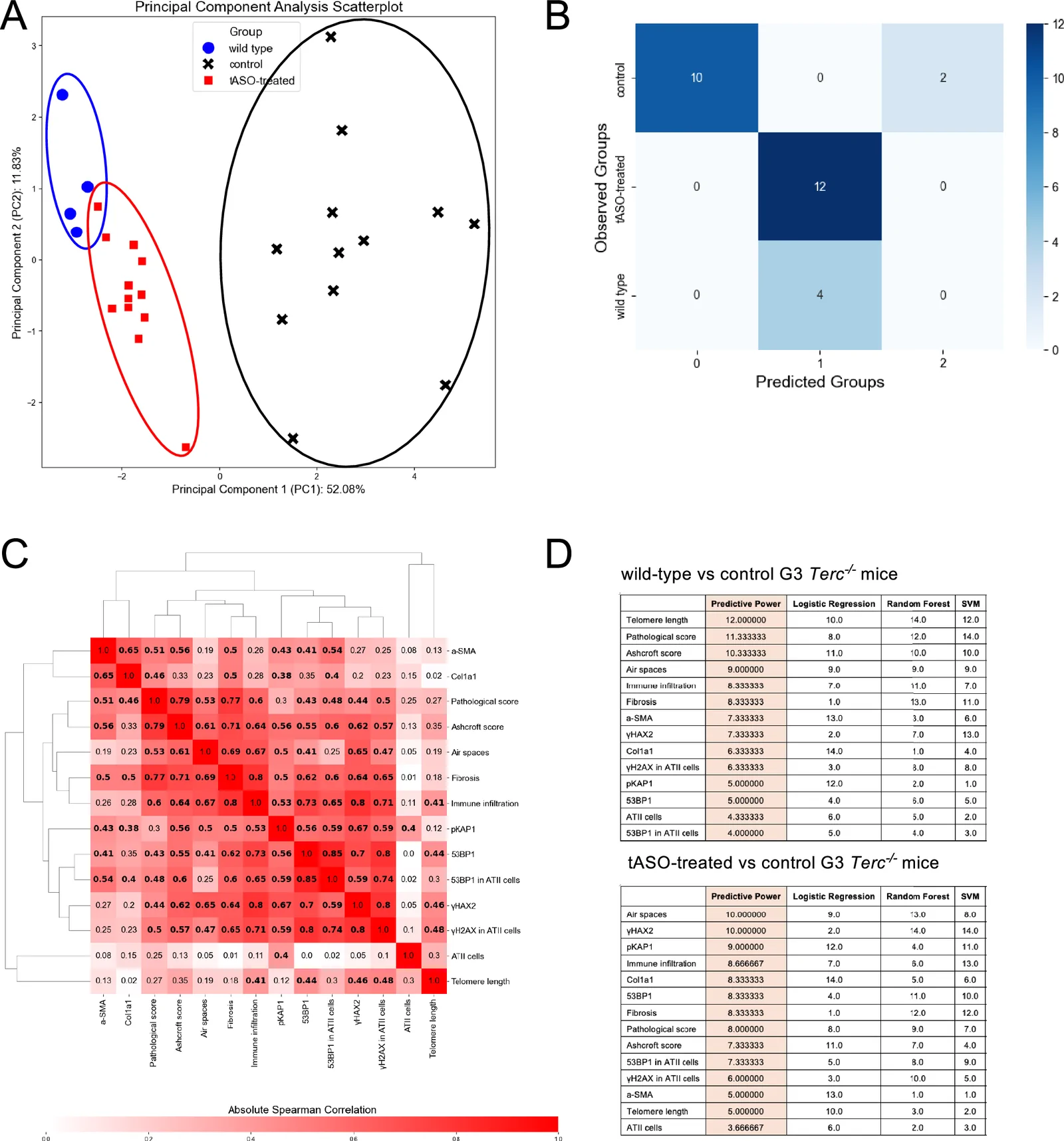
Multivariate analysis shows group separation driven by DDR and pathology markers. In all, 28 adult (12-month-old) mice scored for 14 variables were used for the following analysis. (**A**) Multivariable principal component analysis (PCA). The 2 axes explained 63.91% of the variance (52.08% PC1 and 11.83% PC2). Based on PCA, the mice segregated into three groups (blue: wild-type mice, black: control G3 *Terc*^*−/−*^ mice, red: tASOs-treated G3 *Terc*^*−/−*^ mice). (**B**) Heatmap of the confusion matrix for the aggregative clustering classification of the mice (observed groups vs predicted groups). (**C**) Correlation heatmap of the variables with hierarchical clustering. The correlation was calculated using the absolute Spearman score between each couple of variables (in bold *P* value < 0.05). (**D**) Tables of the power of each variable to predict sample classification using the different models shown (logistic regression, random forest, and SVM). The predictive power is the weighted average of the accuracy of each model.

To determine the different effects of tDDR inhibition on the parameters described above, we used the data obtained with adult mice—wild-type, control- and tASOs-treated G3 *Terc*^*−/−*^ mice—as described in Fig. 2A (see Dataset EV3) for a principal component analysis (PCA). The first two components of the PCA explained respectively 52.08% and 11.83% of the variance, indicating a strong correlation between the variables. The first component was able to separate the three groups: wild-type, control (untreated and control ASO-treated G3 *Terc*^*−/−*^ mice), and tASOs-treated (anti-teloG- and anti-teloC-treated G3 *Terc*^*−/−*^ mice), confirming their biological difference with respect to the variables measured (Fig. 7A). Notably, the wild-type and control mouse groups were clearly separated, while the treated group was closer to the wild-type group, indicating that tDDR inhibition shifts the parameters studied toward a wild-type phenotype. A similar result is evident in a heatmap showing the comparison between the predicted groups generated through agglomerative clustering and the observed groups (Fig. 7B). Wild-type and control mice were clearly separated in agglomerative clustering, while tASOs-treated and wild-type groups clustered together in the same predicted group, indicating that tASOs-treated mice were more similar to the wild-type condition.

**Figure EV8:**
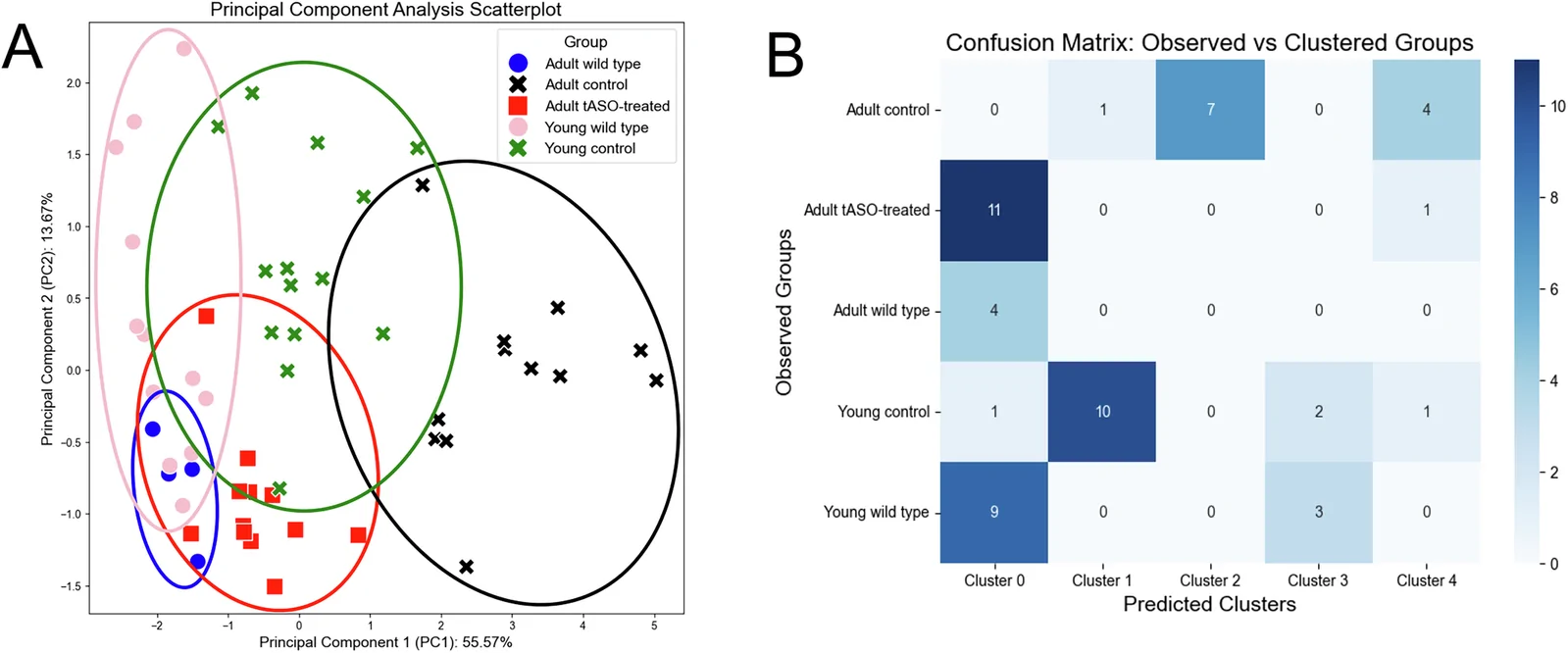
PCA of multiparametric features shows a shift toward a youthful state following tASOs treatment. In all, 28 adult (12-month-old) and 26 young (3-month-old) mice scored for 7 variables were used for the following analysis. (**A**) Multivariable principal component analysis (PCA). The 2 axes explained 69.24% of the variance (55.57% PC1 and 13.67% PC2). Based on PCA, the mice were segregated into 5 groups (blue: adult wild-type mice, black: adult control G3 *Terc*^*−/−*^ mice, red: adult tASOs-treated G3 *Terc*^*−/−*^ mice, pink: young wild-type mice, green: young G3 *Terc*^*−/−*^mice). (**B**) Heatmap of the confusion matrix for the aggregative clustering classification of the mice (observed groups vs predicted groups).

The inclusion of data from wild-type and G3 *Terc*^*−/−*^ young mice (Dataset EV4) in such PCA analysis indicated that tASOs-treated adult mice shift their multiparametric profiles toward the younger groups, partially overlapping with them, indicating a rejuvenation effect of tASOs treatment (Fig. EV8A,B).

Next, to visualize the relationships between all the variables studied above, we constructed a correlation heatmap. A simple agglomerative hierarchical clustering, using absolute Spearman correlation to quantify their distance, highlighted different groups of variables (Fig. 7C). Those related to the DDR (γH2AX, γH2AX in ATII cells, 53BP1, 53BP1 in ATII cells, and pKAP1) closely correlated to each other, and clustered together; similarly, tissue pathological markers (Ashcroft score, pathological score, air spaces, fibrosis, immune infiltrate, αSMA and COL1A1), also generated a compact cluster. These observations that different variables, describing the same biological phenomenon but measured by different techniques, show a high degree of association, strengthen our conclusions. Interestingly, the DDR and the tissue pathology clusters were the closest ones as indicated by the dendrogram, highlighting their tight relationship. Moreover, the strong correlation between the extent of DDR activation and lung pathology was supported by the significant scores (as shown in bold) between most variables from these two clusters.

Next, all variables studied were ranked based on their predictive power, measuring the ability to predict group belonging of each mouse studied (Fig. 7D). Interestingly, telomere length is the best predictor (first position in the ranking) in distinguishing wild-type from control groups. The observation that, when comparing tASOs-treated with control groups, telomere length loses predictive power further supports the notion that tASOs treatment uncouples telomere length from tDDR activation and lung pathology.

Taken together, these results strengthen the conclusion that tDDR inhibition normalizes the molecular and pathological features in the lungs, and emphasize the association between tDDR, rather than telomere length, and lung pathology.

### tDDR inhibition alleviates established fibrosis and emphysema in old G3 *Terc*^*−/−*^ mice

**Figure 8:**
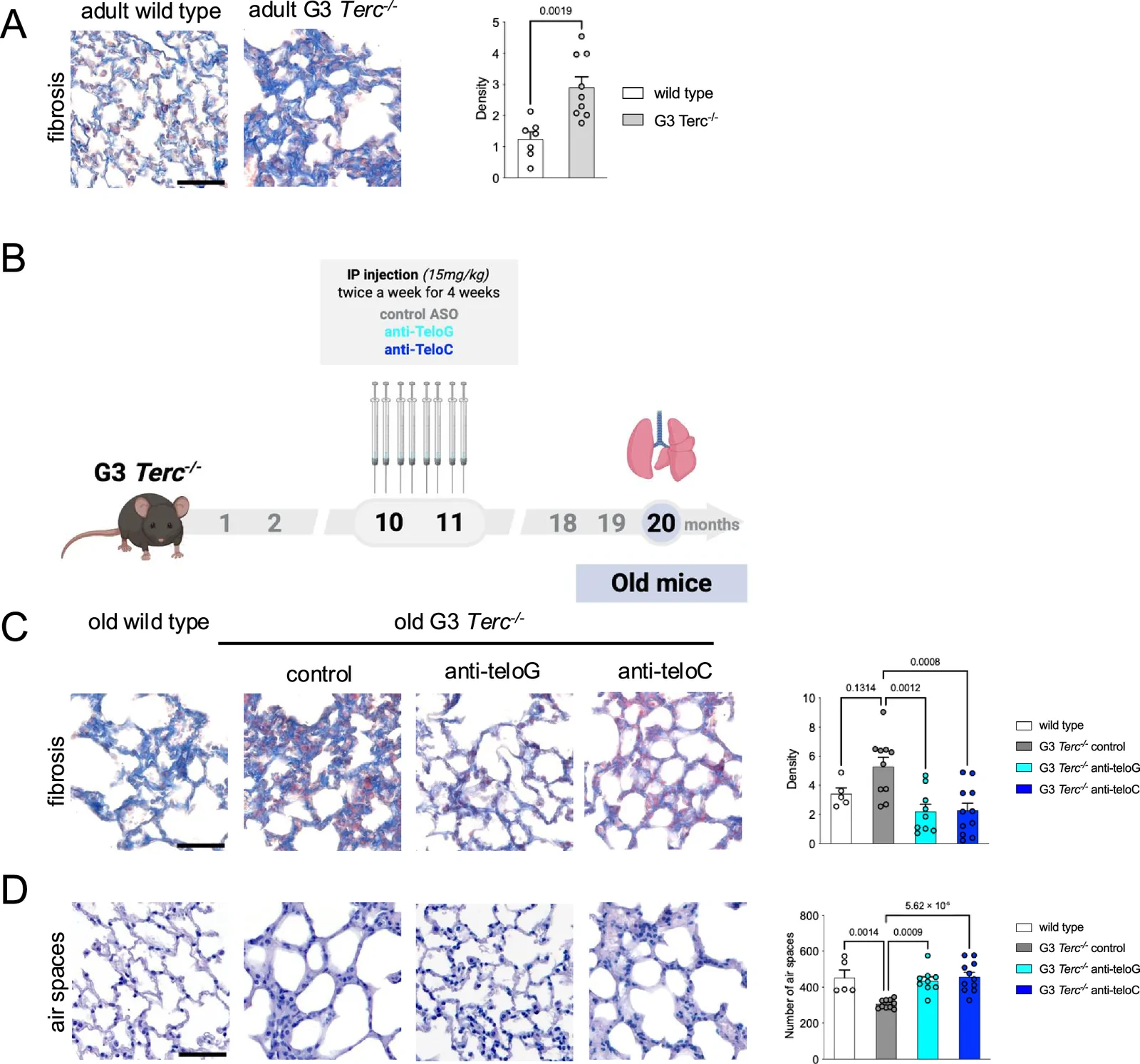
tDDR inhibition in old mice reduces lung fibrosis and ameliorates tissue histology. (**A**) Lungs from wild-type or G3 *Terc*^*−/−*^ mice were collected at 10 months of age. Representative images and quantitative analyses of fibrosis as measured by Masson’s Trichrome staining density (wild-type *n*  =  7 mice, G3 *Terc*^*−/−*^ *n*  =  9 mice). Original magnification, ×200. Scale bar, 100 µm. A two-tailed unpaired *t* test was applied. Data are presented as mean ± SEM. (**B**) Schematic of ASO in vivo treatment. Ten-month-old (adult) G3 *Terc*^*−/−*^ mice were intraperitoneally injected with 15 mg/kg of the indicated ASOs twice a week for 4 weeks (eight injections in total), and sacrificed 9 months after the treatment, at 20 (old) months of age, together with age-matched wild-type controls. Lungs were collected for analysis. (**C**) Representative images and quantitative analysis of fibrosis as detected by Masson’s Trichrome staining density (wild-type *n*  =  5 mice, G3 *Terc*^*−/−*^ control *n*  =  10 mice; G3 *Terc*^*−/−*^ anti-teloG *n*  =  9 mice, G3 *Terc*^*−/−*^ anti-teloC *n*  =  11 mice). Original magnification, ×200. Scale bar, 100 µm. Ordinary one-way ANOVA followed by Dunnett’s multiple comparison test was applied. Data are presented as mean ± SEM. (**D**) Representative images and quantification of the number of air spaces per field (wild-type *n*  =  5 mice, G3 *Terc*^*−/−*^ control *n*  =  10 mice; G3 *Terc*^*−/−*^ anti-teloG *n*  =  9 mice, G3 *Terc*^*−/−*^ anti-teloC *n*  =  11 mice). Original magnification, ×200. Scale bar, 100 µm. Ordinary one-way ANOVA followed by Dunnett’s multiple comparison test was applied. Data are presented as mean ± SEM. Source data are available online for this figure.

Since IPF is an age-related disease that progresses over time, we evaluated the impact of tDDR inhibition by tASOs on lung fibrosis and structure in old animals, thus with a longer-established and more severe fibrosis. As expected, adult (10-month-old) G3 *Terc*^*−/−*^ mice exhibited higher fibrosis levels compared to age-matched wild-type mice as quantified by Masson’s Trichrome staining (Fig. 8A). To test the impact of tDDR inhibition in this setting, fibrotic G3 *Terc*^*−/−*^ mice were treated with intraperitoneal tASOs injections twice a week for four weeks, similarly to Fig. 2A, and sacrificed at 20 months of age (Fig. 8B). Also in this advanced fibrosis model, tDDR inhibition induced a substantial reduction in fibrosis as determined by Masson’s Trichrome staining and quantification (Fig. 8C) and a significant improvement of emphysema-like features (Fig. 8D).

These results indicate that tASOs treatment can be effective in reducing fibrosis and improving lung structure, also in old animals with a long-established pathologic phenotype.

### G3 *Terc*^*−/−*^ mice recapitulate human IPF transcriptomic changes

**Figure 9:**
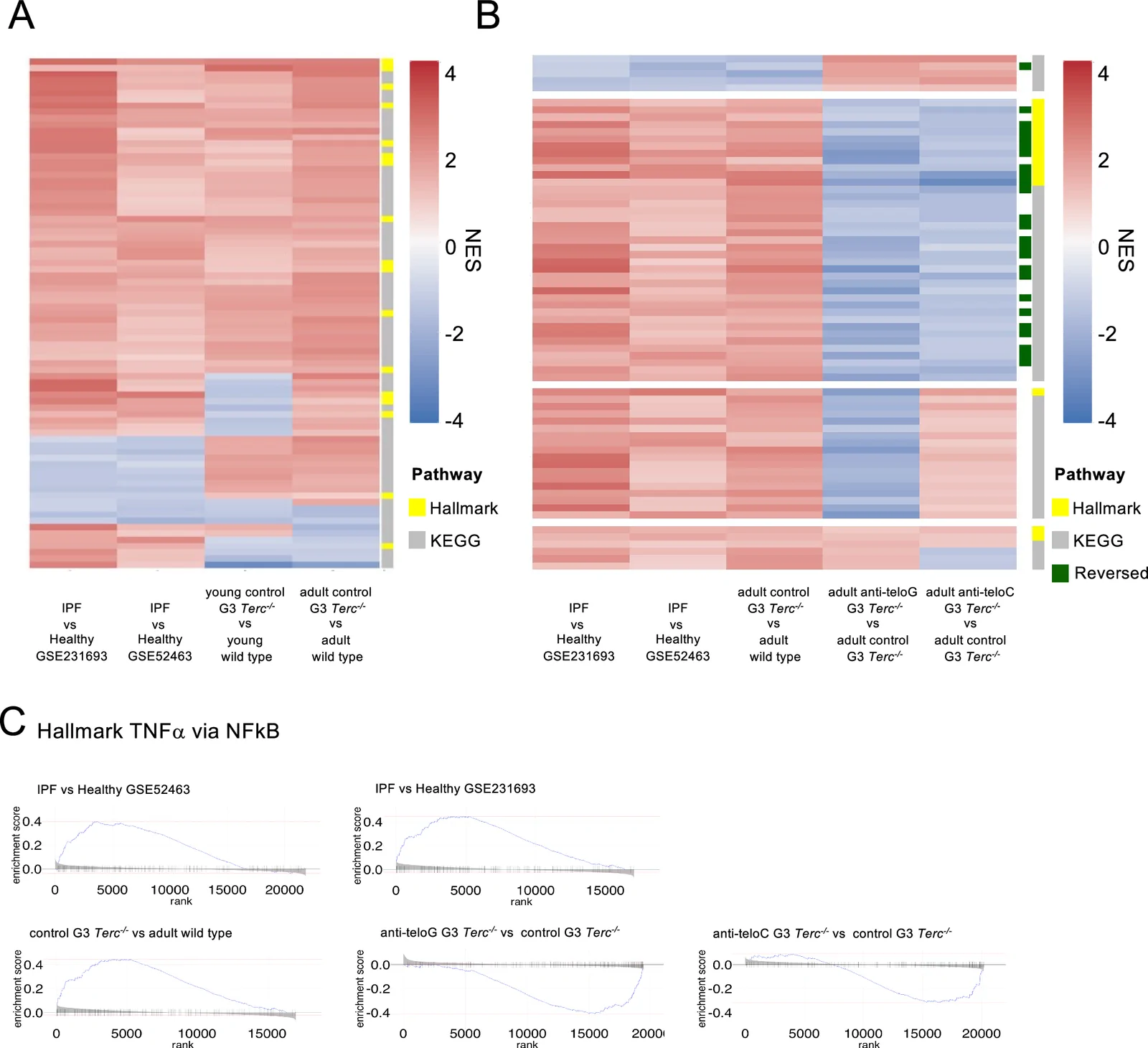
tDDR inhibition normalizes the pathways deregulated in human IPF. (**A**) Heatmap of the Normalized Enrichment Score for KEGG (gray) and Hallmark (yellow) pathways in human IPF patients vs healthy controls (data from GSE231693 (Data ref: Jia et al, 2023) and GSE52463 (Data ref: Nance et al, 2014) datasets), G3 *Terc*^*−/−*^ vs wild-type young mice, and control G3 *Terc*^*−/−*^ vs wild-type adult mice (young wild-type *n* = 4 mice, young G3 *Terc*^*−/−*^ *n* = 4 mice adult wild-type *n* = 5 mice, adult control G3 *Terc*^*−/−*^ *n* = 5 mice). The consistently modified human pathways among human studies were selected, and their behavior in control G3 *Terc*^*−/−*^ vs adult wild-type mouse are shown. (**B**) Heatmap of the Normalized Enrichment Score for KEGG (yellow) and Hallmark (gray) selected pathways as defined in Fig. 9A (adult wild-type *n* = 5 mice, control G3 *Terc*^*−/−*^ *n* = 5 mice, anti-teloG G3 *Terc*^*−/−*^ *n* = 5 mice, anti-teloC G3 *Terc*^*−/−*^ *n* = 5 mice). A hierarchical classification followed by a k-means clustering (*k* = 4) was performed for the indicated comparisons. The first two clusters list the pathways defined as reversed in Fig. EV4D,E (green). (**C**) GSEA enrichment plot for the TNFα via NF-κB pathway for the indicated comparisons.

**Figure EV9:**
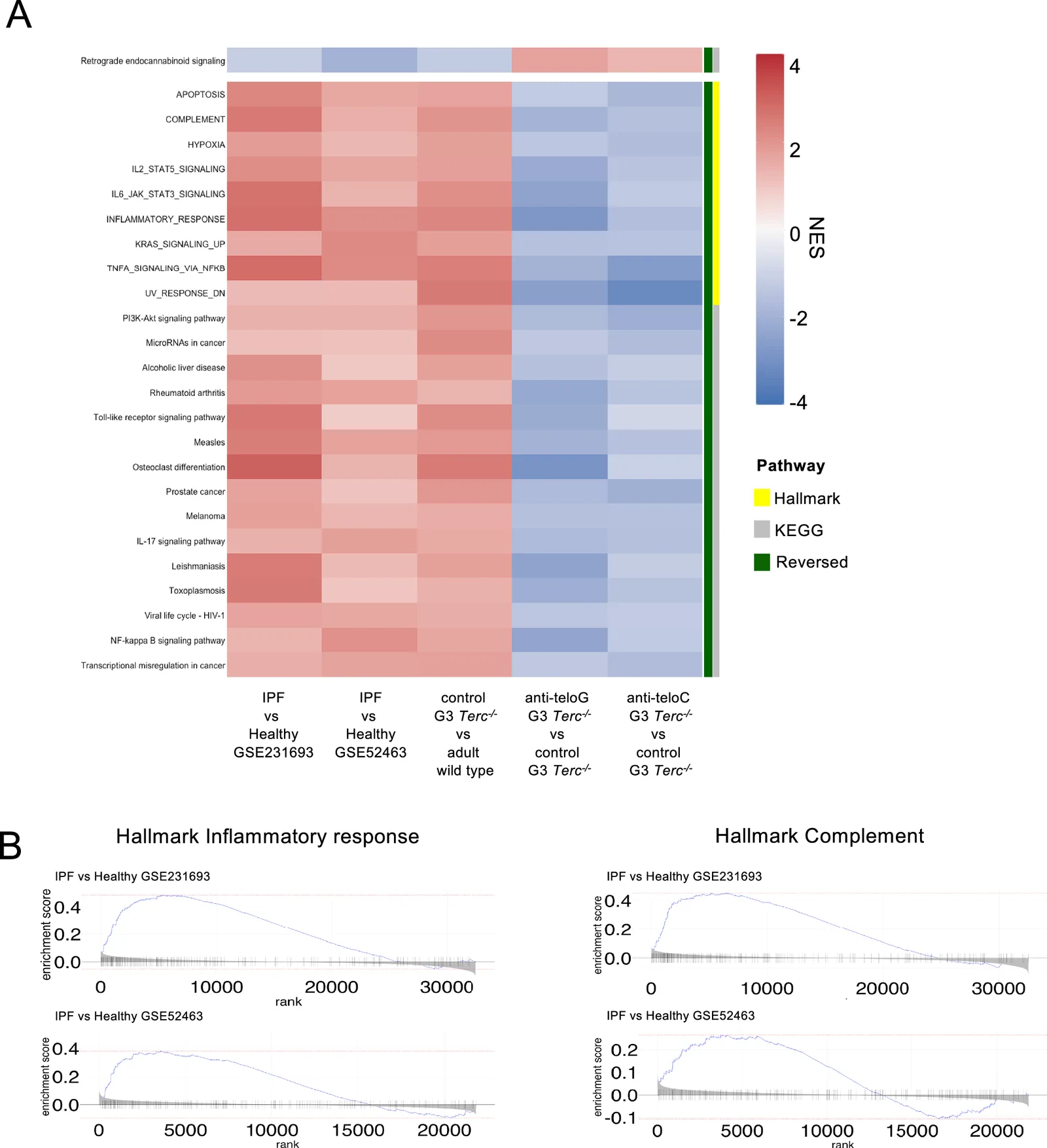
tDDR inhibition normalizes pathways in the lungs of adult G3 *Terc*^*−/−*^ mice, which are altered in human IPF patients, including inflammatory signaling. (**A**) Heatmap of the Normalized Enrichment Score for the reversed KEGG pathways (gray, lowercase) and Hallmark pathways (yellow, uppercase) in human and control G3 *Terc*^*−/−*^ mice for the indicated comparisons. (**B**) GSEA plot for the Inflammatory response pathway and Complement pathways for the indicated comparisons.

Finally, we assessed whether and to what extent the G3 *Terc*^*−/−*^ mouse model recapitulates the changes observed in the lungs of IPF patients. We therefore compared our mouse RNA-seq datasets from young and adult mice (Fig. 4) with two independent, publicly available datasets generated with lung samples of IPF patients and healthy controls (Data ref: Jia et al, 2023) (Data ref: Nance et al, 2014). To enhance analytical robustness and minimize human dataset variability, we focused on those Hallmark and KEGG pathways that were dysregulated in the same direction in both human cohorts. Differential pathway enrichment analyses revealed a strong similarity in changes observed in G3 *Terc*^*−/−*^ mouse lungs at both ages and human IPF samples (Fig. 9A). When we compared selected pathways concordantly altered in both species and analyzed the impact of tDDR inhibition by tASOs, we observed that most of them were reversed by one or both tASOs treatments (Fig. 9B). Moreover, the pathways that were reversed in mice (Fig. EV4B,C) presented a similar deregulated pattern in IPF patients (Fig. EV9A). Among the pathways upregulated in both human IPF datasets and control G3 *Terc*^*−/−*^ mice compared with their controls and downregulated upon tDDR inhibition, we noticed the TNFα via NF-κB signaling pathway (Fig. 9C), which is relevant for its known role in pro-inflammatory signaling and immune cell recruitment also in IPF (Guo et al, 2024; Alharbi et al, 2021). This observation aligns with and strengthens the observed increased lung fibrosis determined by histological evaluations in control G3 *Terc*^*−/−*^ mice and its reduction following tDDR inhibition (Fig. 3B). Similarly, the inflammatory and complement pathways, which are upregulated in G3 *Terc*^*−/−*^ mice and reduced upon tASOs treatment (Fig. 5E,F), were also consistently upregulated in the human IPF datasets (Fig. EV9B).

Overall, these results confirm the faithfulness of the telomerase-deficient mouse model employed in this study in recapitulating key molecular and pathological features of the human IPF condition and support its value as a translational model. tASOs treatments ameliorate disease manifestations in G3 *Terc*^*−/−*^ mice and restore multiple dysregulated pathways that are also altered in IPF patients, further reinforcing the therapeutic relevance of this approach.

## Discussion

Here, we adopted a genetic mouse model of IPF bearing the inactivation of telomerase activity, a defect frequently observed in familial and sporadic patients, to investigate the role of telomere dysfunction-induced tDDR activation in IPF pathogenesis. In accordance with previous reports (Chen et al, 2015; Piñeiro-Hermida et al, 2020), we observed that this model recapitulates many aspects of the pathological lung phenotypes associated with the human disease, including telomere shortening, DDR activation, cellular senescence, immune activation, and fibrosis. G3 *Terc*^*−/−*^ mice reproduce transcriptional changes of aged mouse lungs and, most importantly, of lungs from human IPF patients, supporting its value as a model of both accelerated mouse aging and of human lung IPF pathology.

As a tool to probe the causative contribution of tDDR activation, we used two different tASOs targeting tdincRNA induced by telomere damage to suppress tDDR selectively (Rossiello et al, 2017; Aguado et al, 2019). Using this approach, we demonstrated that tASOs treatment reduces the tDDR in vivo, in mouse lungs, and this inhibition is associated with alleviation of lung pathology when tASOs treatment is administered both in young (3-month-old) and adult (10-month-old) mice, suggesting that the therapeutic benefit is independent of the length of time of fibrotic buildup. The observation that tASOs treatments slow down fibrosis progression (compare control and tASOs-treated conditions in Figs. 3B, 8C and  EV2E), and noticeably reduce fibrosis to a level lower than the starting point (compare Fig. 1D with  3B and Figs. EV2E and  8A with  8C), suggests that tASOs in this model not only slow down fibrosis progression but can also revert it. Furthermore, since mice showed significant lung fibrosis and increased senescence signatures at the time of treatment (Figs. 1D, 8A, and EV5F), our results suggest disease-modifying efficacy in therapeutic settings.

Interestingly, the observed beneficial effects are long-lasting and appear to persist for months after treatment. This prolonged effect could be related to an improvement of stem cells’ function, the major drivers of the pathology (Alder et al, 2015; Povedano et al, 2015; Liu et al, 2019), as indicated by the observed reduction of DDR in ATII cells. This improvement could lead to a rescue of their regenerative capacity and a reduced accumulation of lung fibrosis over time. In addition, since accumulation of senescent cells is also the consequence of reduced clearance by an impaired immune system (Prata et al, 2018), and since telomerase knockout mice show impaired hematopoiesis and immunity (Samper et al, 2002; Allsopp et al, 2003), which is rescued by tDDR inhibition (Oppezzo et al, 2026), tASOs treatments have the potential to improve lung pathology also due to an improved immune system. These two, not mutually exclusive, mechanisms can contribute synergistically to a sustained beneficial effect of ameliorating tissue pathology.

In these settings, tASOs treatment causes a reduction of several DDR markers, including γH2AX, a marker that was less impacted upon acute telomere dysfunction reported earlier (Rossiello et al, 2017; Aguado et al, 2019). This can be the result of cell autonomous mechanisms, by which persistent inhibition of tDDR by repeated dosing of tASOs may be more impactful and reduce γH2AX levels, and of non-cell autonomous effects of improved immune cell functions (Oppezzo et al, 2026), promoting senescence cell clearance and thus reducing γH2AX-positive cells. The notion that tDDR inhibition in primary senescent cells prevents the spread of secondary senescence due to paracrine and juxtacrine signaling (Admasu et al, 2021), may further contribute to the reduction of the number of γH2AX-positive cells (Nelson et al, 2012; Acosta et al, 2013; Victorelli et al, 2019).

Our results indicating efficacy of tDDR inhibition without affecting telomere length demonstrate that telomere length or dysfunction per se are not responsible for triggering lung pathology; rather, this is the consequence of tDDR activation. By targeting mechanisms immediately downstream of telomere dysfunction, this approach is expected to be effective in settings of different telomere dysfunction, regardless of its genetic origin, and perhaps also of environmental compounding factors. Consistently, our treatment successfully normalized the transcriptomic changes shared between telomerase-deficient mice and IPF patients, and perhaps not limited to those with short telomeres and/or with mutations in genes involved in telomere maintenance. The effects of the two tASOs tested were largely overlapping, with few differences. Further studies will determine the extent of the efficacy of tASOs treatment upon stratification of patients in relation to the cause of the pathology. In this regard, while one potential limitation of this study may be that *Terc*^−/−^ mice carry a genetic defect that—although common among IPF patients—is not the only telomere biology defect causing IPF, our previous demonstration of in vivo efficacy of tASOs in reducing tDDR independently from the genetic cause (i.e., upon loss of telomere-binding protein TRF2 or mutation in LMNA gene (Rossiello et al, 2017; Aguado et al, 2019), suggests broad applicability. Additionally, fibrosis resolution and therapeutic efficacy were assessed by histological and molecular endpoints rather than by longitudinal in vivo imaging or lung function testing. G3 *Terc*^*−/−*^ mice develop early fibrotic changes, and disease progression is gradual and more protracted than in IPF patients, reflecting species‑specific differences. Nevertheless, similarly to human patients, these animals have a reduced lifespan compared with wild‑type controls (Giorgio et al, 2016).

Normal aging is associated with increased telomere dysfunction (López-Otín et al, 2022). Considering that G3 *Terc*^*−/−*^ mice recapitulate many of the transcriptional changes observed in old mice, the efficacy of tASOs treatment in this model supports the possibility that this strategy may also be of use in age-dependent dysfunctions not induced by genetic alterations.

In summary, we observed that, by inhibiting the tDDR, cellular senescence, immune activation, and inflammation are consequently reduced, favoring reduced fibrosis and lung pathology. These results highlight the crucial role of tDDR in initiating and driving the progression of lung fibrosis and suggest tASOs as a promising therapeutic agent for IPF and potentially other diseases caused by telomeric dysfunction, including age-related conditions.

## Methods

**Table:** Reagents and tools table

Reagent/resource	Reference or source	Identifier or catalog number
**Experimental models**		
C57BL/6J (*M. musculus*)	Charles River Laboratories	
*Terc*^*+/−*^ (*M. musculus*)	Jackson Laboratory	B6. Cg-Terctm1Rdp/J Stock No: 004132 | mTR^−/−^
**Antibodies**		
anti-gH2AX phospho S139	Abcam	AB11174
anti-53BP1	Novus	NB100-304
anti-CD45 antibody	Abcam	AB10558
anti-Prosurfactant Protein C	Millipore	AB3786
anti-TRF2	Abcam	AB13579
Phospho-SMAD2/SMAD3 (Ser465, Ser467, Ser423, Ser425) Polyclonal Antibody	Thermo Fisher Scientific	PA5-110155
Anti-p21 antibody [EPR18021]	Abcam	AB188224
Vimentin (D21H3) XP® Rabbit mAb #5741	Cell Signaling	5741S
Anti-Cytokeratin 8 antibody	Abcam	ab59400
COL1A1 (E8F4L) XP® Rabbit mAb #72026	Cell Signaling	72026S
Phospho-ATM (Ser1981) Monoclonal Antibody (10H11.E12)	eBioscience™	14-9046-82
Rabbit anti-Phospho KAP-1 (S824) Antibody Affinity Purified	Bethyl	A300-767A
Smooth Muscle Actin (SMA)	Biocare Medical	CM 001 C
Hop (E-1)	Santa Cruz Biotechnology	sc-398703
Anti-Ki67 antibody	Abcam	ab15580
Donkey anti-Rabbit IgG (H+L) Highly Cross-Adsorbed Secondary Antibody, HRP	Thermo Fisher Scientific	A16035
Anti-Mouse IgG (whole molecule)–Peroxidase antibody produced in rabbit	Sigma	A9044
SignalStain® Boost IHC Detection Reagent (AP, Rabbit) #18653	Cell Signaling	18653S
ImmPRESS®-AP Horse Anti-Rabbit IgG Polymer Detection Kit, Alkaline Phosphatase	Vector	MP-5401
Goat anti-Rabbit IgG (H+L) Cross-Adsorbed Secondary Antibody, Alexa Fluor™ 568	Invitrogen	A-11011
**Oligonucleotides and other sequence-based reagents**		
Control ASO	Qiagen	ACTGATAGGGAGTGGTAAACT
Anti-teloG ASO	Qiagen	CCCTAACCCTAACCCTAACCC
Anti-teloC ASO	Qiagen	GGGTTAGGGTTAGGGTTAGGG
Cy5-conjugated TelC PNA probe	Panagene	F1003
Actin forward	Sigma-Aldrich	CTAAGGCCAACCGTGAAAAG
Actin reverse	Sigma-Aldrich	ACCAGAGGCATACAGGGACA
Telo forward	Sigma-Aldrich	CGGTTTGTTTGGGTTTGGGTTTGGGTTTGGGTTTGGGTT
Telo reverse	Sigma-Aldrich	GGCTTGCCTTACCCTTACCCTTACCCTTACCCTTACCCT
RT_C-Rich Rv	Sigma-Aldrich	CCCTAACCCTAACCCTAA
RT_G-Rich Rv	Sigma-Aldrich	TAGGGTTAGGGTTAGGG
Rplp0 Fw	Sigma-Aldrich	TTCATTGTGGGAGCAGAC
Rplp0 Rv	Sigma-Aldrich	CAGCAGTTTCTCCAGAGC
DNA probe C-rich StY11 probe	https://pubmed.ncbi.nlm.nih.gov/35396546/	
**Chemicals, enzymes, and other reagents**		
Proteinase K, recombinant, PCR grade	Thermo Fisher Scientific	E00492
RNase A	Qiagen	19101
MboI	New England Biolabs	R0147L
SSC buffer (20X)	Thermo Fisher Scientific	J60839.K2
PerfectHyb plus Hybridization buffer	Sigma-Aldrich	H7033
VECTASHIELD® Antifade Mounting Medium	Vector Laboratories	H1000-10
RNase-Free DNase Set DNase I	Qiagen	79256
TRIzol	Invitrogen	15596026
LightCycler 480 SYBR Green I Master	Roche	4707516001
TURBO DNase	Thermo Fisher Scientific	AM2238
SuperScript™ III Reverse Transcriptase	Thermo Fisher Scientific	18080044
Microspin™ G-50 Columns	Merck	GE27-5330-01
Hydroxyproline Assay Kit (Perchlorate-Free)	Merk	MAK357
DNeasy Blood & Tissue Kit	Qiagen	69506
RNeasy Plus Universal Mini Kit	Qiagen	73404
EvaGreen Supermix	Bio-Rad	1725200
SuperScript VILO cDNA Synthesis Kit	Thermo Fisher Scientific	11754050
Stranded mRNA prep ligation kit	Illumina	20040532
NaveniBright HRP kit	Navinci Diagnostics	60030
Maxwell® RSC simplyRNA Tissue Kit	Promega	AS134
RNA-later	Thermo Fisher Scientific	AM7024
RNAseOut™ Recombinant Ribonuclease Inhibitor	Thermo Fisher Scientific	100000840
10% neutral buffered formalin	Diapath	F0043
Eosin Y 1% aqueous solution	Bio-Optica	05-10007/L
Harris’ Hematoxylin	Bio-Optica	05-06004
Masson’s trichrome Stain kit	Diapath	010210
X-free Solvent for histology xylene substitute	Bio-Optica	06-1305 F
Epitope Retrieval Solution pH 6 (x10 Concentrate)	Leica Biosystems	RE7113-CE
Epitope Retrieval Solution pH 9 (x10 Concentrate)	Leica Biosystems	RE7119-CE
A – PapPen for immunohistochemistry	Bio-Optica	11-100
Novolink Polymer Detection Systems	Leica Biosystems	RE7280-CE / RE7280-K
Antibody Diluent	Leica Biosystems	RE7133-CE
DAB CHROMOGEN SYSTEM PACK 100 ML	BIOCARE MEDICAL	DB801L
Romulin AEC Chromogen Kit	BIOCARE MEDICAL	RAEC810
Vulcan Fast Red Chromogen Kit 2	BIOCARE MEDICAL	FR805M
Gill’s Hematoxylin n.1	Bio-Optica	05-06013/L
Micromount	Leica Biosystems	3801731
Fluorescence Mounting Medium, Mounting medium, 15 mL	Agilent	S3023
**Software**		
Halo Software	v.3.5.3577.140, Indica Labs	
Aperio ImageScope	v.12.3.2.8013, Leica Biosystems	
Pannoramic Scanner Software	4.1.0, 3DHISTECH	
Zen Software	v.2.0 and v.3.1, Zeiss	
GraphPad Prism	v.10.0	
Cell Profiler	v4.2.6	
R	v.4.4.0	
Fiji/ImageJ	V2.9	
**Other**		
Leica SP5 confocal microscope	Leica	
Maxwell® RSC Instrument	Promega	
NanoVueTM Plus Spectrophotometer	GE Healthcare	
LightCycler 96 Roche	Roche	
nf-core rnaseq pipeline		v3.12.0-g3bec233
Platform		x86_64-apple-darwin20
ChemiDoc MP Imaging System	Bio-Rad	
Qubit Fluorimeter	Thermo Fisher Scientific	
Agilent Bioanalyzer 2100 instrument	Agilent Technologies	
Illumina NextSeq550Dx sequencer	Illumina	
Tissue Lyser	Qiagen	
Aperio CS2 digital scanner	Leica Biosystems	
Pannoramic 250 Flash III DX Digital Scanner	3DHISTECH	
Zeiss Axioscope A1	Zeiss	
Zeiss Axioscope 5	Zeiss	
Digital water bath 5 L WB-MD5	FALC	

### Animals and treatments

Experiments involving animals have been carried out in accordance with the Italian Law (D.lgs. 26/2014), which enforces European Directive 2010/63/EU on the protection of animals used for scientific purposes. Accordingly, the study protocol was authorized by the Italian Ministry of Health, the national Competent Authority (project number 565/2021).

Mice were housed in individually ventilated cages (IVCs) under specific pathogen-free (SPF) conditions.

C57BL/6J mice were purchased from Charles River Laboratories. *Terc*^*+/−*^ mice (B6. Cg-Terctm1Rdp/J Stock No: 004132 | mTR^−/−^) (Blasco et al, 1997) were purchased by Jackson Laboratory. *Terc*^*+/−*^ mice were intercrossed to generate first-generation (G1) homozygous *Terc*^*−/−*^ knockout mice. Second-generation (G2) *Terc*^*−/−*^ mice were generated by successive breeding of G1 *Terc*^*−/−*^ and then G3 *Terc*^*−/−*^ mice by crosses between G2 *Terc*^*−/−*^ mice. For this study, we used male mice (for the analyses in Fig. 1 and in the experimental setting described in Figs. 2A and EV2A) or female mice (in the experimental setting described in Fig. 8B).

Mice were injected intraperitoneally (i.p.) at 2 or 10 months of age with a control, anti-teloG, or anti-teloC ASO at a final concentration of 15 mg/kg twice a week for 4 weeks and sacrificed at 5, 12, or 20 months of age. Group sizes were calculated to achieve sufficient statistical power. Animals were allocated to treatment groups in a cage‑balanced manner, with treatments distributed across different cages to minimize cage effects and subjective allocation bias. No blinding was performed during treatment administration or outcome assessment. Lungs were collected and a part of each of them was snap-frozen and stored in RNA-later (Thermo Fisher Scientific, AM7024) for RNA extraction and another was washed in PBS and collected for fixation in 10% neutral-buffered formalin (Diapath S.p.A., #F0043) at 4 °C overnight, washed in running water at slow flux for 5 h, transferred to 70% ethanol, dehydrated, and embedded in paraffin for histological analysis. Paraffin-embedded tissues were cut into 4-μm sections for analyses.

### Antisense oligonucleotides (ASOs) sequences

The locked nucleic acid-modified oligonucleotides with a fully phosphorothioate backbone were produced by Qiagen as described (Rossiello et al, 2017).

Sequences were as follows (5′–3′ orientation):

control: ACTGATAGGGAGTGGTAAACT

anti-teloG: CCCTAACCCTAACCCTAACCC

anti-teloC: GGGTTAGGGTTAGGGTTAGGG.

### Histopathological examination and air spaces analysis

Paraffin-embedded lung sections were routinely stained with hematoxylin and eosin (H&E) for histopathological analysis. Whole-slide images of stained sections were acquired using a Pannoramic 250 Flash III DX Digital Scanner (3DHISTECH) with Pannoramic Scanner Software 4.1.0 (3DHISTECH) to visualize the entire lung section. For the histopathological evaluation, a semiquantitative scoring system was utilized, which involved the following factors: inter-alveolar septal thickening, interstitial cellularity increase, interstitial inflammatory infiltration, intra-alveolar inflammatory infiltration, and erythrocyte extravasation. All the factors were morphologically evaluated blind and scored according to the degree of severity (0, normal; 1, slight; 2, moderate; 3, marked) and extension (0, absent; 1, focal; 2, multifocal; 3, diffuse). An overall morphological and pathological score was calculated for each sample as the product of the degree of severity and the extension.

Lung fibrosis was additionally assessed using the modified Ashcroft scoring system as described by (Hübner et al, 2008). Semiquantitative evaluation was performed on the experimental groups. Ten non-overlapping microphotographs per sample, acquired at x200 magnification, were analyzed, and fibrosis severity was graded according to the reference scale. The mean score per animal was calculated to represent the extent of pulmonary fibrosis.

To determine the number of air spaces, slide digitalization was performed using an Aperio CS2 digital scanner (Leica Biosystems) with the ImageScope software (Aperio ImageScope version 12.3.2.8013, Leica Biosystems), and a semiquantitative analysis was conducted on five nonoverlapping fields per sample, using a medium-power magnification of ×200.

### Masson’s Trichrome staining

Masson’s Trichrome staining was performed on paraffin-embedded lung sections with Masson’s Trichrome Stain kit (DiaPath) following the manufacturer’s protocol. This staining method highlights collagen fibers in blue and muscular fibers in red. Slide digitalization was performed using an Aperio CS2 digital scanner (Leica Biosystems) with the ImageScope software (Aperio ImageScope version 12.3.2.8013, Leica Biosystems) and a Pannoramic 250 Flash III DX Digital Scanner (3DHISTECH) with Pannoramic Scanner Software 4.1.0 (3DHISTECH) to visualize the entire lung section. To quantify the lung fibrosis, the “Area Quantification v2.4.2” algorithm of HALO software (Indica Labs) was utilized specifically for quantifying the blue-colored collagen fibers. This algorithm enabled the evaluation of Masson’s Trichrome staining in five separate fields, without overlap, using a medium-power magnification ×200 and independently of cell segmentation.

### Immunolocalization and quantitative analyses

Paraffin-embedded lung sections were deparaffinized, rehydrated, and unmasked using Novocastra Epitope Retrieval Solutions pH 6 or pH 9 (Leica Biosystems) in a thermostatic bath at 98 °C for 30 min. Subsequently, the sections were brought to room temperature and washed in PBS. After neutralization of the endogenous peroxidase with 3% H_2_O_2_ and Fc blocking with 0.4% casein in PBS (Leica Biosystems), the samples were incubated with the primary antibodies. Immunohistochemistry (IHC) was developed using anti-Rabbit IgG (H + L) Highly Cross-Adsorbed Secondary Antibody HRP (Invitrogen, 1:500) or Anti-Mouse IgG (whole molecule)–Peroxidase antibody produced in rabbit (Sigma, 1:200) and DAB (3,30-Diaminobenzidine, Biocare Medical) or Romulin AEC Chromogen Kit (Biocare Medical) as substrate chromogen. Double IHC staining was performed by applying SignalStainBoost IHC Detection rabbit (Cell Signaling Technology) alkaline phosphatase-conjugated or ImmPRESS®-AP Horse Anti-Rabbit IgG Polymer Detection Kit, Alkaline Phosphatase (Vector), and Vulcan Fast Red (Biocare Medical) as substrate chromogen. Nuclei were counter-stained with Gill’s hematoxylin No. 1 (BioOptica). For immunofluorescence (IF), the binding of the primary antibody to the antigenic substrate was revealed by Goat anti-Rabbit IgG (H + L) Cross-Adsorbed Secondary Antibody, Alexa Fluor™ 568 (Life Technologies, 1:300), and nuclei were subsequently visualized with DAPI.

Slides were analyzed under a Zeiss Axioscope A1 and Zeiss Axioscope 5 microscopes equipped with four fluorescence channels, widefield IF. Microphotographs were collected using a Zeiss Axiocam 503 and Axiocam 807 Color digital camera with the Zen 2.0 and Zen 3.1 Software (Zeiss). Quantitative analyses of γH2AX, 53BP1, pATM, pKAP1, aSMA, COL1A1, pSMAD2/3, p21, CD45, HOPX, KRT8, Ki-67 IHC mouse stainings were performed by calculating the average percentage of positive signals in five nonoverlapping fields at medium-power magnification x200 using the Nuclear v9 or Positive Pixel Count v9 Aperio ImageScope software and “Multiplex IHC v3.2.3” or Area Quantification v2.4.2” algorithms of HALO software (Indica Labs). IF staining (pro-SPC) was analyzed through the use of the segmentation-based algorithm “HighPlex FL v4.2.3” of HALO software (Indica Labs), quantifying the average percentage of positive cells in five nonoverlapping fields at medium-power magnification x200. Double IHC (γH2AX and pro-SPC; 53BP1 and pro-SPC, γH2AX and VIMENTIN) stainings were analyzed through the use of the segmentation-based algorithm “Multiplex IHC v3.2.3” of HALO software (Indica Labs), quantifying the average percentage of γH2AX+ or 53BP1+ cells in type II pneumocytic pro-SP-C+ cells and γH2AX+ cells in mesenchymal VIMENTIN-positive cells in five nonoverlapping fields at medium-power magnification ×200.

### Fluorescence in situ hybridization on mouse tissues

Mouse lungs were fixed with 4% paraformaldehyde (PFA) for 1 h and cryoprotected with Sucrose 30% before freezing. Seven-mm tissue sections were permeabilized with triton 0.1% for 20 min RT, incubated with glycine 10 mM for 30 min RT, washed, denatured at 80 °C for 5 min, and incubated with Cy5-conjugated TelC PNA probe (F1003, Panagene). After washing, nuclei were stained with 4,6-diamidino-2-phenylindole (DAPI; 1 mg/ml). Slides were mounted with VECTASHIELD® Antifade Mounting Medium (H-1000-10). Image acquisition was performed with a Leica TCS SP5 confocal microscope. The detection parameters were set in the control samples and were kept constant across specimens. The quantification of the signal was performed in a semi-quantitative manner by CellProfiler software.

### Proximity ligation assay

Proximity ligation assay (PLA) was performed using NaveniBright HRP kit (Navinci Diagnostics) according to the manufacturer’s protocol using the following primary antibodies: rabbit polyclonal anti-γH2AX phospho S139 (1:1000 pH 6, ab11174, Abcam) and mouse monoclonal TRF2 (clone 4A794, 1:200 pH 9, ab13579, Abcam). Negative controls were performed using only one primary antibody. Slides were analyzed under a Zeiss Axioscope A1, and microphotographs were collected using a Zeiss Axiocam 503 Color with the Zen 2.0 Software (Zeiss). Segmented images were obtained using HALO image analysis software (v3.2.1851.229, Indica Labs). HALO image analysis software was used to quantify the PLA signals in ten non-overlapping fields at high-power magnification (×400), and the output was expressed as the average of signal area per cell (in μm^2^).

### Antibodies

The following primary antibodies were used for IHC and IF: rabbit polyclonal anti-γH2AX phospho S139 (1:1000, pH 6, AB11174, Abcam), rabbit polyclonal anti-53BP1 (1:1000, pH 6, NB100-304, Novus), mouse monoclonal anti-Phospho-ATM (Ser1981) (1:100, pH 6, 14-9046-82, Invitrogen), rabbit polyclonal anti-Phospho KAP-1 (S824) (1:200, pH 9, A300-767A, Bethyl), mouse monoclonal anti-Smooth Muscle Actin (SMA) (1:500, pH 9, CM 001 C, Biocare Medical), rabbit monoclonal anti-COL1A1 (1:100, pH 9, 72026S, Cell Signaling), rabbit polyclonal anti-Phospho-SMAD2/SMAD3 (Ser465, Ser467, Ser423, Ser425) (1:1000, pH 6, PA5-110155, Invitrogen), rabbit monoclonal anti-p21 (1:500, pH 6, ab188224, Abcam), rabbit polyclonal anti-CD45 antibody (1:500, pH 9, AB10558, Abcam), rabbit polyclonal anti-Prosurfactant Protein C (pro-SPC) (1:200, pH 9, AB3786, Millipore), mouse monoclonal anti-HOPX (1:100, pH 6, sc-398703, Santa Cruz Biotechnology), rabbit polyclonal anti-Cytokeratin 8 (1:500, pH 9, ab59400, Abcam), rabbit polyclonal anti-Ki-67 (1:1000, pH 6, ab15580, Abcam), rabbit monoclonal anti-Vimentin (1:200, pH 9, 5741S, Cell Signaling).

### Telomere restriction fragment analysis

Lung tissue was homogenized in TNE buffer (100 mM Tris–HCl pH 8.0, 10 mM EDTA pH 8.0, 100 mM NaCl) and lysed in TNES (TNE 1×, 1% SDS (wt/vol)) supplemented with RNase A, followed by proteinase K digestion at 37 °C. Genomic DNA was purified by sequential phenol–chloroform–isoamyl alcohol and chloroform extractions, precipitated with sodium acetate and isopropanol, washed with 70% ethanol, and gently resuspended in TrisHCl 10 mM pH 8.0 buffer. DNA concentration was determined by fluorimetry (Qubit Broad Range). For restriction digestion, 5 μg of genomic DNA was incubated in CutSmart buffer to ensure homogeneous resuspension, followed by overnight digestion with MboI at 37 °C, with additional enzyme supplementation the next day. Digested DNA was recovered by isopropanol precipitation, washed with ethanol, and resuspended in TrisHCl 10 mM pH 8.0 buffer. The digested DNA fragments were separated by gel pulsed-field gel electrophoresis (PFGE). DNA samples were resolved on agarose gels under alternating electric fields with optimized pulse times and field strength, stained with ethidium bromide, and visualized by UV illumination. Next, the gel was incubated in depurination buffer (0.25 M HCl) for 30 min under gentle orbital shaking and then incubated in denaturing solution twice 30 min under gentle orbital shaking and subsequently neutralized using neutralization solution for an additional 30 min. The denatured DNA fragments were transferred onto a nylon membrane by capillary blotting using 20× SSC buffer overnight. After transfer, the DNA was immobilized on the membrane by UV cross-linking using a Stratalinker with 254 nm UV bulbs and the “autocross-link” function. The membrane was pre-hybridized in hybridization buffer at the appropriate temperature for 1–2 h to prevent non-specific binding. Hybridization was then carried out by incubating the membrane with a labeled DNA probe, C-rich StY11 probe (prepared as described (Mazzucco et al, 2022) at the same temperature overnight. Following hybridization, the membrane was washed with a series of low- and high-stringency wash buffers to remove unbound probe. Detection of hybridized probe was performed using autoradiography or chemiluminescent detection, depending on the labeling method. The resulting bands were visualized and analyzed to determine the presence and size of the target DNA fragments by Amersham™ Typhoon™ biomolecular imagers. Quantification of the radioactive signal was performed using Fiji (ImageJ). Normalization of lane-to-lane variation was achieved using Ethidium bromide–stained total DNA. Telomere fragment sizes were estimated by interpolation from the migration profile of the DNA ladder, and the median fragment length was calculated accordingly.

### Quantitative PCR for telomere length

DNA was extracted by DNeasy Blood and Tissue Kit (Qiagen 69506), according to the manufacturer’s instructions. Relative telomere length was measured by qPCR using SsoFast™ EvaGreen® Supermix (Bio-Rad, #1725200), adapting a previously published protocol (Vasilishina et al, 2019). In total, 20 ng of DNA were used in each reaction, and the following primers were used:

Actin Fw: 5’-CTAAGGCCAACCGTGAAAAG-3’

Actin Rv: 5’- ACCAGAGGCATACAGGGACA-3’

Telo Fw: 5’-CGGTTTGTTTGGGTTTGGGTTTGGGTTTGGGTTTGGGTT-3’

Telo Rv: 5’- GGCTTGCCTTACCCTTACCCTTACCCTTACCCTTACCCT-3’.

qPCR was performed on a LightCycler® 96 Instrument, using the following thermal cycling conditions: an initial denaturation step at 98 °C for 120 s, followed by 45 cycles of denaturation at 98 °C for 5 s, annealing, and extension at 60 °C for 20 s.

### Strand-specific RT-qPCR for tdincRNA detection

For tdincRNA, lung tissue was stored in RNA-later (Thermo Fisher Scientific, AM7024) until use. The samples were then resuspended in Homogenization solution (Promega, AS134) and 1-Thioglycerol (Promega, AS134), and lysed with TissueLyser II (Qiagen). Proteinase K (Thermo Fisher Scientific, E00492) and RNAseOut™ Recombinant Ribonuclease Inhibitor (Thermo Fisher Scientific, 100000840) were then added, and samples were incubated at 56 °C for 10 min. RNA extraction was then performed using Maxwell® RSC Instrument (Promega), with Maxwell® RSC simplyRNA Tissue Kit (Promega, AS134), following the manufacturer’s instructions. Detection of tdincRNAs was performed as previously described (Rossiello et al, 2017), with some modifications. Briefly, 1 μg of total RNA samples were treated with TURBO DNase (Thermo Fisher Scientific, AM2238) at 37 °C for 1 h. Next, RNA was reverse transcribed using the SuperScript III Reverse Transcriptase kit (Thermo Fisher Scientific, 18080044) with strand-specific primers. cDNA was passed on a Microspin™ G-50 Columns (Merck, GE27-5330-01) and qPCR was performed using LightCycler 480 SYBR Green I Master (Roche, 04707516001) and run on LightCycler 96 Roche detection system. A volume of cDNA corresponding to 20 ng of initial RNA was used. Each reaction was performed in triplicate. Rplp0 was used as a control gene for normalization.

Primer sequences (5′–3′ orientation) were:

RT_C-Rich Rv CCCTAACCCTAACCCTAA

RT_G-Rich Rv TAGGGTTAGGGTTAGGG

Rplp0 Fw TTCATTGTGGGAGCAGAC

Rplp0 Rv CAGCAGTTTCTCCAGAGC

telo Fw CGGTTTGTTTGGGTTTGGGTTTGGGTTTGGGTTTGGGTT

telo Rv GGCTTGCCTTACCCTTACCCTTACCC TTACCCTTACCCT.

### Illumina stranded mRNA sequencing

To isolate RNA, 20-30 mg lung tissue was homogenized in TRIzol (Invitrogen, 15596026) with TissueLyser II (Qiagen) and processed with RNeasy Plus Universal Mini Kit (Qiagen, 73404) according to the manufacturer’s specifications. To increase the purity of the RNA extracted, an on-column RNase-Free DNase Set DNase I (Qiagen, 79256) treatment was performed to remove the residual amounts of DNA. The NanoDrop spectrophotometer was used to detect RNA quantity and purity. RNA purity was ascertained via NanoDrop 260/280 and 260/230 ratios. The abundance and integrity of total RNA were assessed using a Qubit fluorimeter and an Agilent Bioanalyzer 2100 instrument (Agilent Technologies, Palo Alto, CA). For each sample, 500 ng of total RNA were used to generate a library of fragments using the Stranded mRNA prep ligation kit (Illumina, 20040532). Oligo(dT) magnetic beads purify and capture the mRNA molecules containing polyA tails. The purified mRNA was fragmented and copied into first-strand complementary DNA (cDNA) using reverse transcriptase and random primers. In a second strand cDNA synthesis step, dUTP replaces dTTP to achieve strand specificity. The final steps added adenine (A) and thymine (T) bases to fragment ends and ligate adapters. The resulting products were purified and selectively amplified with 12 cycles of PCR to generate an indexed library of fragments. The library was quantified using a Qubit 4.0 fluorimeter, checked on an Agilent Bioanalyzer 2100, and then loaded on an Illumina NextSeq550Dx sequencer for sequencing, following the manufacturer’s instructions.

Library fragments were sequenced using 2 × 75 nt read mode, thus sequencing 75 nucleotides from both ends of each fragment; on average, ~50 Million Paired-end fragments were sequenced for each sample. Sequencing results were generated in fastq.gz format.

### RNA-seq preprocessing and pathway analysis for mouse lungs

Sequencing reads were aligned and counted with the NF-core/rnaseq pipeline with default options (Patel et al, 2025; Ewels et al, 2020) (v3.12.0-g3bec233). The reference genome on mm10 was used (Church et al, 2011). The quality check was performed with FastQC (Andrews, 2010) and SampleNetwork (Oldham et al, 2012). The differential analysis was performed on R (version 4.4.0) (Team 2024) with DESeq2 (Love et al, 2014). Differentially expressed genes were defined as genes with *P* value adjusted (Benjamini–Hochberg) < 0.05. The Pathway Analysis was carried out with fsgea (Korotkevich et al, 2021) and the clusterProfiler package (Xu et al, 2024; Wu et al, 2021), independently for all comparisons, and aggregated into a single matrix of Normalized Enriched Score (NES) and *P* value adjusted to perform the clustering analyses. The Hallmark pathways and Sen Mayo Signature are from the Mouse MSigDB Collection (Mootha et al, 2003; Subramanian et al, 2005; Liberzon et al, 2015), the Universal Signature was defined by (Hernandez-Segura et al, 2017b). The Mice KEGG pathways are from clusterProfiler (Xu et al, 2024; Wu et al, 2021). The pathways for TGFβ, lung fibrosis, and myofibroblast differentiation are from (Panagiotidis et al, 2025; Lingampally et al, 2025; El Agha et al, 2017). Gene signatures from ATII, ATI, and KRT8-positive cells are from (Strunz et al, 2020). For the pathway enrichment analysis, genes were ranked in descending order of statistics score. The GSEA plots were done with fsgea (Korotkevich et al, 2021) and the ggplot package. The heatmap and the k-means clustering were performed with the ComplexHeatmap package (Gu, 2022). First, we built the heatmap with the NES for all the pathways (KEGG and Hallmark), then we performed a hierarchical and a k-means clustering on this heatmap (*k* = 8). We define as reversed, the pathways that had NES > 0 in old wild-type vs adult wild-type and control G3 *Terc*^*−/−*^ vs adult wild-type and NES < 0 in anti-teloC G3 *Terc*^*−/−*^ vs control G3 *Terc*^*−/−*^ and anti-teloG G3 *Terc*^*−/−*^ vs control G3 *Terc*^*−/−*^ or NES < 0 in old wild-type vs adult wild-type and control G3 *Terc*^*−/−*^ vs adult wild-type and NES > 0 in anti-teloC G3 *Terc*^*−/−*^ vs control G3 *Terc*^*−/−*^ and anti-teloG G3 *Terc*^*−/−*^ vs control G3 *Terc*^*−/−*^ and are consistent. We retained the reversed pathways with *P* value adjusted on control G3 *Terc*^*−/−*^ vs adult wild-type significant (*P* value adjusted <0.05). The distance matrix was calculated for the NES matrix of the reversed pathways for the four comparisons (old wild-type vs adult wild-type; control G3 *Terc*^*−/−*^ vs adult wild-type; anti-teloG G3 *Terc*^*−/−*^ vs control G3 *Terc*^*−/−*^; anti-teloC G3 *Terc*^*−/−*^ vs control G3 *Terc*^*−/−*^) using the function dist of stats package. Then the hierarchical clustering was performed with hclust function of stats package (Team 2024). The visualization of the dendrogram was done using ggdendrogram of the ggdendro package (de Vries and Ripley, 2024). We counted how many pathways were significantly deregulated (*P* value adjusted <0.05) and divided it by the total of pathways (*n* = 223 for KEGG; *n* = 50 for Hallmark). Then for the ones which were significantly deregulated into control G3 *Terc*^*−/−*^ vs adult wild-type, we counted how many have NES > 0 in control G3 *Terc*^*−/−*^ vs adult wild-type and NES < 0 under one or both treatments, but also counted how many have NES < 0 in control G3 *Terc*^*−/−*^ vs adult wild-type and NES > 0 under one or both treatments. Concerning the senescence, the Sen Mayo and Universal Senescence pathways were added to the Hallmark pathways, and the pathways analysis was performed with fsgea (Korotkevich et al, 2021). The NES of Sen Mayo and Universal Senescence signatures were printed using the ComplexHeatmap package (Gu, 2022). Same strategies were used for lung fibrosis, myofibroblast differentiation, and pulmonary cell lines lineage.

### Differential gene expression analysis

After selecting the 75% most variable genes from the log2-transformed dataset, we applied pyDeseq2 (version 0.4.11) to analyze the differential expression between the conditions. This technique uses a negative binomial model to extract log2-fold changes and *P* values from the raw gene expression. Specifically, we decided to refit outliers, identified through Cook’s distance, and to filter out genes with low counts across all conditions. Finally, two different heatmaps were produced to summarize the analysis. Significant genes for a specific benchmark group were selected by setting a maximum threshold for the adjusted *P* value at 0.05. The differential expression of these genes was then compared across other groups, showing the result of this comparison in a heatmap designed with Seaborn (version 0.13.2). Specifically, we first chose the old wild-type vs adult wild-type as a benchmark and compared it with the control G3 *Terc*^*−/−*^ vs adult wild-type. Then, we selected the control G3 *Terc*^*−/−*^ vs adult wild-type and compared its differential gene expression with old wild-type vs adult wild-type, anti-teloG G3 *Terc*^*−/−*^ vs control G3 *Terc*^*−/−*^, and anti-teloC G3 *Terc*^*−/−*^ vs control G3 *Terc*^*−/−*^.

### RNA-seq analysis and pathways analysis in human samples

The count matrix of published RNA-seq studies on IPF patients versus healthy controls (GSE231693 (Data ref: Jia et al, 2023) GSE52463 (Data ref: Nance et al, 2014)) were downloaded. A DESeq2 (Love et al, 2014) analysis for each study was conducted on R (version 4.4.0) (Team 2024) independently. After the usual replicate quality check and removal using a MDS approach using the Glimma package (Su et al, 2017), pathway analysis was performed for each study using clusterProfiler (Xu et al, 2024; Wu et al, 2021) for KEGG human pathways and fgsea (Korotkevich et al, 2021) for human Hallmark pathways. The KEGG and Hallmark pathway analyses were processed separately. The Human KEGG pathways are from clusterProfiler (Xu et al, 2024; Wu et al, 2021) and Human Hallmark from MSigDB Collection (Liberzon et al, 2015). For the pathway analysis enrichment analysis, genes were ranked in descending order of statistics score. Then, consistent pathways were selected. A consistent pathway is defined as having the same pattern in the two human studies. The Normalized Enrichment Scores (NES) and adjusted *P* values of human consistent pathways were combined with those from mouse studies into a comprehensive matrix. A heatmap of NES was assembled using ComplexHeatmap (Gu, 2022) for the two human comparisons and the control G3 *Terc*^*−/−*^ vs adult wild-type mice comparison. We selected the pathways with similar patterns in the three conditions, and we performed a k-means clustering (*k* = 4) for the five comparisons (the two human comparisons IPF vs Healthy and the 3 mice comparisons control G3 *Terc*^*−/−*^ vs adult wild-type; anti-teloG G3 *Terc*^*−/−*^ vs control G3 *Terc*^*−/−*^; anti-teloC G3 *Terc*^*−/−*^ vs control G3 *Terc*^*−/−*^) using ComplexHeatmap (Gu, 2022). The GSEA plots were done with the fsgea package (Korotkevich et al, 2021).

### Deconvolution analysis

Cell-type deconvolution was performed using three independent algorithms—CIBERSORTx (Newman et al, 2019), ImmuCC (Chen et al, 2017), and xCell (Aran et al, 2017)—to estimate the relative contributions of major immune and lung-associated cell populations across experimental conditions. For CIBERSORTx, we used the Tabula Muris single-cell RNA-seq lung dataset as the reference matrix. Deconvolution scores were computed for each sample and subsequently averaged within groups to assess global trends in cell-type abundance.

To investigate the relationship between cell-type–specific signatures and differential gene expression patterns, we performed a rank-based Gene Set Enrichment Analysis (GSEA) through the clusterProfiler R package (Xu et al, 2024). For each of the four pairwise comparisons—old wild-type vs. adult wild-type, control vs. adult wild-type, anti-teloG vs. control, and anti-teloC vs. control—genes were ranked according to their log₂ fold change. Cell-type marker gene sets were obtained from PanglaoDB, selecting canonical mouse markers associated with immune and lung-resident cell populations.

### Multivariable analysis, classification, and machine learning

The analysis was carried out on adult (12-month-old) and young (3-month-old) mice; missing entries were imputed using the mean value for the variable using Scikit-Learn (version 1.2.2). We then applied agglomerative hierarchical clustering to underline the relationship between the variables, using as a measure of distance the Spearman absolute correlation and highlighting significance by calculating correlation *P* values. The choice of analyzing absolute correlation values was motivated by the purpose of this work, which is uncovering the association between different variables, rather than underlying their direction. The figure was generated using Seaborn (version 0.13.2) with the clustermap function, setting average as the linkage method. The default correlation metric was modified to use Spearman instead of Pearson, adaptation implemented using Scipy (version 1.13.0). Then, a Principal Component Analysis was conducted using Scikit-Learn (version 1.2.2). The scatterplot of the first two principal components, generated with Seaborn, highlights the condition of the mouse with variations in both color and shape.

Further analyses were conducted to assess the predictive power of each variable. We first fitted three classification models—Logistic Regression, Random Forest, and Support Vector Machine (SVM)—all of which were implemented through Scikit-Learn (version 1.2.2). To optimize model performance, we applied Grid Search from Scikit-Learn (version 1.2.2) using 5-fold cross-validation and accuracy as the evaluation metric. Logistic Regression classifier was set to use a multinomial loss function and the SAGA solver. Classification was carried out using elastic net as a regularization method, with regularization strength ranging from 0.01 to 1 and the proportion of L1 regularization ranging from 0 to 1, both in increments of 0.1. Some Random Forest’s hyperparameters were tuned as well. In particular, the number of estimators, with options 100, 200, and 300; the maximum depth of the trees, None, 10, 20, or 30; the minimum number of samples required to split a node, 2, 5, or 10; and the minimum number of samples required to be a leaf node, 1, 2, or 4. Finally, we selected the best option for four SVM’s hyperparameters. Specifically, we selected the regularization parameter among four values (0.1, 1, 10, and 100); we explored three different kernel functions – Linear, Radial Basis Function, Polynomial Kernel - with the latter further tuned using 2, 3, and 4 degrees; and we examined the gamma parameter with the options scale and auto. To obtain a cumulative measure, we ranked the feature importance according to the predictive power, which is a weighted average of the other values, with the accuracies employed as weights.

### Statistical analyses

Bar graphs are shown as mean ± standard error of the mean (SEM). P-values were calculated by the GraphPad Prism 10 for Mac, GraphPad Software (www.graphpad.com), using either two-tailed unpaired *t* test or one-way ANOVA, as indicated in the figure legends. *P* values of RNA-seq and pathways analysis were calculated by DESeq2, fsgea, clusterProfiler, and adjusted by Benjamini–Hochberg or Wald test for DESeq2.

For experiments involving animals, the number of mice required was determined through power analyses, ensuring a statistical power of 80% and a significance level of *P* < 0.05. Sample size calculations were performed using GPower, based on preliminary data and expected effect sizes for the parameters central to our study. Animals were allocated to treatment groups in a cage-balanced manner, with treatments distributed across different cages to minimize cage effects and subjective allocation bias; formal computer-based randomization was not applied. No animals were deliberately excluded from the analysis, and no group-specific mortality was observed.

### Graphics

Figures 2A, 8B, EV2A, and the visual abstract graphics were created with BioRender.com.

## Supplementary information

Peer Review File

Dataset EV1

Dataset EV2

Dataset EV3

Dataset EV4

Source data Fig. 1

Source data Fig. 2

Source data Fig. 3

Source data Fig. 6

Source data Fig. 5

Source data Fig. 8

Expanded View Figures

## Data Availability

All raw RNA-seq data are available in the E-MTAB-14908 and E-MTAB-17005 datasets in the EMBL-EBI ENA database. Code, scripts, and computational models used for classification and machine learning analysis are available at the following link: https://zenodo.org/records/19706924. The source data of this paper are collected in the following database record: biostudies:S-SCDT-10_1038-S44321-026-00500-x.
