# Supplementary material for: The telomeric DNA damage response as a therapeutic target in idiopathic pulmonary fibrosis
Source: EMBO Mol Med. 2026 Aug 12;18(9):3704–44. doi: 10.1038/s44321-026-00500-x (PMC13562540; doi:10.1038/s44321-026-00500-x)
Supplement: Supplementary file 1 [file 44321_2026_500_MOESM12_ESM.pdf]

Expanded View Figures

Figure EV1. G3 *Terc*$^{-/-}$ mice exhibit telomere shortening, increased tdincRNAs, and lung pathology without changes in body weight.

(**A**) Lungs from wild-type or G3 *Terc*$^{-/-}$ mice were collected at 3 months of age. Relative telomere length in lung samples from mice, as measured by qPCR ($n = 5$ mice per group). The mouse actin gene was used as a normalizer. (**B**) Body weight of mice of the indicated genotype at 3 months of age (wild-type $n = 15$ mice, G3 *Terc*$^{-/-}$ $n = 18$ mice). (**C**) Quantification of telomeric damage-induced non-coding RNAs (tdincRNAs), both G-rich and C-rich strands, measured by strand-specific RT–qPCR, in the lungs of young (5-month-old) mice of the indicated genotype (wild-type $n = 7$ mice, G3 *Terc*$^{-/-}$ $n = 8$ mice). (**D**) Representative Hematoxylin and Eosin (H&E)-stained whole-lung sections from mice of the indicated age and genotype. Scale bar, 1000 µm. (**E**, **F**) For each of the indicated morphological parameters evaluated by H&E staining, a score was blindly assigned by a chartered pathologist and used to calculate the total pathological score (young: wild-type $n = 12$ mice, G3 *Terc*$^{-/-}$ $n = 14$ mice; adult: control $n = 4$ mice, G3 *Terc*$^{-/-}$ $n = 3$ mice). (**G**) Representative Masson's Trichrome-stained whole-lung sections from mice of the indicated age and genotype. Scale bar, 1000 µm. For all graphs, a two-tailed unpaired *t* test was applied. All data are presented as mean ± SEM.

© The Author(s)

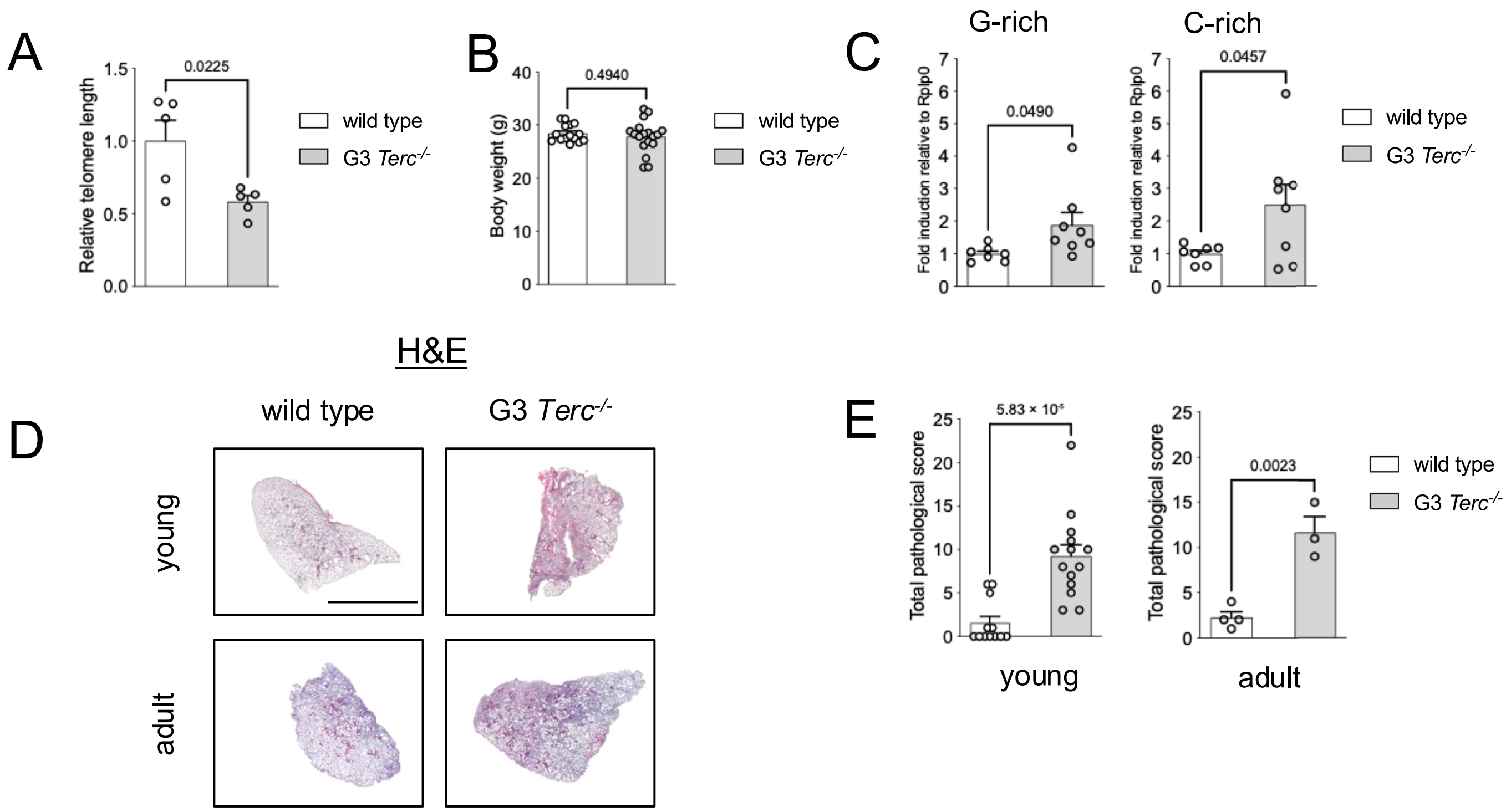

F

	young				adults			
	wild type (N=12)		G3 *Terc-/-* (N=14)		wild type (N=4)		G3 *Terc-/-* (N=3)	
	Average	SEM	Average	SEM	Average	SEM	Average	SEM
Inter-alveolar septal thickening	0.58	0.26	2.36	0.46	0.50	0.29	2.67	0.67
Interstitial cellularity increase	0.50	0.26	2.43	0.33	0.75	0.25	2.67	0.67
Intra-alveolar inflammatory infiltration	0.00	0.00	0.50	0.23	0.00	0.00	1.00	0.00
Interstitial inflammatory infiltration	0.17	0.11	1.86	0.27	0.75	0.48	3.33	0.67
Erythrocyte extravasation	0.33	0.14	2.07	0.38	0.25	0.25	2.00	0.00

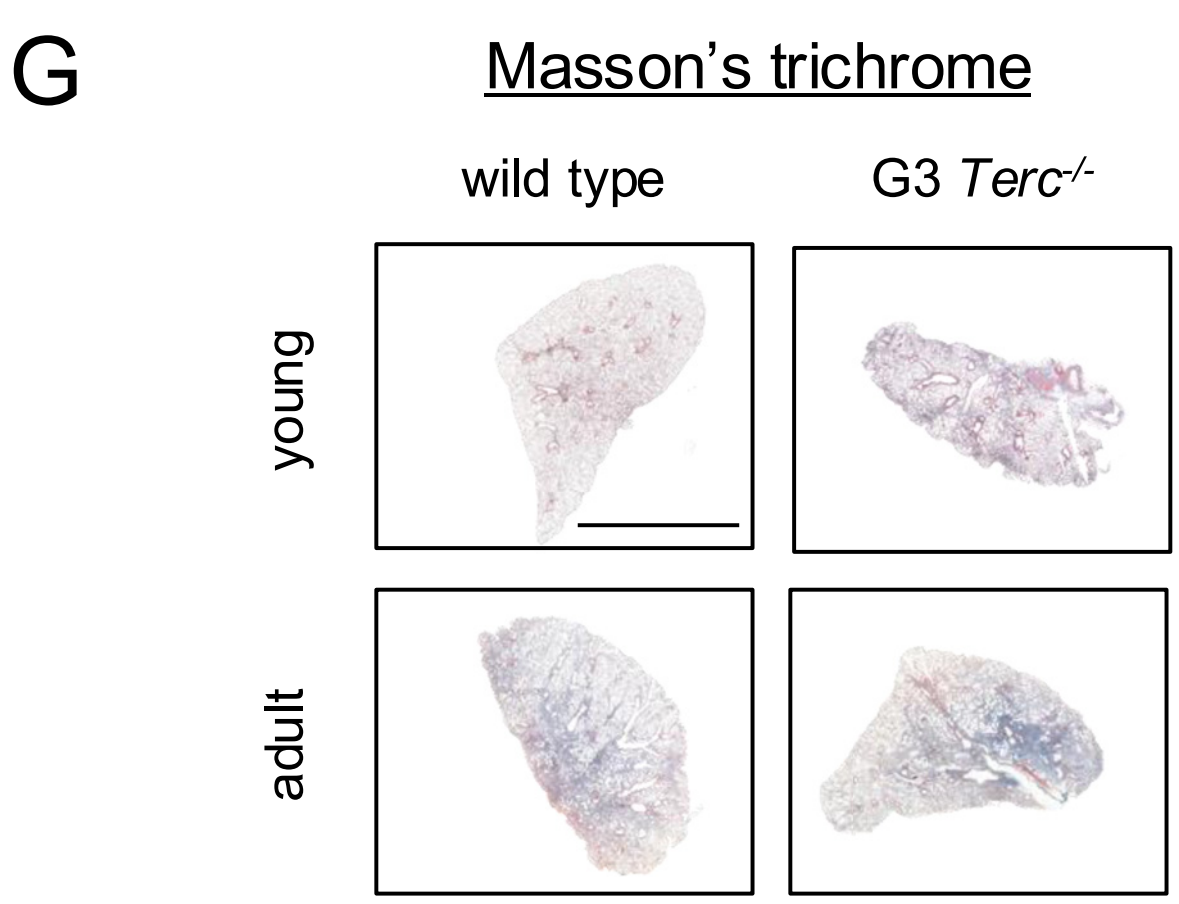

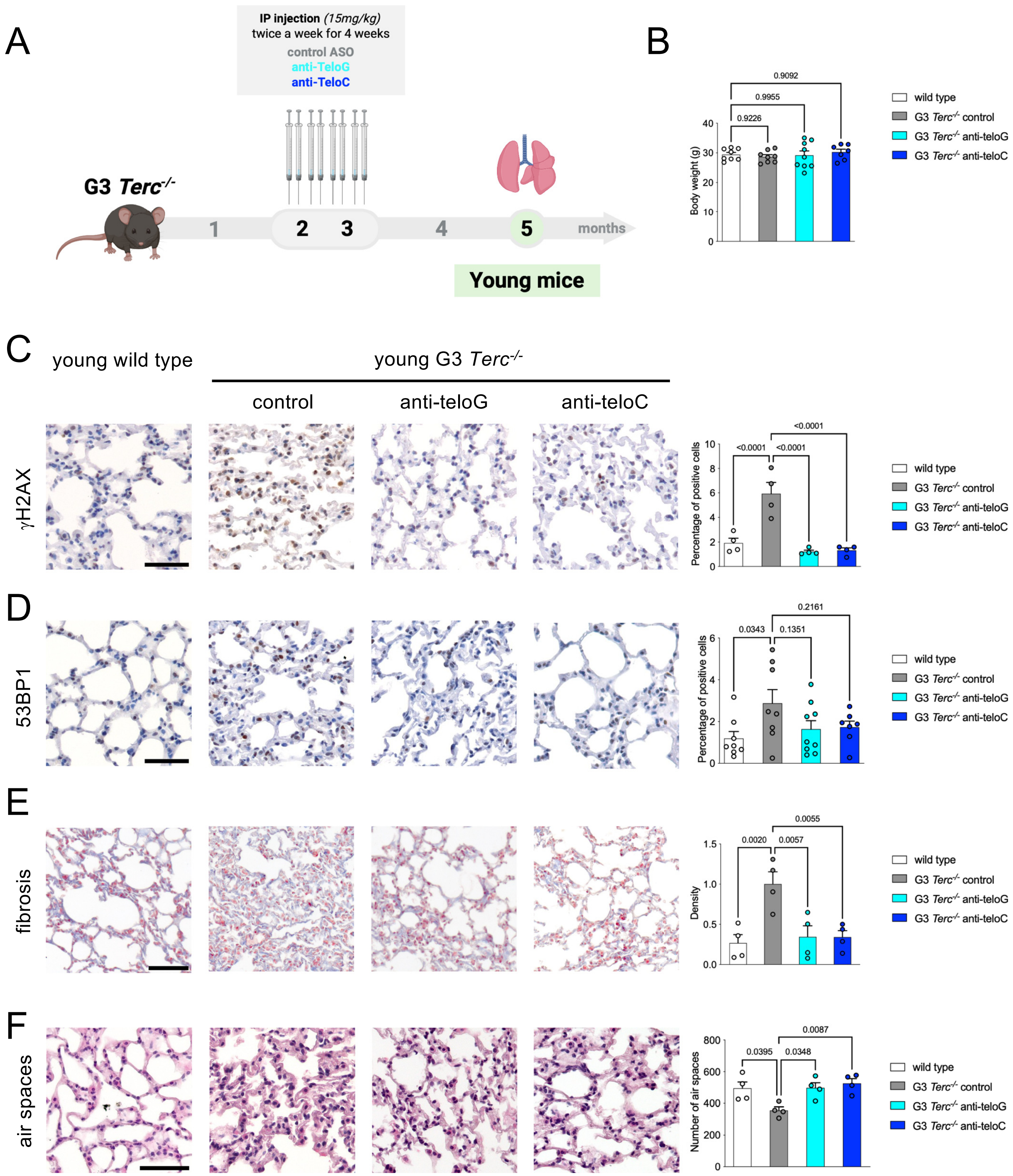

A
IP injection (15mg/kg)
twice a week for 4 weeks
control ASO
anti-TeloG
anti-TeloC
G3 Terc-/-
1
2 3
4
5
months
Young mice
B
Body weight (g)
0.9092
0.9955
0.9226
wild type
G3 Terc-/- control
G3 Terc-/- anti-teloG
G3 Terc-/- anti-teloC
C
young wild type
young G3 Terc-/-
control
anti-teloG
anti-teloC
γH2AX
Percentage of positive cells
<0.0001
<0.0001
<0.0001
D
53BP1
0.2161
0.0343
0.1351
E
fibrosis
Density
0.0055
0.0020
0.0057
F
air spaces
Number of air spaces
0.0087
0.0395
0.0348

Figure EV2. tASOs treatment reduces markers of DDR activation and ameliorates tissue histopathology of the lungs of young G3 *Terc*$^{-/-}$ mice.

(**A**) Schematic of in vivo ASOs treatments. 2-month-old G3 *Terc*$^{-/-}$ mice were intraperitoneally injected with 15 mg/kg of control, anti-teloG, or anti-teloC ASOs twice a week for 4 weeks (eight injections in total), and sacrificed to collect lungs 2 months after the last injection, at 5 (young) months of age, together with untreated age-matched wild-type mice. (**B**) Body weight of mice with the indicated genotype and treatment (wild-type $n = 8$ mice, G3 *Terc*$^{-/-}$ control $n = 8$ mice; G3 *Terc*$^{-/-}$ anti-teloG $n = 9$ mice, G3 *Terc*$^{-/-}$ anti-teloC $n = 7$ mice). (**C**, **D**) Representative images and quantification of the percentage of γH2AX- or 53BP1-positive cells as detected by IHC staining (γH2AX: wild-type $n = 4$ mice, G3 *Terc*$^{-/-}$ control $n = 4$ mice; G3 *Terc*$^{-/-}$ anti-teloG $n = 4$ mice, G3 *Terc*$^{-/-}$ anti-teloC $n = 4$ mice; 53BP1: wild-type $n = 8$ mice, G3 *Terc*$^{-/-}$ control $n = 8$ mice; G3 *Terc*$^{-/-}$ anti-teloG $n = 9$ mice, G3 *Terc*$^{-/-}$ anti-teloC $n = 7$ mice). Original magnification, ×200. Scale bar, 100 µm. (**E**) Representative images and quantification of fibrosis as measured by Masson's Trichrome staining density ($n = 4$ mice per group). Original magnification, ×200. Scale bar, 100 µm. (**F**) Representative images and quantification of the number of air spaces per field ($n = 4$ mice per group). Original magnification, ×200. Scale bar, 100 µm. For all graphs, ordinary one-way ANOVA followed by Dunnett's multiple comparison test was applied. All data are presented as mean ± SEM.

 © The Author(s)

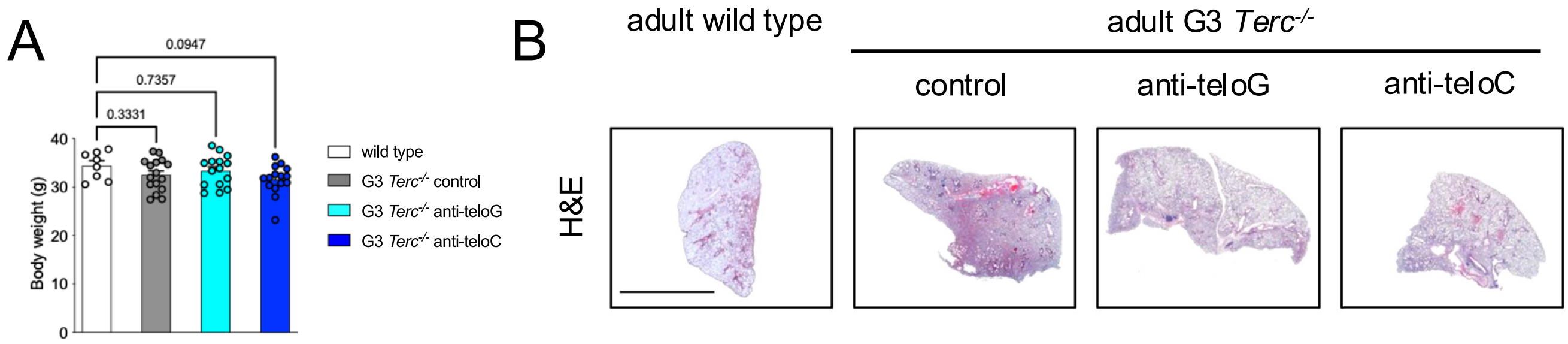

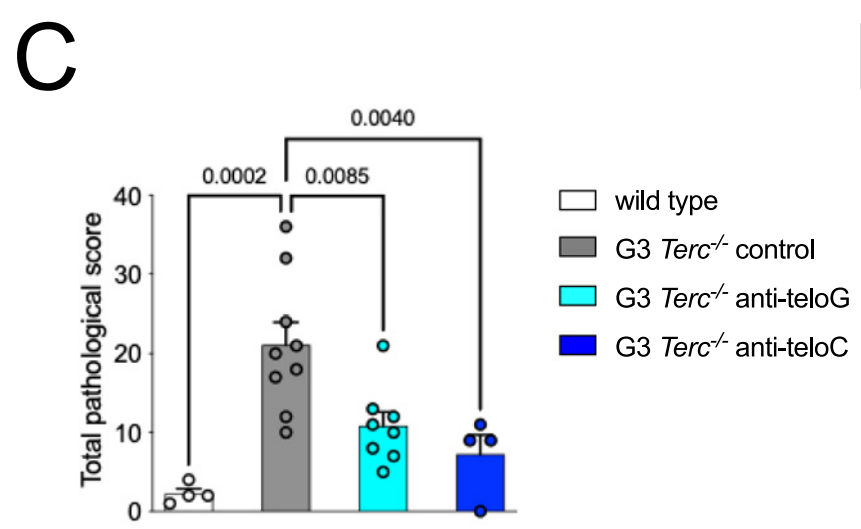

D

	Adult wild type (N=4)		Adult G3 Terc-/- control (N=9)		Adult G3 Terc-/- anti-teloG (N=8)		Adult G3 Terc-/- anti-teloC (N=4)	
	Average	SEM	Average	SEM	Average	SEM	Average	SEM
Inter-alveolar septal thickening	0.50	0.29	5.44	0.88	3.00	0.53	1.50	0.65
Interstitial cellularity increase	0.75	0.25	5.11	0.89	3.13	0.58	1.50	0.50
Intra-alveolar inflammatory infiltration	0.00	0.00	2.78	0.62	1.13	0.35	1.00	0.41
Interstitial inflammatory infiltration	0.75	0.48	5.00	0.67	2.25	0.31	1.25	0.48
Erythrocyte extravasation	0.25	0.25	2.78	0.55	1.38	0.26	2.00	0.82

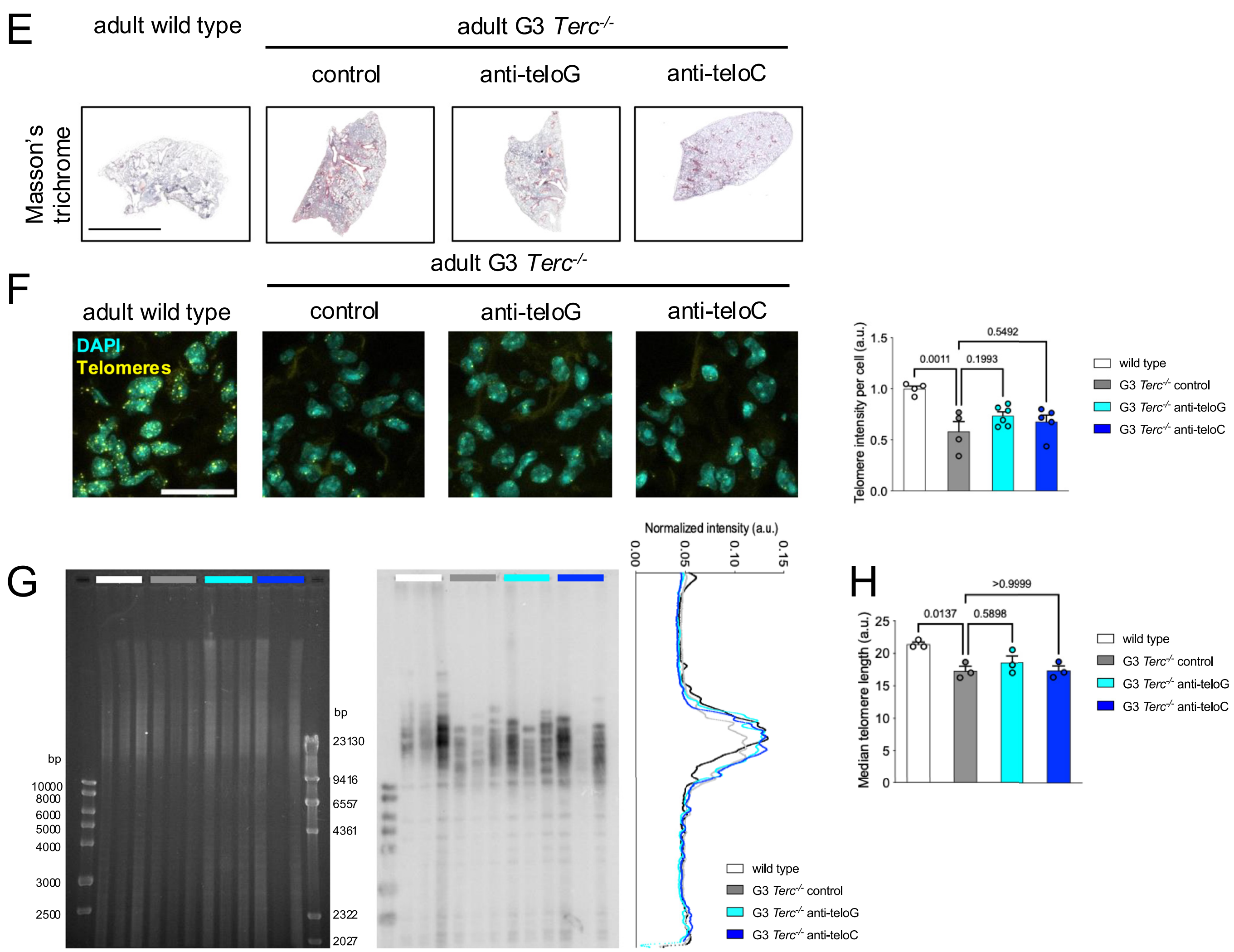

Figure EV3. tDDR inhibition ameliorates the pathological score of the lungs of adult G3 *Terc*$^{-/-}$ mice without changes in body weight and without affecting telomere length.

G3 *Terc*$^{-/-}$ mice were treated as described in Fig. 2A and sacrificed at 12 (adult) months of age, together with age-matched wild-type mice. Lungs were collected for analysis. (**A**) Body weight of mice with the indicated genotype and treatment. (wild-type $n = 8$ mice, G3 *Terc*$^{-/-}$ control $n = 16$ mice; G3 *Terc*$^{-/-}$ anti-teloG $n = 15$ mice, G3 *Terc*$^{-/-}$ anti-teloC $n = 15$ mice). (**B**) Representative Hematoxylin and Eosin (H&E)-stained whole-lung sections from mice treated as indicated. Scale bar, 1000 µm. (**C**, **D**) For each of the indicated morphological parameters evaluated by H&E, a score was assigned blindly and used to calculate the pathological score (see "Methods" section) (wild-type $n = 4$ mice, G3 *Terc*$^{-/-}$ control $n = 9$ mice; G3 *Terc*$^{-/-}$ anti-teloG $n = 8$ mice, G3 *Terc*$^{-/-}$ anti-teloC $n = 4$ mice). (**E**) Representative Masson's trichrome-stained whole-lung sections from mice treated as indicated. Scale bar, 1000 µm. (**F**) Representative images and quantification of the fluorescence in situ hybridization (FISH) staining and telomeric PNA probe signal quantification expressed in arbitrary units, a.u. (wild-type $n = 4$ mice, G3 *Terc*$^{-/-}$ control $n = 4$ mice; G3 *Terc*$^{-/-}$ anti-teloG $n = 6$ mice, G3 *Terc*$^{-/-}$ anti-teloC $n = 5$ mice). Scale bar, 20 µm. (**G**) Telomere restriction fragment (TRF) analysis of genomic DNA. Left: ethidium bromide staining showing total DNA. Right: telomere-specific hybridization using a radiolabeled telomeric probe. The quantification shows the normalized telomeric probe signal intensity (**H**) Median telomere length expressed in arbitrary units (a.u.), as determined from TRF analysis ($n = 3$ mice per group). For all graphs, ordinary one-way ANOVA followed by Dunnett's multiple comparison test was applied. All data are presented as mean ± SEM.

 © The Author(s)

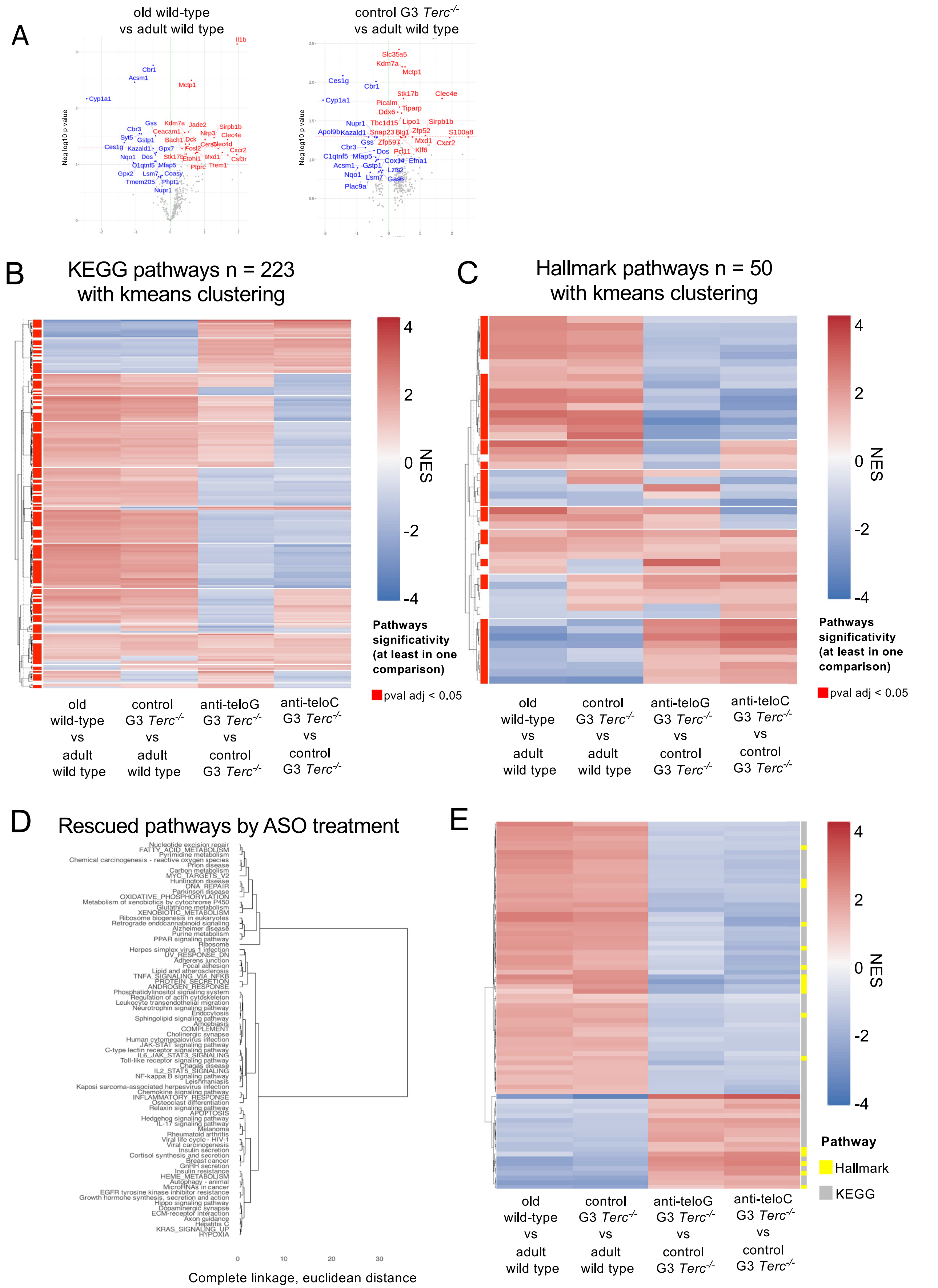
A
old wild-type
vs adult wild type
control G3 Terc-/-
vs adult wild type
Neg log10 p value
B
KEGG pathways n = 223
with kmeans clustering
C
Hallmark pathways n = 50
with kmeans clustering
NES
Pathways significativity (at least in one comparison)
pval adj < 0.05
old wild-type vs adult wild type
control G3 Terc-/- vs adult wild type
anti-teloG G3 Terc-/- vs control G3 Terc-/-
anti-teloC G3 Terc-/- vs control G3 Terc-/-
D
Rescued pathways by ASO treatment
Complete linkage, euclidean distance
E
Pathway
Hallmark
KEGG

Figure EV4. tDDR inhibition reverses the altered transcriptional profiles of lungs of adult G3 *Terc*$^{-/-}$ mice.

G3 *Terc*$^{-/-}$ mice were treated as described in Fig. 2A and sacrificed at 12 (adult) months of age; untreated wild-type mice were sacrificed at 12 (adult) or 22 (old) months of age. Lungs were collected for analysis. (**A**) Volcano plots highlighting the top 20 upregulated and downregulated genes in the indicated comparisons (logFC and *P* value were calculated with DESeq2 using the Wald test). (**B**, **C**) Heatmaps of the Normalized Enrichment Score for KEGG (**B**, $n = 223$) and Hallmark (**C**, $n = 50$) pathways. Pathway enrichment analysis for the indicated comparisons, and a hierarchical clustering and k-means ($k = 14$) clustering were performed. Pathways were annotated on the left in red when significantly modified (*P* value adjusted by Benjamini-Hochberg test) in at least one comparison. (**D**, **E**) Dendogram and heatmap of the pathways (Hallmark: yellow, uppercase; KEGG: gray, lowercase) reversed by tASOs treatments. A reversed pathway is a pathway significantly deregulated (*P* value adjusted <0.05) in old wild-type vs adult wild-type mice and reversed in anti-teloG G3 *Terc*$^{-/-}$ vs control G3 *Terc*$^{-/-}$ and anti-teloC G3 *Terc*$^{-/-}$ vs control G3 *Terc*$^{-/-}$ ($n = 3$ mice for old wild-type, $n = 5$ mice for adult wild-type, $n = 5$ mice for control G3 *Terc*$^{-/-}$, $n = 5$ mice for anti-teloG G3 *Terc*$^{-/-}$, $n = 5$ mice for anti-teloC G3 *Terc*$^{-/-}$).

 © The Author(s)

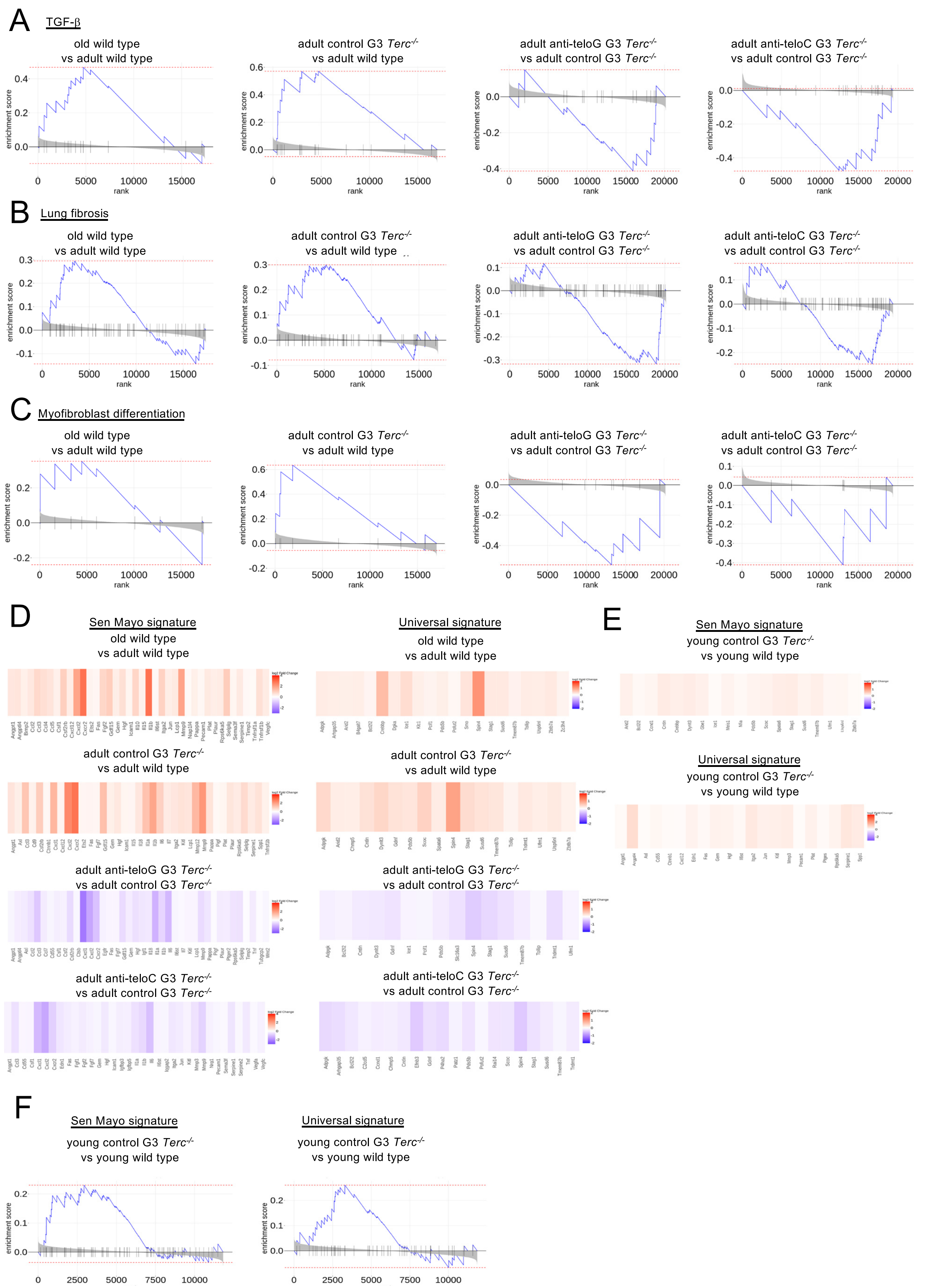
A
TGF-β
old wild type vs adult wild type
adult control G3 Terc-/- vs adult wild type
adult anti-teloG G3 Terc-/- vs adult control G3 Terc-/-
adult anti-teloC G3 Terc-/- vs adult control G3 Terc-/-
B
Lung fibrosis
old wild type vs adult wild type
adult control G3 Terc-/- vs adult wild type
adult anti-teloG G3 Terc-/- vs adult control G3 Terc-/-
adult anti-teloC G3 Terc-/- vs adult control G3 Terc-/-
C
Myofibroblast differentiation
old wild type vs adult wild type
adult control G3 Terc-/- vs adult wild type
adult anti-teloG G3 Terc-/- vs adult control G3 Terc-/-
adult anti-teloC G3 Terc-/- vs adult control G3 Terc-/-
enrichment score
rank
D
Sen Mayo signature
old wild type vs adult wild type
adult control G3 Terc-/- vs adult wild type
adult anti-teloG G3 Terc-/- vs adult control G3 Terc-/-
adult anti-teloC G3 Terc-/- vs adult control G3 Terc-/-
Universal signature
old wild type vs adult wild type
adult control G3 Terc-/- vs adult wild type
adult anti-teloG G3 Terc-/- vs adult control G3 Terc-/-
adult anti-teloC G3 Terc-/- vs adult control G3 Terc-/-
E
Sen Mayo signature
young control G3 Terc-/- vs young wild type
Universal signature
young control G3 Terc-/- vs young wild type
F
Sen Mayo signature
young control G3 Terc-/- vs young wild type
Universal signature
young control G3 Terc-/- vs young wild type
enrichment score
rank

Figure EV5. tDDR inhibition reverses fibrosis and senescence signatures of lungs of adult G3 *Terc*$^{-/-}$ mice.

(**A–C**) GSEA enrichment plot for the TGF-β, lung fibrosis, and myofibroblast differentiation pathways for the indicated comparisons. (**D**, **E**) Differential Gene Expression (logFC) of Sen Mayo and Universal signature. (**F**) GSEA enrichment plots for the Sen Mayo and Universal signatures for the indicated comparisons.

 © The Author(s)

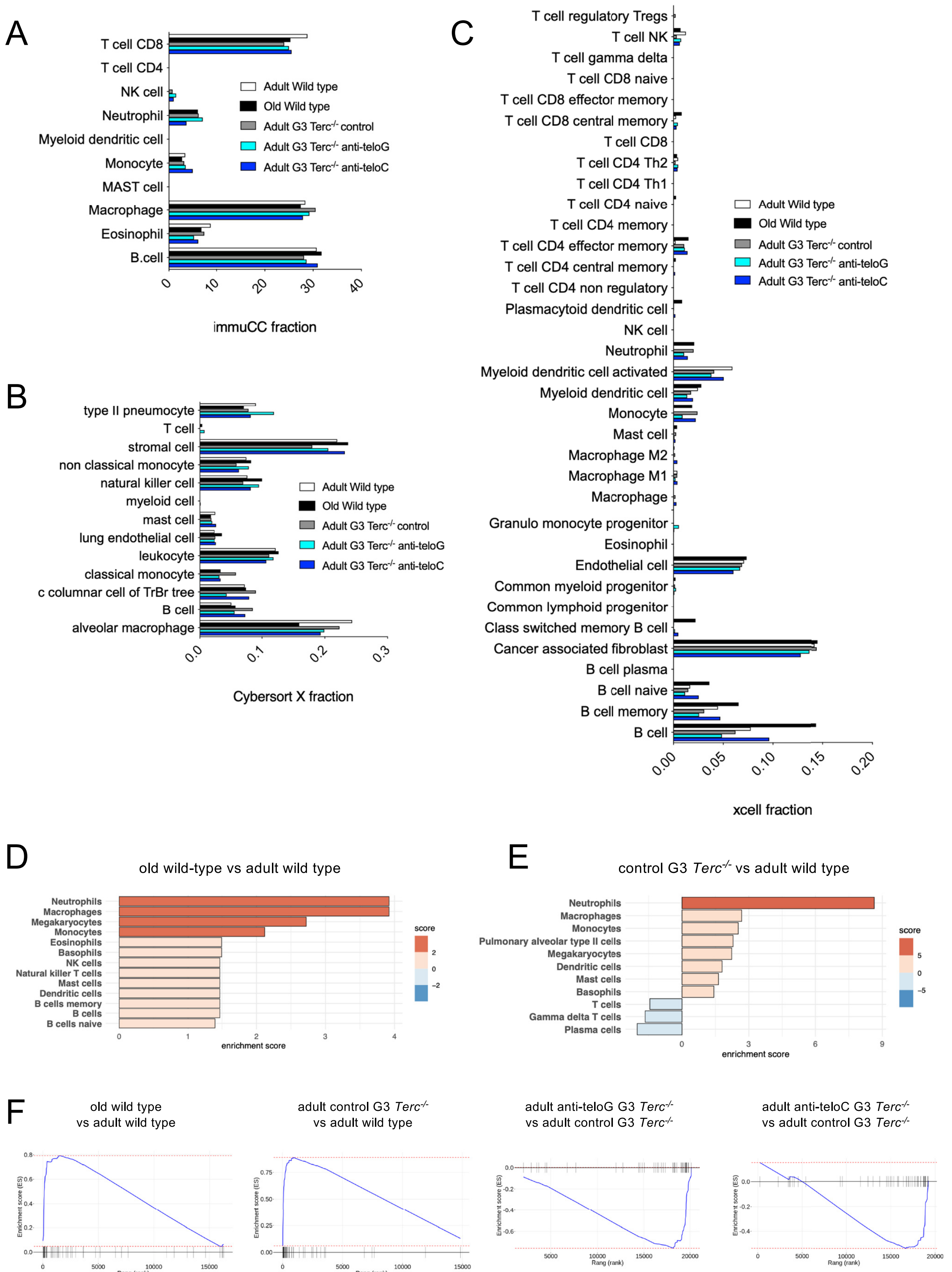
A
T cell CD8
T cell CD4
NK cell
Neutrophil
Myeloid dendritic cell
Monocyte
MAST cell
Macrophage
Eosinophil
B.cell
Adult Wild type
Old Wild type
Adult G3 Terc-/- control
Adult G3 Terc-/- anti-teloG
Adult G3 Terc-/- anti-teloC
0
10
20
30
40
immuCC fraction
B
type II pneumocyte
T cell
stromal cell
non classical monocyte
natural killer cell
myeloid cell
mast cell
lung endothelial cell
leukocyte
classical monocyte
c columnar cell of TrBr tree
B cell
alveolar macrophage
Adult Wild type
Old Wild type
Adult G3 Terc-/- control
Adult G3 Terc-/- anti-teloG
Adult G3 Terc-/- anti-teloC
0.0
0.1
0.2
0.3
Cybersort X fraction
C
T cell regulatory Tregs
T cell NK
T cell gamma delta
T cell CD8 naive
T cell CD8 effector memory
T cell CD8 central memory
T cell CD8
T cell CD4 Th2
T cell CD4 Th1
T cell CD4 naive
T cell CD4 memory
T cell CD4 effector memory
T cell CD4 central memory
T cell CD4 non regulatory
Plasmacytoid dendritic cell
NK cell
Neutrophil
Myeloid dendritic cell activated
Myeloid dendritic cell
Monocyte
Mast cell
Macrophage M2
Macrophage M1
Macrophage
Granulo monocyte progenitor
Eosinophil
Endothelial cell
Common myeloid progenitor
Common lymphoid progenitor
Class switched memory B cell
Cancer associated fibroblast
B cell plasma
B cell naive
B cell memory
B cell
Adult Wild type
Old Wild type
Adult G3 Terc-/- control
Adult G3 Terc-/- anti-teloG
Adult G3 Terc-/- anti-teloC
0.00
0.05
0.10
0.15
0.20
xcell fraction
D
old wild-type vs adult wild type
Neutrophils
Macrophages
Megakaryocytes
Monocytes
Eosinophils
Basophils
NK cells
Natural killer T cells
Mast cells
Dendritic cells
B cells memory
B cells
B cells naive
score
2
0
−2
0
1
2
3
4
enrichment score
E
control G3 Terc-/- vs adult wild type
Neutrophils
Macrophages
Monocytes
Pulmonary alveolar type II cells
Megakaryocytes
Dendritic cells
Mast cells
Basophils
T cells
Gamma delta T cells
Plasma cells
score
5
0
−5
0
3
6
9
enrichment score
F
old wild type
vs adult wild type
adult control G3 Terc-/-
vs adult wild type
adult anti-teloG G3 Terc-/-
vs adult control G3 Terc-/-
adult anti-teloC G3 Terc-/-
vs adult control G3 Terc-/-
Enrichment score (ES)
Rang (rank)

Figure EV6. Neutrophil transcriptional signatures are elevated in old and G3 *Terc*$^{-/-}$ lungs and reduced by tASOs.

(**A–C**) Average deconvolution fractions across samples. Bars represent mean deconvolution scores within each group (old wild-type, adult wild-type, control G3 *Terc*$^{-/-}$, anti-teloG G3 *Terc*$^{-/-}$, and anti-teloC G3 *Terc*$^{-/-}$). (**D**) Enriched cytotypes from the rank-based gene set enrichment analysis (GSEA, clusterProfiler, permutation-based enrichment test) on old wild-type and adult wild-type. Bar length reflects the significance level (log10 adjusted *P* value). The significant cytotypes are all positively associated with the old wild-type condition. (**E**) Enriched cytotypes from the rank-based gene set enrichment analysis (GSEA, clusterProfiler, permutation-based enrichment test) on old wild-type and control G3 *Terc*$^{-/-}$ compared to adult wild-type. Bar length reflects the significance level (log10 adjusted *P* value). (**F**) GSEA plots showing the Neutrophils associations across different DEG lists. Neutrophils are positively enriched in old wild-type and control G3 *Terc*$^{-/-}$ samples (both versus adult wild-type). This is in contrast with the downregulation of neutrophil-related transcriptional activity observed in the anti-teloG G3 *Terc*$^{-/-}$, and anti-teloC G3 *Terc*$^{-/-}$ (both versus control G3 *Terc*$^{-/-}$).

 © The Author(s)

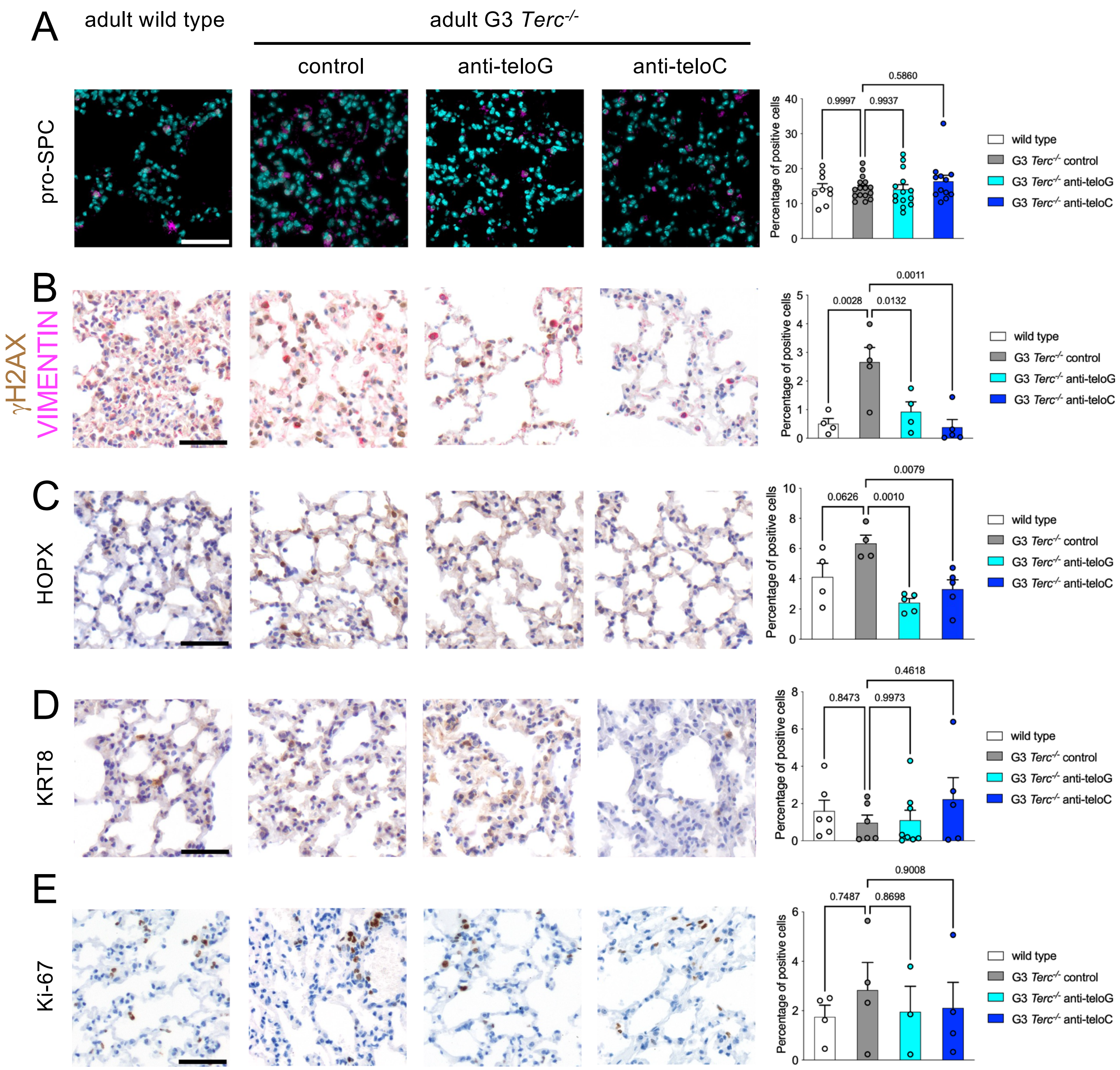

Figure EV7. tASOs treatment attenuates DDR also in mesenchymal cells and promotes restoration of alveolar homeostasis.

(**A**) Representative images and quantification of the percentage of ATII cells, as detected by the pro-SPC marker by immunofluorescence (wild-type $n = 9$ mice, G3 *Terc*$^{-/-}$ control $n = 16$ mice; G3 *Terc*$^{-/-}$ anti-teloG $n = 14$ mice, G3 *Terc*$^{-/-}$ anti-teloC $n = 12$ mice). (**B**) Representative images and quantification of the percentage of γH2AX-positive cells (brown signal) among all VIMENTIN-positive cells (pink signal), as determined by double IHC staining (wild-type $n = 4$ mice, G3 *Terc*$^{-/-}$ control $n = 5$ mice; G3 *Terc*$^{-/-}$ anti-teloG $n = 4$ mice, G3 *Terc*$^{-/-}$ anti-teloC $n = 5$ mice). (**C–E**) Representative images and quantification of the percentage of HOPX-, KRT8-, or Ki-67-positive cells as detected by IHC staining in the lungs of adult mice treated as indicated (HOPX: wild-type $n = 4$ mice, G3 *Terc*$^{-/-}$ control $n = 4$ mice; G3 *Terc*$^{-/-}$ anti-teloG $n = 5$ mice, G3 *Terc*$^{-/-}$ anti-teloC $n = 5$ mice; KRT8: wild-type $n = 6$ mice, G3 *Terc*$^{-/-}$ control $n = 6$ mice; G3 *Terc*$^{-/-}$ anti-teloG $n = 8$ mice, G3 *Terc*$^{-/-}$ anti-teloC $n = 5$ mice; KI67: wild-type $n = 4$ mice, G3 *Terc*$^{-/-}$ control $n = 4$ mice; G3 *Terc*$^{-/-}$ anti-teloG $n = 3$ mice, G3 *Terc*$^{-/-}$ anti-teloC $n = 4$ mice). Original magnification, ×200. Scale bar, 100 µm. For all graphs, ordinary one-way ANOVA followed by Dunnett's multiple comparison test was applied. All data are presented as mean ± SEM.

© The Author(s)

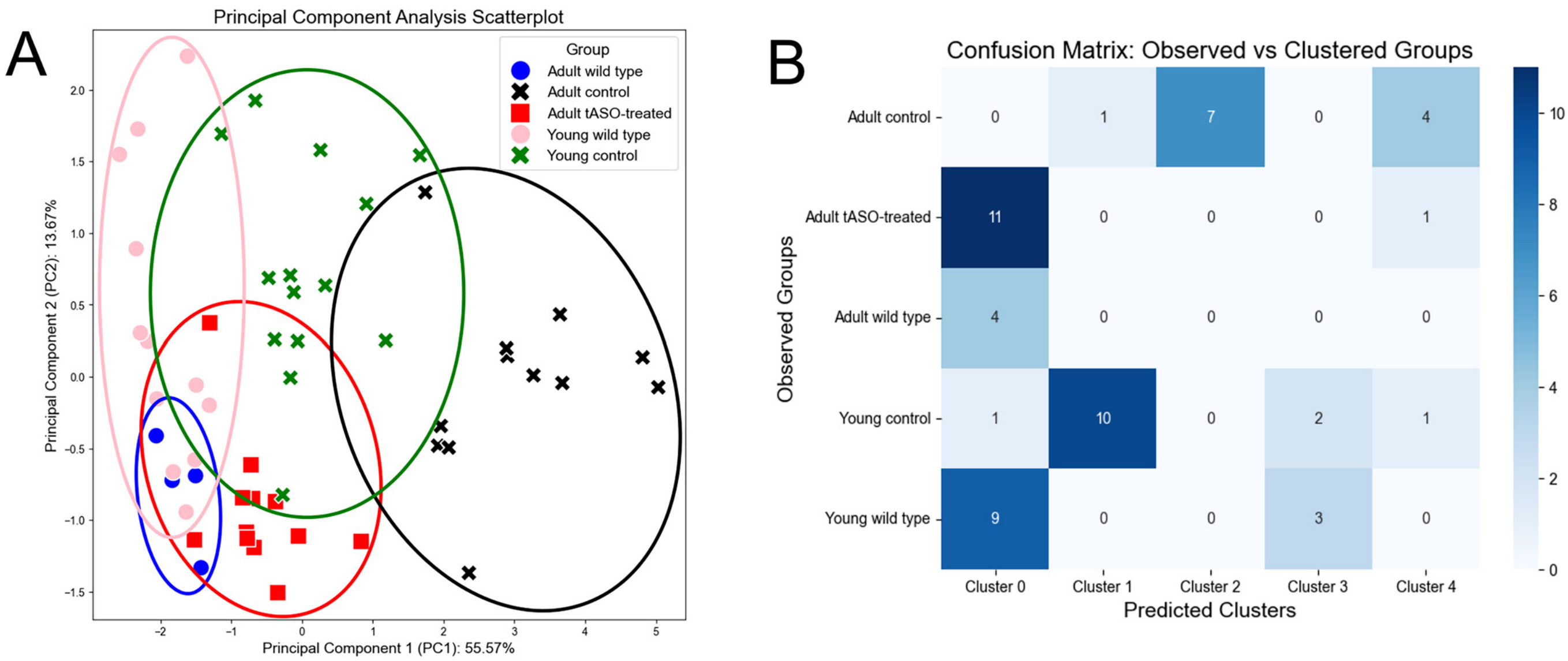

Figure EV8. PCA of multiparametric features shows a shift toward a youthful state following tASOs treatment.

In all, 28 adult (12-month-old) and 26 young (3-month-old) mice scored for 7 variables were used for the following analysis. (**A**) Multivariable principal component analysis (PCA). The 2 axes explained 69.24% of the variance (55.57% PC1 and 13.67% PC2). Based on PCA, the mice were segregated into 5 groups (blue: adult wild-type mice, black: adult control G3 *Terc*$^{-/-}$ mice, red: adult tASOs-treated G3 *Terc*$^{-/-}$ mice, pink: young wild-type mice, green: young G3 *Terc*$^{-/-}$mice). (**B**) Heatmap of the confusion matrix for the aggregative clustering classification of the mice (observed groups vs predicted groups).

© The Author(s)

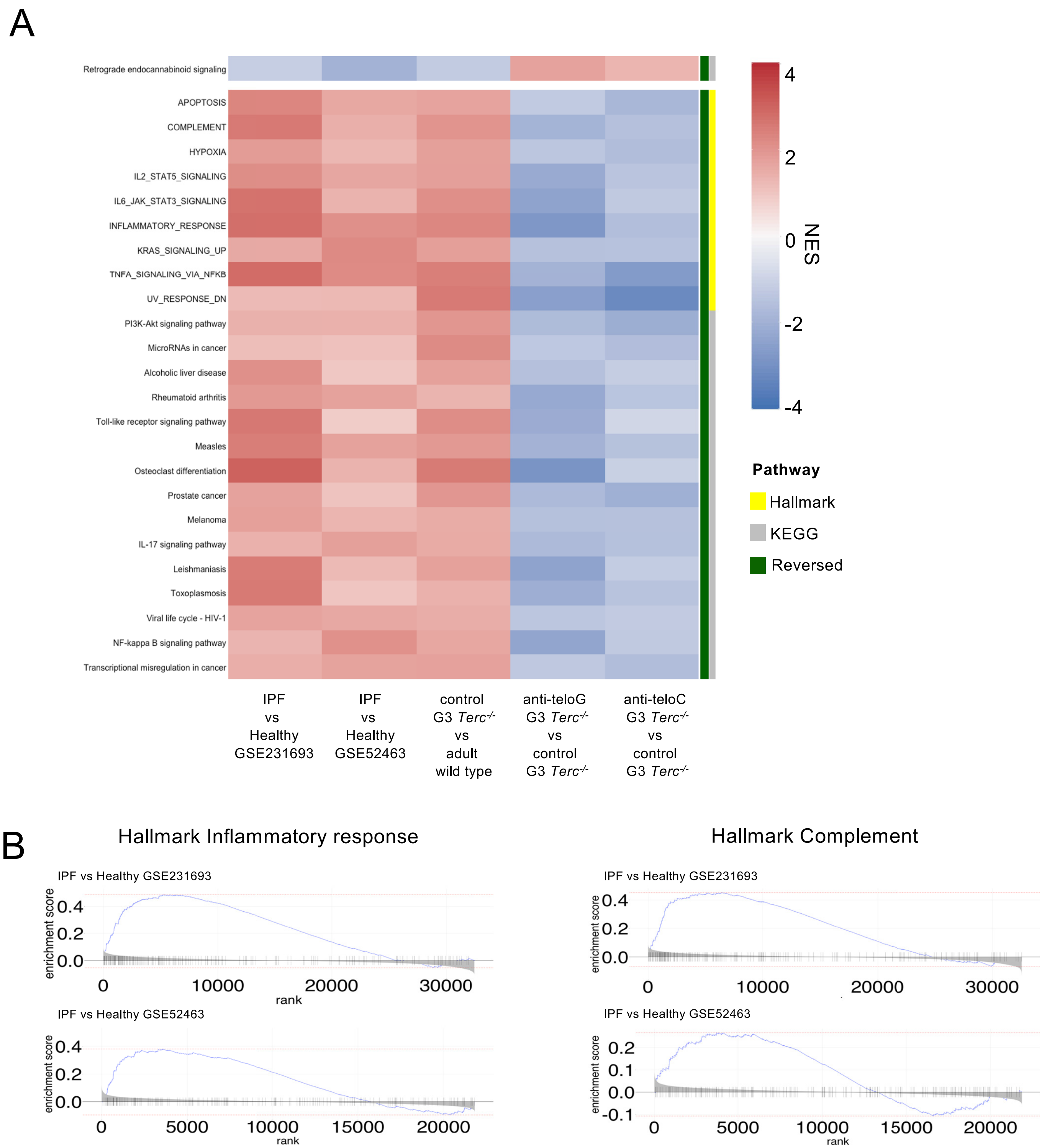

Figure EV9. tDDR inhibition normalizes pathways in the lungs of adult G3 *Terc*$^{-/-}$ mice, which are altered in human IPF patients, including inflammatory signaling.

(**A**) Heatmap of the Normalized Enrichment Score for the reversed KEGG pathways (gray, lowercase) and Hallmark pathways (yellow, uppercase) in human and control G3 *Terc*$^{-/-}$ mice for the indicated comparisons. (**B**) GSEA plot for the Inflammatory response pathway and Complement pathways for the indicated comparisons.

© The Author(s)
