# Supplementary material for: The telomeric DNA damage response as a therapeutic target in idiopathic pulmonary fibrosis
Source: EMBO Mol Med. 2026 Aug 12;18(9):3704–44. doi: 10.1038/s44321-026-00500-x (PMC13562540; doi:10.1038/s44321-026-00500-x)
Supplement: Supplementary file 2 [file 44321_2026_500_MOESM1_ESM.pdf]

EMBO Molecular Medicine

The telomeric DNA damage response as a therapeutic target in Idiopathic Pulmonary Fibrosis

Francesca Rossiello, Giada Cicio, Sara Sepe, Alexandra Mancheno-Ferris, Valeria Cancila, Arianna Castellini, Federica Rey, Giorgio Bertolazzi, Alessia Oppezzo, Eugenia Marinelli, Matteo Cabrini, Alessia di Lillo, Sara Tavella, Ilaria Rosso, Francesca Buffa, Claudio Tripodo, and Fabrizio d'Adda di Fagagna

Corresponding authors: Fabrizio d'Adda di Fagagna (fabrizio.dadda@igm.cnr.it)

Review Timeline:

Submission Date:	20th May 25
Editorial Decision:	24th Jun 25
Revision Received:	23rd Apr 26
Editorial Decision:	28th May 26
Revision Received:	8th Jun 26
Editorial Decision:	3rd Jul 26
Revision Received:	16th Jul 26
Accepted:	23rd Jul 26

Editor: Lise Roth

Transaction Report:

(Note: With the exception of the correction of typographical or spelling errors that could be a source of ambiguity, letters and reports are not edited. Depending on transfer agreements, referee reports obtained elsewhere may or may not be included in this compilation. Referee reports are anonymous unless the Referee chooses to sign their reports.)

24th Jun 2025

Dear Dr. d'Adda di Fagagna,

Thank you for submitting your manuscript to EMBO Molecular Medicine, and please accept my apologies for the delay in getting back to you, as one referee needed more time to provide their report.
We have now received feedback from the three reviewers who agreed to evaluate your manuscript. As you will see from the reports below, the referees acknowledge the interest of the study and are overall supporting publication of your work pending appropriate revisions.

Addressing the reviewers' concerns in full will be necessary for further considering the manuscript in our journal, and acceptance of the manuscript will entail a second round of review. EMBO Molecular Medicine encourages a single round of revision only and therefore, acceptance or rejection of the manuscript will depend on the completeness of your responses included in the next, final version of the manuscript. For this reason, and to save you from any frustrations in the end, I would strongly advise against returning an incomplete revision.

If you would like to discuss further the points raised by the referees, I am available to do so via email or video. Let me know if you are interested in this option.

We are expecting your revised manuscript within three months, if you anticipate any delay, please contact us.

When submitting your revised manuscript, please carefully review the instructions that follow below. We perform an initial quality control of all revised manuscripts before re-review; failure to include requested items will delay the evaluation of your revision.

We require:

1) A .docx formatted version of the manuscript text (including legends for main figures, EV figures and tables). Please make sure that the changes are highlighted to be clearly visible.

2) Individual production quality figure files as .eps, .tif, .jpg (one file per figure). For guidance, download the 'Figure Guide PDF' (https://www.embopress.org/page/journal/17574684/authorguide#figureformat).

3) At EMBO Press we ask authors to provide source data for the main figures. Our source data coordinator will contact you to discuss which figure panels we would need source data for and will also provide you with helpful tips on how to upload and organize the files.

4) A .docx formatted letter INCLUDING the reviewers' reports and your detailed point-by-point responses to their comments. As part of the EMBO Press transparent editorial process, the point-by-point response is part of the Review Process File (RPF), which will be published alongside your paper.

5) A complete author checklist, which you can download from our author guidelines (https://www.embopress.org/page/journal/17574684/authorguide#submissionofrevisions). Please insert information in the checklist that is also reflected in the manuscript. The completed author checklist will also be part of the RPF.

6) All Materials and Methods need to be described in the main text using our 'Structured Methods' format. According to this format, the Methods section includes a Reagents and Tools Table (listing key reagents, experimental models, software and relevant equipment and including their sources and relevant identifiers) followed by a Methods and Protocols section describing the methods, ideally using a step-by-step protocol format. The aim is to facilitate adoption of the methodologies across labs. Please download and fill our Reagents and Tools Table template (.docx), which you can find in our author guidelines: https://www.embopress.org/page/journal/14693178/authorguide#structuredmethods.
When submitting your revised manuscript, please do not include the Reagents and Tools Table in the Methods section of the manuscript but upload it as a separate file choosing the file type "Reagent Table".

7) Please note that all corresponding authors are required to supply an ORCID ID for their name upon submission of a revised manuscript.

8) It is mandatory to include a 'Data Availability' section after the Materials and Methods. Before submitting your revision, primary datasets produced in this study need to be deposited in an appropriate public database, and the accession numbers and database listed under 'Data Availability'. Please remember to provide a reviewer password if the datasets are not yet public (see https://www.embopress.org/page/journal/17574684/authorguide#dataavailability).

In case you have no data that requires deposition in a public database, please state so in this section. Note that the Data

Availability Section is restricted to new primary data that are part of this study.

9) For data quantification: please specify the name of the statistical test used to generate error bars and P values, the number (n) of independent experiments (specify technical or biological replicates) underlying each data point and the test used to calculate p-values in each figure legend. The figure legends should contain a basic description of n, P and the test applied. Graphs must include a description of the bars and the error bars (s.d., s.e.m.). Please provide exact p values.

10) Our journal encourages inclusion of *data citations in the reference list* to directly cite datasets that were re-used and obtained from public databases. Data citations in the article text are distinct from normal bibliographical citations and should directly link to the database records from which the data can be accessed. In the main text, data citations are formatted as follows: "Data ref: Smith et al, 2001" or "Data ref: NCBI Sequence Read Archive PRJNA342805, 2017". In the Reference list, data citations must be labeled with "[DATASET]". A data reference must provide the database name, accession number/identifiers and a resolvable link to the landing page from which the data can be accessed at the end of the reference. Further instructions are available at .

11) We replaced Supplementary Information with Expanded View (EV) Figures and Tables that are collapsible/expandable online. EV Figures should be cited as 'Figure EV1, Figure EV2" etc... in the text and their respective legends should be included in the main text after the legends of regular figures.
- For the figures that you do NOT wish to display as Expanded View figures, they should be bundled together with their legends in a single PDF file called *Appendix*, which should start with a short Table of Content. Appendix figures should be referred to in the main text as: "Appendix Figure S1, Appendix Figure S2" etc.
- Additional Tables/Datasets should be labeled and referred to as Table EV1, Dataset EV1, etc. Legends have to be provided in a separate tab in case of .xls files. Alternatively, the legend can be supplied as a separate text file (README) and zipped together with the Table/Dataset file.
See detailed instructions here:

12) The paper explained: EMBO Molecular Medicine articles are accompanied by a summary of the articles to emphasize the major findings in the paper and their medical implications for the non-specialist reader. Please provide a draft summary of your article highlighting
- the medical issue you are addressing,
- the results obtained and
- their clinical impact.
This may be edited to ensure that readers understand the significance and context of the research. Please refer to any of our published articles for an example.

13) Author contributions: CRediT has replaced the traditional author contributions section because it offers a systematic machine readable author contributions format that allows for more effective research assessment. Please remove the Authors Contributions from the manuscript and use the free text boxes beneath each contributing author's name in our system to add specific details on the author's contribution. More information is available in our guide to authors.

14) Disclosure statement and competing interests: We updated our journal's competing interests policy in January 2022 and request authors to consider both actual and perceived competing interests. Please review the policy https://www.embopress.org/competing-interests and update your competing interests if necessary.

15) Every published paper now includes a 'Synopsis' to further enhance discoverability. Synopses are displayed on the journal webpage and are freely accessible to all readers. They include a short stand first (maximum of 300 characters, including space) as well as 2-5 one-sentences bullet points that summarizes the paper. Please write the bullet points to summarize the key NEW findings. They should be designed to be complementary to the abstract - i.e. not repeat the same text. We encourage inclusion of key acronyms and quantitative information (maximum of 30 words / bullet point). Please use the passive voice. Please attach these in a separate file or send them by email, we will incorporate them accordingly.

Please also suggest a visual abstract to illustrate your article as a PNG file 550 px wide x 300-600 px high. A cropped portion of this image will serve as thumbnail for the table of content on our webpage.

16) As part of the EMBO Publications transparent editorial process initiative (see our Editorial at http://embomolmed.embopress.org/content/2/9/329), EMBO Molecular Medicine will publish online a Review Process File (RPF) to accompany accepted manuscripts.
In the event of acceptance, this file will be published in conjunction with your paper and will include the anonymous referee reports, your point-by-point response and all pertinent correspondence relating to the manuscript. Let us know whether you agree with the publication of the RPF and as here, if you want to remove or not any figures from it prior to publication.
Please note that the Authors checklist will be published at the end of the RPF.

EMBO Molecular Medicine has a "scooping protection" policy, whereby similar findings that are published by others during

review or revision are not a criterion for rejection. Should you decide to submit a revised version, I do ask that you get in touch after three months if you have not completed it, to update us on the status.

I look forward to receiving your revised manuscript.

Yours sincerely,

Lise Roth

Lise Roth, PhD
Senior Editor
EMBO Molecular Medicine

***** Reviewer's comments *****

Referee #1 (Comments on Novelty/Model System for Author):

I have concerns regarding the rigor of the study. Authors need to provide additional measurements of lung fibrosis including Col1a1, Col1a2, Alpha SMA and fibronectin gene expression in additional to hydroxyproline levels and Ashcroft score. Also, it seems that the Terc-/- mice have features of emphysema but they are not really discussing the degree and severity of emphysema in the paper and how that can potentially confound their findings.

Referee #1 (Remarks for Author):

This study investigates the role of telomere dysfunction-induced DNA damage response (tDDR) in idiopathic pulmonary fibrosis (IPF) using a genetic mouse model (Terc-/-) that mirrors key features of human IPF, including telomere shortening, cellular senescence, immune activation, and lung fibrosis. Importantly, the model also replicates transcriptomic changes seen in both aged mice and IPF patients. To probe the pathological contribution of tDDR, researchers employed telomeric antisense oligonucleotides (tASOs) targeting telomeric non-coding RNAs. The findings suggest that treatment with tASOs in both young and adult mice slowed fibrosis progression and reversed established lung fibrosis, demonstrating disease-modifying potential. The study also showed that tASO treatment reduced γH2AX, a marker of DNA damage, through both cell-autonomous and immune-mediated mechanisms. Notably, the treatment normalized gene expression profiles shared by telomerase-deficient mice and IPF patients, suggesting that tDDR-not telomere length itself-is the key driver of pathology. Furthermore, the therapeutic effects were consistent across models of telomere dysfunction with different genetic causes, supporting the broad applicability of tDDR inhibition. Given that telomere dysfunction also increases with normal aging, this approach may extend beyond genetic IPF to age-related fibrotic and degenerative conditions.
While this manuscript provides a potential therapeutic target for the treatment of IPF, I have some concern regarding the rigor of the research and some of the findings presented. For example, it seems like the Terc -/- mice showed signs of emphysema but the extent of emphysema is not described in the manuscript or the implications of the degree of emphysema as a confounding factor. The authors need to present whole lung figures rather than selected sections.
Please see my point-by-point comments below
Page 7, lines 2-7. Please provide validation of H2AX and 53BP1 in addition to staining methods. Either by qRT-PCR or western blot. When representing tissue samples, please add pictures from whole lungs. Authors used a pathological score but do not provide a reference score. Please use Ashcroft score instead, a well-validated score of lung fibrosis.
Page 8, lines 14-17. In all figures that represent tissue fibrosis or response to anti-teloG or anti-teloC treatment, authors need to provide objective measurements of lung fibrosis such as tissue Col1a1, Col1a2, alpha SMA and fibronectin gene expression as well as hydroxyproline levels in tissue lysates. Also whole lungs should be shown. Not just selected areas. I would also recommend treating a the Terc-/- mice with low dose bleomycin (1 u/kg) and assess the treatment effect with anti-teloG or anti-teloC. This would be more representative of the human pathology as it would represent alveolar epithelial cell injury in the background of genetic mutation. The effect of anti-teloG and anti-teloC should be more pronounced.
Page 8, lines 21-22, I would recommend using "genetic model of murine pulmonary fibrosis" instead of genetic mouse model of IPF. IPF is a far more complex disease where the genetic component is one of the many components of the disease.
Page 8, line 24. Please add whole lung pictures and other objective measurements of lung fibrosis such as Col1a1, Col1a2, alpha SMA and fibronectin gene expression as well as hydroxyproline levels in tissue lysates. Please also use the Ashcroft score.
Please quantify the degree of emphysema observed in the Terc-/- mice and changes in the degree of emphysema after anti-

teloG and anti-teloC treatment.

Referee #2 (Comments on Novelty/Model System for Author):

Please see my review. While the study has potentially high impact, several important limitations remain due to insufficient characterization of the tASO mechanism of action.

Referee #2 (Remarks for Author):

The authors previously reported that telomere dysfunction triggers the transcription of DDR dependent telomeric RNAs (tDDRNAs) from both DNA strands and that their targeted inhibition using sequence-specific antisense oligonucleotides (tASOs) effectively suppresses the telomere-associated DDR (Rosiello et al 2017, Aguado et al 2019). Here, the authors utilized third-generation Terc$^{-/-}$ mice (G3 Terc-/-) as a genetic model of idiopathic pulmonary fibrosis (IPF), characterized by elevated levels of DNA damage, inflammation, and parenchymal fibrosis in the lungs. They show that systemic treatment with tASOs (anti-teloG and anti-teloC) reduced DNA damage, fibrosis and lung pathotogy. In particular, the authors demonstrate that tASO treatment reduces DNA damage in alveolar type II cells previously implicated in telomere dysfunction-driven lung fibrosis. They further report that telomere dysfunction in G3 Terc$^{-/-}$ mice mirrors the transcriptional alterations seen in naturally aged lungs and that treatment with tASOs reverses age-related gene expression changes in G3 Terc$^{-/-}$ lungs. In particular, tASO treatment attenuates transcriptional signatures associated with cellular senescence, inflammation, and complement pathways. Finally, Multivariate analysis indicates that tASO treatment counteracts molecular and pathological features in the lung suggesting that lung pathology is more closely linked to telomeric damage response than to telomere length itself.

This is a well-written manuscript that presents compelling evidence that systemic tASO treatment alleviates age-related lung dysfunction in G3 Terc$^{-/-}$ mice, a genetic model that recapitulates key features of pulmonary fibrosis. These findings support tASOs as a promising therapeutic strategy for idiopathic pulmonary fibrosis (IPF). However, to strengthen the conclusions and broaden the impact of the manuscript, further characterization of tASO effects is required. Below are several suggestions aimed at improving the impact of the study:

1. tASOs are designed to target telomeric DDRNAs (tDDRNAs), which have been proposed to mediate DDR activation at dysfunctional telomeres. The authors should demonstrate that tDDRNAs are indeed produced in the lungs of G3 Terc$^{-/-}$ mice to confirm the target presence and relevance in this model.

2. Throughout the manuscript, tDDR inhibition is inferred from reductions in γH2AX and 53BP1 foci. However, these are primarily markers of double-strand breaks, not direct indicators of DDR signaling activation. To substantiate the claim that tASOs inhibit tDDR, phospho-specific antibodies against DDR signaling proteins such as ATM (pATM Ser1981) should be included.

3. The authors should assess whether tASO treatment specifically reduces DNA damage at telomeres of G3 Terc$^{-/-}$ mice . This could be achieved by analyzing telomere dysfunction-induced foci (TIFs in treated and untreated G3 Terc$^{-/-}$ lungs.

4. Given the central role of critically short telomeres in telomere-driven lung dysfunction (PMID: 38424230), telomere length should be measured in the lung parenchyma of WT, G3 Terc$^{-/-}$, and tASO-treated G3 Terc$^{-/-}$ mice. Southern blot or, preferably, TeSLA that would provide informative data on the shortest telomeres.

5. Suppression of senescence and inflammation is currently inferred from transcriptomic signatures. These findings would be significantly strengthened by histological or immunohistochemical evidence, such as SA-β-Gal staining, or immunostaining for senescence markers like p21.

6. The authors should provide the full list of differentially expressed genes (DEGs) corresponding to Figures 3A, 3B, and 3C in an Excel file. Additionally, volcano plots highlighting the top 20 upregulated and downregulated genes would improve data. In Figure 4, the senescence and inflammation-related genes displayed in the heatmaps should be clearly indicated.

7. Bulk RNA-seq reflects average gene expression across multiple cell types. Could the author perform cell-type deconvolution using available lung single-cell RNA-seq references to identify which cell populations contribute most to the observed DEGs.

8. The manuscript shows reduced γH2AX and 53BP1 foci in ATII cells following tASO treatment. It would be very informative to assess whether this reduction is specific to ATII cells or also occurs in other lung cell types, such as endothelial or fibroblast populations.

Referee #3 (Remarks for Author):

The manuscript by Rossiello et al., entitled "Telomeric DNA damage response as a target for the treatment of Idiopathic Pulmonary Fibrosis" presents an interesting and well-structured study. The authors demonstrate that activation of the telomeric DNA damage response (tDDR) can lead to the spontaneous development of lung fibrosis and associated pathologies in mice, even in the absence of external/additional injury (e.g., bleomycin-induced lung fibrosis). Using a mouse model lacking the RNA component of telomerase (Terc), they show that Terc-knockout mice develop fibrosis as early as 3 months of age, a phenotype that persists into adulthood at 12 and 20 months. Furthermore, the authors validate the antifibrotic efficacy of two antisense oligonucleotides (ASOs) in both adult and aged mice. The RNA-seq data are thoroughly analyzed, and the comparison of mice data with human idiopathic pulmonary fibrosis (IPF) datasets reveals notable similarities.

Overall, the manuscript is well-written and the figures are compelling. However, important key RNA-seq datasets and immunofluorescence (IF) quantifications that would strengthen the authors' conclusions are currently missing.

Major Comments:
Figure 1:
The authors have validated the effect of Terc-knockout (Terc-KO) in inducing fibrosis in both young and aged mice. However, it is essential to identify the specific lung cell populations affected by Terc-KO.
• Determine whether the frequency of alveolar epithelial type 2 (AT2) cells changes in Terc-KO mice.
• Assess whether Terc-KO mice exhibit elevated TGF-β signaling pathway activity (e.g., IF stains for phospho-Smad2/3).

Figure 2 and EV2 Results Section:
The manuscript refers to signs of "emphysema". This term may be inappropriate in this context. Decreased fibrosis and restoration of alveolar architecture do not necessarily indicate emphysema, particularly without a significant increase in airspace size/number between wild-type controls and ASO-treated mice. Reconsider the terminology used.

Figure 2 and EV2:
The data demonstrate attenuation of fibrosis and restoration of airspaces in ASO-treated mice, but information on the cellular outcomes in fibrotic lungs is missing.
• Determine whether activated myofibroblasts (aMYFs) are undergoing apoptosis, as this is one of the key mechanisms in fibrosis resolution.
• Include IF staining and quantification of aMYF markers (α-SMA, Col1a1).

Figures 3 and 4:
Since 3-month-old Terc-KO mice lungs show fibrosis, include a comparison between 3-month-old Terc-KO and age-matched wild-type mice (in panels A or a separate EV figure) to assess aging and senescence signatures as early as 3 months of age.

Figure 4:
In addition to the current GSEA plots (e.g., SenMayo, Universal senescence signatures, inflammatory response), include GSEA plots for fibrotic signature (aMYF markers), AT2/AT1 cell signatures, and $Krt8^{+}$ (AT2-AT1) transitional cell's signature.

Figures 5 and EV4:
AT2 cell frequencies appear unchanged across the experimental groups. However, to better assess potential enhancement of AT2-to-AT1 differentiation, it is important to include analyses of proliferation status, as well as quantification of AT2 cells, $Krt8^{+}$ transitional cells (AT2-AT1 intermediates) and AT1 cells across conditions.

Figure 6:
The PCA plot shows that tDDR-treated samples cluster closer to wild-type mice but do not fully correlate or align. Include RNA-seq data from 3-month-old Terc-KO lungs to determine if treated aged mice exhibit similar transcriptomic profiles.

Figure 8:
Include a comparison between young Terc-KO and wild-type mice in panel A. Add GSEA plots showing TGF-β signature changes for additional mechanistic insight.

Minor Comments:
• Indicate the number of mice used in each experiment.
• Separate and align the in vivo ASO treatment schematics with the relevant data. Figure 2 uses 12-month-old mice, while Figure EV2 uses 5-month-old mice, schematics should reflect this distinction.
• Reconsider the use of the term emphysema in the manuscript, as previously noted.
• Expanding the discussion on the Terc-knockout mouse model would provide valuable insights. Despite developing fibrosis by 3 months, the mice survive up to 20 months, unlike human IPF patients who typically survive 4-5 years post-diagnosis. Discuss possible explanations for this discrepancy.

***** Reviewer's comments *****

Referee #1 (Comments on Novelty/Model System for Author):

I have concerns regarding the rigor of the study. Authors need to provide additional measurements of lung fibrosis including Col1a1, Col1a2, Alpha SMA and fibronectin gene expression in additional to hydroxyproline levels and Ashcroft score. Also, it seems that the Terc-/- mice have features of emphysema but they are not really discussing the degree and severity of emphysema in the paper and how that can potentially confound their findings.

We thank Reviewer 1 for this comment, which helped us to improve the rigor of the study. Following these indications, we have now expanded the assessment of lung fibrosis by incorporating multiple complementary readouts. Specifically, we now include histological evaluation using the Ashcroft score, biochemical quantification of collagen content by hydroxyproline assay and mRNA and protein expression analyses by RT-qPCR and immunohistochemistry staining of key fibrotic markers (Col1a1, Col1a2, α-SMA, and Fn1). Together, these additional measurements (see detailed responses below) provide a comprehensive, robust and objective evaluation of lung fibrosis and overall strengthen the conclusions of the manuscript.
In regards to emphysema, while Terc⁻/⁻ mice may display emphysema-like features, the extent of emphysema was not quantified in the present study, since a rigorous assessment requires standardized lung inflation and fixation and systematic whole-lung stereological sampling, which were not included in the study design. For this reason, we did not discuss the degree of emphysema and we limited our analysis to descriptive morphological features. We have now accordingly revised the manuscript to tone down the terminology and now consistently refer to these alterations as “emphysema-like” features limiting our observations to the objective alteration in air spaces number.

Referee #1 (Remarks for Author):

This study investigates the role of telomere dysfunction-induced DNA damage response (tDDR) in idiopathic pulmonary fibrosis (IPF) using a genetic mouse model (Terc-/-) that mirrors key features of human IPF, including telomere shortening, cellular senescence, immune activation, and lung fibrosis. Importantly, the model also replicates transcriptomic changes seen in both aged mice and IPF patients. To probe the pathological contribution of tDDR, researchers employed telomeric antisense oligonucleotides (tASOs) targeting telomeric non-coding RNAs. The findings suggest that treatment with tASOs in both young and adult mice slowed fibrosis progression and reversed established lung fibrosis, demonstrating disease-modifying potential. The study also showed that tASO treatment reduced γH2AX, a marker of DNA damage, through both cell-autonomous and immune-mediated mechanisms. Notably, the treatment normalized gene expression profiles shared by telomerase-deficient mice and IPF patients, suggesting that tDDR-not telomere length itself-is the key driver of pathology. Furthermore, the therapeutic effects were consistent across models of telomere dysfunction with different genetic causes, supporting the broad applicability of tDDR inhibition. Given that telomere dysfunction also

increases with normal aging, this approach may extend beyond genetic IPF to age-related fibrotic and degenerative conditions.
While this manuscript provides a potential therapeutic target for the treatment of IPF, I have some concern regarding the rigor of the research and some of the findings presented. For example, it seems like the Terc -/- mice showed signs of emphysema but the extent of emphysema is not described in the manuscript or the implications of the degree of emphysema as a confounding factor. The authors need to present whole lung figures rather than selected sections.

We thank Reviewer 1 for the insightful evaluation of our work and for highlighting important points related to experimental rigor. We have now revised the manuscript to address all concerns. We agree with the Reviewer's expert interpretation that Terc⁻/⁻ mice may display features compatible with emphysema. However, the available tissue sampling did not allow a robust quantitative assessment of emphysema degree or severity, which requires standardized lung inflation and fixation as well as systematic whole-lung stereological analysis. For this reason, we did not discuss the extent or severity of emphysema in the manuscript and revised the wording to "emphysema-like morphological features", thereby minimizing the risk of overinterpretation or confounding of the main findings. We are also providing whole lung figures, as descrided below.

Please see my point-by-point comments below
Page 7, lines 2-7. Please provide validation of gH2AX and 53BP1 in addition to staining methods. Either by qRT-PCR or western blot.

We thank the reviewer for this comment. We have extensive experience in assessing DNA damage response (DDR) activation by immunostaining of canonical DDR markers, including γH2AX and 53BP1, in both human and mouse cells and tissues (Fumagalli et al, Nat Cell Biol, 2012; Francia et al, Nature, 2012; Rossiello et al, Nat Comm, 2017; Michelini et al, Nat Cell Biol, 2017; Aguado et al, Nat Comm, 2019; Gioia et al Nat Cell Biol, 2023; Sepe et al, EMBO J, 2025). In particular, we have recently validated the specificity of γH2AX and 53BP1 immunostaining in mouse lung tissues in our publication (Gioia et al, Nat Cell Biol, 2023): in that study, using the same antibodies and identical staining conditions as those employed here, we validated γH2AX and 53BP1 staining in untreated and X-ray-irradiated mouse lung sections. This approach demonstrated a strong and specific nuclear signal induced exclusively in irradiated samples, confirming antibody specificity and staining reliability (Extended Data Fig. 7 C,D, also shown below).

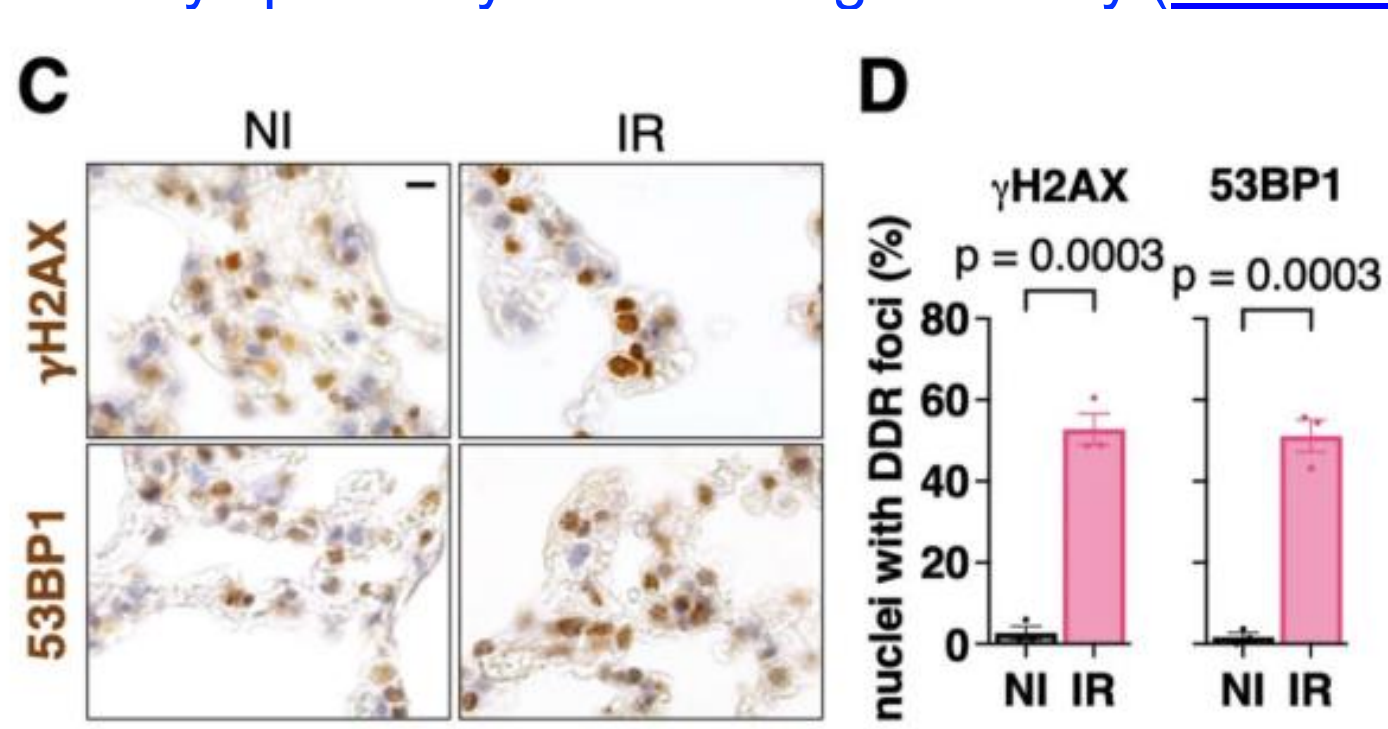

Importantly, qRT-PCR analysis is not informative for γH2AX, as its signal reflects a post-translational phosphorylation event of the histone H2AX rather than changes in transcript abundance. Similarly, 53BP1 foci formation results from the redistribution of pre-existing 53BP1 protein to sites of DNA damage, rather than increased mRNA or protein expression levels. Therefore, immunostaining-based detection represents the most appropriate and widely accepted method to assess DDR activation in tissue sections.
It is also worth mentioninhg here that, we deeply characterized DDR activation by the inclusion of additional DDR markers tested by immunohistochemical stainings, namely pATM, the autophosphorylated and thus activated form of ATM, and pKAP1, a modification specifically mediated by ATM (Goodarzi et al, Nat Struc Mol Biol, 2011). Treatment with tASOs resulted in a robust reduction of both pATM and pKAP1, which were increased in Terc-/- mice compared with wild type controls (Fig. 2d-e, also shown below), aligning with and strengthening conclusions based on γH2AX and 53BP1 studies.

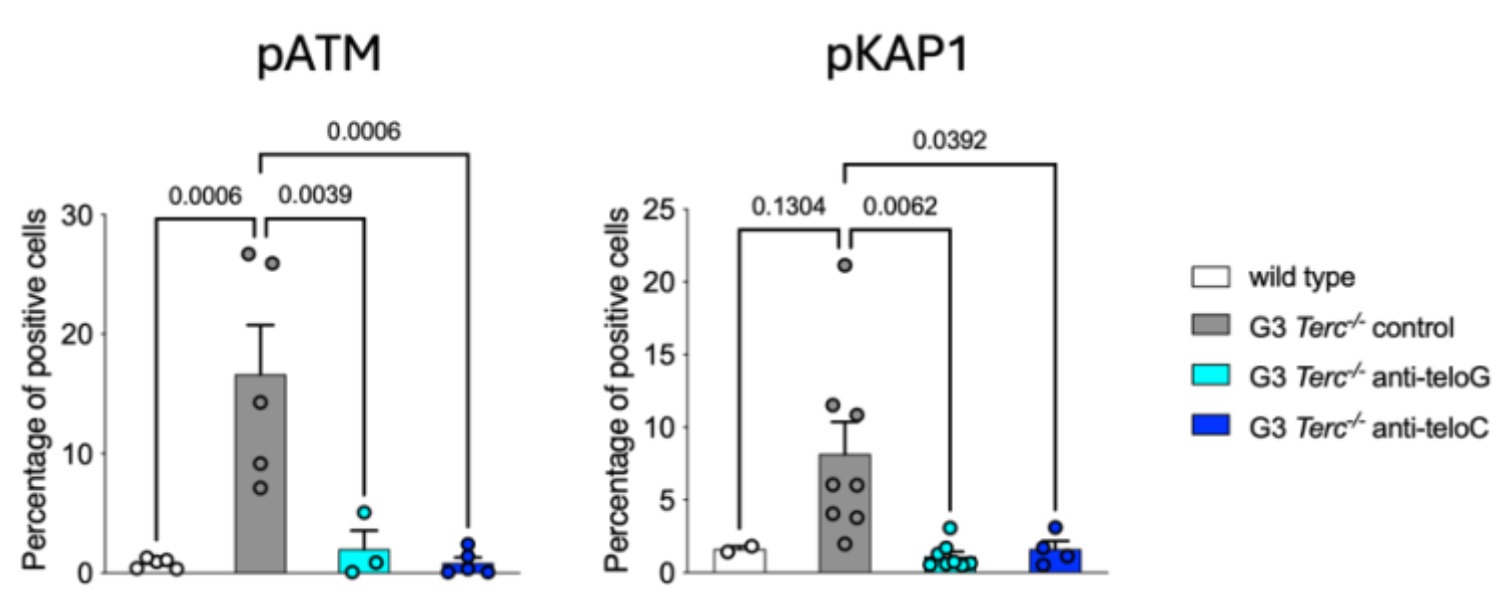

When representing tissue samples, please add pictures from whole lungs. Authors used a pathological score but do not provide a reference score. Please use Ashcroft score instead, a well-validated score of lung fibrosis.

We thank the reviewer for this helpful suggestion. As requested, we have now included representative images of whole lungs to provide an overview of the morphological changes in the lung parenchyma (see Fig. EV 1d, EV 1g, EV 3b, EV 3e). We have prioritized full-section images for H&E and Masson's trichrome stainings, as we consider these the most informative, due to space limitations. However, we will be happy to provide whole-lung images for all other stainings, including high-resolution versions, should the reviewer find them helpful – we seek Editorial advice for uploading large and high resolution (heavy) images.
Regarding the histopathological evaluation, as detailed in the Methods section, we employed and adapted a predefined semiquantitative scoring system based on established morphological criteria similar to that previously applied in our published work aimed at evaluating lung pathology (Sangaletti et al, Am J Pathol, 2011). Specifically, the following parameters were assessed: inter-alveolar septal thickening, increased interstitial cellularity, interstitial inflammatory infiltration, intra-alveolar inflammatory infiltration, and erythrocyte extravasation. All parameters were evaluated in a blinded manner by a chartered pathologist and scored according to both severity (0 = normal; 1 = slight; 2 = moderate; 3 = marked) and extension (0 = absent; 1 = focal; 2 = multifocal;

3 = diffuse). For each sample, an overall morphological/pathological score was calculated as the product of severity and extension. This approach allowed us to capture both the intensity and distribution of histopathological alterations.
Regarding the Reviewer's suggestion to adopt the Ashcroft score, we appreciated this recommendation and we have now performed fibrosis grading using the Ashcroft scoring system (Hübner et al, BioTechniques, 2008) to complement the pathological score originally reported. The results of the Ashcroft scoring, now shown in Fig. 1c and 3a, are fully consistent with those obtained with the pathological score previously employed.

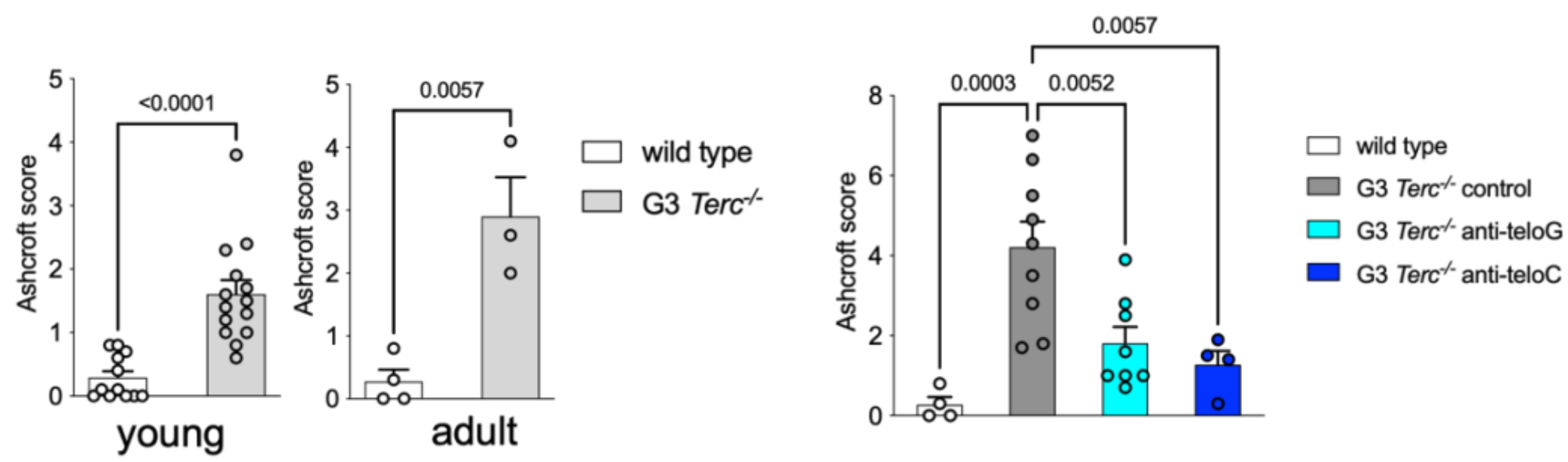

Page 8, lines 14-17. In all figures that represent tissue fibrosis or response to anti-teloG or anti-teloC treatment, authors need to provide objective measurements of lung fibrosis such as tissue Col1a1, Col1a2, alpha SMA and fibronectin gene expression as well as hydroxyproline levels in tissue lysates.

We thank the Reviewer for this comment. The original analyses were designed to provide quantitative and pathologically relevant assessments of lung fibrosis. In this study, lung fibrosis was assessed using Masson's trichrome staining. This is a well-established (Islam and Kumar, Methods Mol Biol, 2026) and widely employed histochemical assay for the detection and quantification of collagen deposition and fibrotic remodeling in lung tissue, specifically staining fibrillar collagens (types I and III) and also visualizing reticulin fibers. This technique allows direct visualization of fibrotic areas within their anatomical context and is therefore highly relevant from a pathological standpoint.
Importantly, Masson's trichrome staining was not evaluated qualitatively but was subjected to rigorous and unbiased quantification. Indeed, five non-overlapping and unbiasedly chosen fields were analyzed per lung section, selected according to predefined criteria to avoid sampling bias. Fibrosis was quantified by measuring collagen density, rather than total staining signal, providing a normalized and objective readout of fibrotic burden. Image analysis and signal quantification was performed using a dedicated and validated software (HALO, Indica Labs). Finally, each data point shown in the graphs represents one individual mouse. Therefore, we are confident that such measures are rigorous, robust, quantitative and conclusions drawn are thus pathologically relevant.

Text removed because it referred to a figure intended for reviewers only and to unpublished findings.

Figure for reviewers removed

Notably, while an entire lobe is usually employed for such measures, this was not possible in our set of samples and thus only a portion of it was used, and normalization was based on tissue weight. This likely caused and underestimation of the differences observed since fibrotic tissues have a known higher weight. Therefore we leave to this Reviewer's and to our Editor's discretion to included these results or leave them for Reviewers' only as we would be prone to.

Text removed because it referred to a figure intended for reviewers only and to unpublished findings.

Figure for reviewers removed

This result should be interpreted in light of the genetic model of lung fibrosis we have employed, which is fundamentally different from others, such as the bleomycin-induced fibrosis model, which is often used (Tashiro et al, Front Med, 2017; Moore et al, Am J Respir Cell Mol Biol, 2013), and equally often criticized (Moeller et al Int J Biochem Cell Biol, 2008; Brazee et al, Semin Respir Crit Care Med, 2025), and may have prompted such requests. Indeed, bleomycin instillation induces an acute, injury-driven fibrotic response in the mouse lung characterized by rapid and strong transcriptional activation of inflammation and profibrotic genes, which, however, are spontaneously reversed with time. This is opposite to the IPF condition in humans that is slow, progressive and irreversible. Our genetic model is based on a germline alteration, akin to that most common (15%) observed in familiar IPF patients (Alonso-Gonzalez, Front Med, 2023) thus more accurately modeling the chronic and progressive fibrotic process observed in humans.

In this context, histological collagen accumulation (e.g. as evaluated by Masson's trichrome) reflects integrated, long-term extracellular matrix (ECM) deposition and remodeling that may not be fully recapitulated by snapshot changes of profibrotic genes studied in bulk mRNA levels by RT-qPCR.

Consistent with this, transcriptional upregulation of classical fibrotic markers such as Col1a1, Col1a2, and Acta2 has not been consistently observed and reported in bulk lung tissue from human IPF patients. When observed, increased Col1a1 and other fibrotic markers expression have been detected primarily in bronchoalveolar lavage (BAL) cells (Tsitoura et al, Front Immunol, 2021) or within specific lung cell populations identified by single-cell RNA sequencing (Tsukui et al, Nat Comm, 2020), rather than at the whole-tissue level. To verify this, we examined ourselves the human RNA-seq datasets also used in our manuscript and shown in Fig. 9, which compare lung tissue from IPF patients with healthy controls. We noticed that the differences in expression of Col1a1, Col1a2, Acta2, and Fn1 were small, with only a single dataset showing a statistically significant increase in Acta2 and even in this case the change was not reproduced in a second independent dataset (see Figure below).

Figure for reviewers removed

Of course, we do not mean to imply that collagen accumulation or extracellular matrix deposition does not occur in IPF. Rather, our point is that this increase does not uniformly translate into a strong and robust transcriptional induction analysed in bulk lung tissue.

Also whole lungs should be shown. Not just selected areas.

As mentioned above, we have now included representative images of whole lungs for H&E and Masson's trichrome staining (see Fig. EV 1d, EV 1g, EV 3b, EV 3e). However, we would be happy to provide whole-lung images for all other stainings, including high-resolution versions, should the reviewer find them helpful.

I would also recommend treating the Terc-/- mice with low dose bleomycin (1 u/kg) and assess the treatment effect with anti-teloG or anti-teloC. This would be more representative of the human pathology as it would represent alveolar epithelial cell injury in the background of genetic mutation. The effect of anti-teloG and anti-teloC should be more pronounced.

Although we agree with this Reviewer that combining genetic susceptibility with bleomycin-induced injury could be interesting, as argued above, bleomycin triggers an

acute and largely reversible inflammatory and fibrotic response which does not reflect the chronic, progressive, and irreversible fibrosis characteristic of human IPF. In contrast, the G3 $Terc^{-/-}$ model used here recapitulates age-dependent, slow-onset telomere-driven lung fibrosis, observed in humans.

In addition, as this Reviewer will agree, implementing a bleomycin challenge in this genetic model would require establishing of a new experimental system, including obtaining new ministerial ethical authorization for animal experimentation, as mandated by national regulations, as well as optimization dosing, timing, and bleomycin intratracheal instillation, which would significantly delay resubmission.

In agreement with our Editor, we concluded that establishing an additional model is beyond the scope of the current manuscript.

Page 8, lines 21-22, I would recommend using "genetic model of murine pulmonary fibrosis" instead of genetic mouse model of IPF. IPF is a far more complex disease where the genetic component is one of the many components of the disease.

We thank the Reviewer for this suggestion. The wording in the manuscript has now been updated to “genetic model of murine pulmonary fibrosis” to accurately reflect the scope of the model and avoid overstatements.

Page 8, line 24. Please add whole lung pictures and other objective measurements of lung fibrosis such as Col1a1, Col1a2, alpha SMA and fibronectin gene expression as well as hydroxyproline levels in tissue lysates. Please also use the Ashcroft score.

As mentioned above, we have now included representative images of whole lungs for H&E and Masson’s trichrome staining (see Fig. EV 1d, EV 1g, EV 3b, EV 3e). However, we would be happy to provide whole-lung images for all other stainings, including high-resolution versions, should the reviewer find them helpful.

As already discussed above, our evaluation of lung pathology relies on quantitative and objective assessments.

Text removed because it referred to a figure intended for reviewers only and to unpublished findings.

These findings further support the notion that, in our constitutive, age-dependent, telomere-driven fibrosis model, fibrotic remodeling may occur without marked transcriptional changes detectable in bulk lung tissue, as discussed above.

Figure for reviewers removed

Since the expression levels of individual fibrotic genes do not seem to show significant changes when analysed at the single-gene level, we performed gene set enrichment analyses (GSEA) of our RNA-seq data to extract a more robust pathway-based transcriptomic assessment. Interestingly, these analyses demonstrated a significant enrichment of the TGF-β signaling pathway, a key regulator of fibrotic remodeling, in G3 Terc$^{-/-}$ lungs compared with wild-type controls. Importantly, tASO treatment reversed this enrichment, shifting TGF-β pathway activity toward wild-type levels (Fig. EV 5a, also show below).

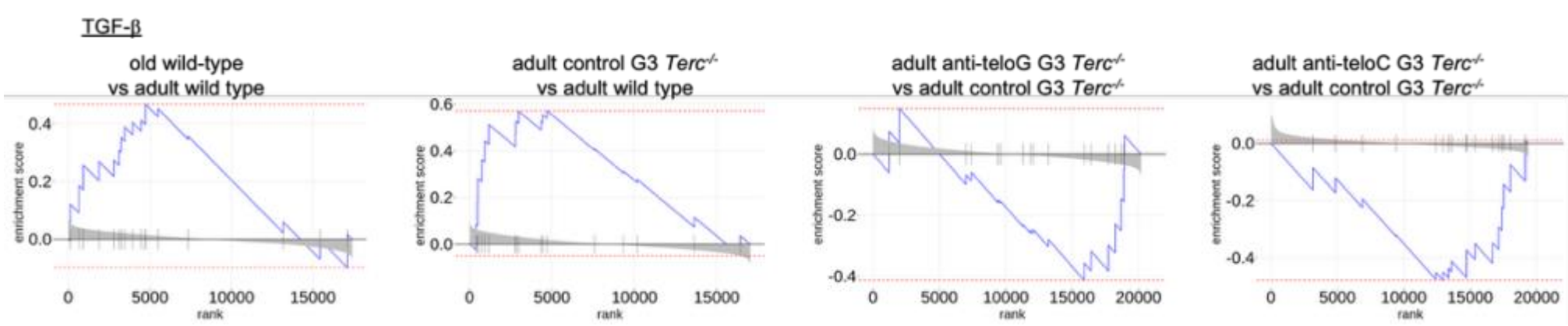

These results suggest that, while some single-gene differences may be subtle, their concerted regulation across the pathway results in a more robust and statistically significant shift in TGF-β signaling activity, pointing to pathway analysis as a more sensitive and biologically informative readout of fibrotic remodeling than individual gene-based comparisons.

When we quantified the level of α-SMA by immunohistochemistry in adult mice, we observed a trend toward an increase in Terc-/- mice, which was significantly reduced by tASOs treatment (Fig. 3c, also shown below).

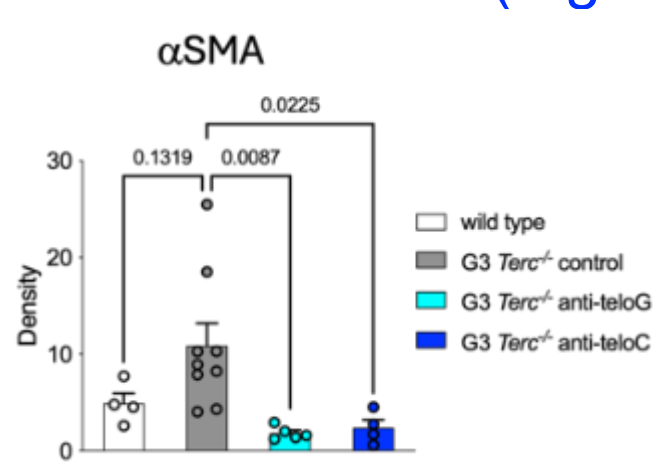

Similarly, quantification of Col1a1 signal by immunohistochemistry in adult mice showed a significant decrease in tASOs-treated animals (Fig. 3d, also shown below). This reduction is consistent with an attenuation of fibrotic remodeling, as Col1a1 is a major component of extracellular matrix deposition and a well-established marker of activated fibroblasts.

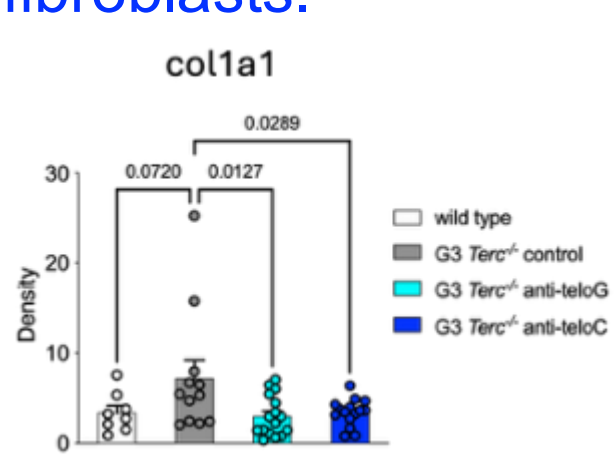

Importantly, we also performed Ashcroft scoring to provide an additional quantitative assessment of lung fibrosis. As shown in the revised manuscript (Fig. 3a, also shown

below), Ashcroft scores are increased in G3 Terc-/- mice and are significantly reduced by both tASOs treatment, consistent with the other histopathological features.

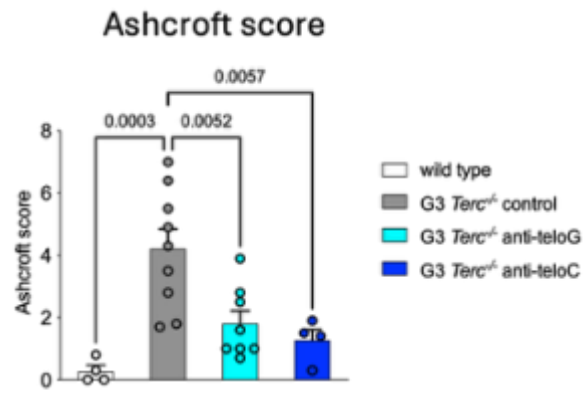

Taken together, these data consistently show that telomere-driven fibrotic changes in G3 Terc⁻/⁻ lungs are modest at the single-gene level but robust and significant at the pathway and histological levels, and that they are significantly ameliorated by tASOs treatment.

Please quantify the degree of emphysema observed in the Terc-/- mice and changes in the degree of emphysema after anti-teloG and anti-teloC treatment.

We thank the Reviewer and fully agree that telomerase-deficient mice can show emphysema-like features, which would possibly represent a further relevant phenotype. However, we also acknowledge that robust emphysema quantification requires standardized inflation/fixation and systematic whole-lung stereological sampling, which were not part of the present study design. This is why we have not extended the analyses of such features. Accordingly, we have revised the manuscript to refer to these alterations as “emphysema-like” features rather than emphysema, since the phenotype was measured by altered air space number without formal stereological quantification of disease severity.

In summary, we believe that the Reviewer’s concern regarding the objective quantification of fibrosis has been fully addressed. We have demonstrated the accuracy and pathological relevance of our Masson’s trichrome measurements, supported by Ashcroft scoring, α-SMA and Col1a1 immunohistochemistry, hydroxyproline measurements, and pathway analyses, all of which consistently indicate increased fibrosis in G3 Terc-/- mice and significant reduction upon tASOs treatment. The changes in individual Col1a1, Col1a2, Acta2, and Fn1 mRNAs may not be expected in this genetic telomere-driven model characterized by slow but naturally irreversible fibrotic remodeling similar to the human IPF pathology.

Referee #2 (Comments on Novelty/Model System for Author):

Please see my review. While the study has potentially high impact, several important limitations remain due to insufficient characterization of the tASO mechanism of action.

We thank Reviewer 2 for recognizing the potential impact of our study. We acknowledge the importance of thoroughly characterizing the mechanism of action of tASOs. In response, we have expanded our analyses to provide additional evidence supporting the mechanism of tASO action. These additions address the reviewer’s concerns and further

strengthen the mechanistic rigor of the study. Detailed point-by-point responses are provided below.

Referee #2 (Remarks for Author):

The authors previously reported that telomere dysfunction triggers the transcription of DDR dependent telomeric RNAs (tDDRNAs) from both DNA strands and that their targeted inhibition using sequence-specific antisense oligonucleotides (tASOs) effectively suppresses the telomere-associated DDR (Rosiello et al 2017, Aguado et al 2019). Here, the authors utilized third-generation Terc⁻/⁻ mice (G3 Terc-/-) as a genetic model of idiopathic pulmonary fibrosis (IPF), characterized by elevated levels of DNA damage, inflammation, and parenchymal fibrosis in the lungs. They show that systemic treatment with tASOs (anti-teloG and anti-teloC) reduced DNA damage, fibrosis and lung pathotogy. In particular, the authors demonstrate that tASO treatment reduces DNA damage in alveolar type II cells previously implicated in telomere dysfunction-driven lung fibrosis. They further report that telomere dysfunction in G3 Terc⁻/⁻ mice mirrors the transcriptional alterations seen in naturally aged lungs and that treatment with tASOs reverses age-related gene expression changes in G3 Terc⁻/⁻ lungs. In particular, tASO treatment attenuates transcriptional signatures associated with cellular senescence, inflammation, and complement pathways. Finally, Multivariate analysis indicates that tASO treatment counteracts molecular and pathological features in the lung suggesting that lung pathology is more closely linked to telomeric damage response than to telomere length itself.
This is a well-written manuscript that presents compelling evidence that systemic tASO treatment alleviates age-related lung dysfunction in G3 Terc⁻/⁻ mice, a genetic model that recapitulates key features of pulmonary fibrosis. These findings support tASOs as a promising therapeutic strategy for idiopathic pulmonary fibrosis (IPF).
However, to strengthen the conclusions and broaden the impact of the manuscript, further characterization of tASO effects is required.

We thank the Reviewer for highlighting the importance of further characterizing the effects of tASOs treatment. In response, we have conducted additional analyses to provide more detailed insights into the molecular and cellular consequences of tASOs administration. These new data complement the original findings and strengthen our mechanistic understanding of how tASOs mitigate telomere dysfunction-driven DNA damage, inflammation, and fibrosis. Point-by-point responses addressing each specific suggestion are provided below.

Below are several suggestions aimed at improving the impact of the study:

1. tASOs are designed to target telomeric DDRNAs (tDDRNAs), which have been proposed to mediate DDR activation at dysfunctional telomeres. The authors should demonstrate that tDDRNAs are indeed produced in the lungs of G3 Terc⁻/⁻ mice to confirm the target presence and relevance in this model.

Telomeric damage-induced non-coding RNAs (tdincRNAs) have been extensively characterized in multiple models of telomere dysfunction (Rossiello et al, Nat Comm, 2017; Aguado et al, Nat Comm, 2019; Sepe et al, EMBO J, 2025), including mouse embryonic fibroblasts (MEFs) upon inducible telomere deprotection, human fibroblasts expressing progerin (as a model of Hutchinson-Gilford Progeria Syndrome (HGPS), an accelerated aging disease), skin samples from HGPS patients, and primary neurons treated with Aβ42 oligomers (as a model of Alzheimer's disease). Our conclusions are based on orthogonal independent approaches including RT-qPCR, RNA FISH, and RNA-seq to demonstrate the presence and functional relevance of tdincRNAs in cells and tissues across species.
In this manuscript, to address the Reviewer's request in the context of our model, we are now including measurements of such transcripts in mouse lungs by RT-qPCR. We observed an increase of both C- and G-rich RNA strands in lungs from G3 Terc-/- mice compared with age-matched wild-type controls (Fig. EV 1c, also shown below), confirming the presence of the tASOs targets also in this model and supporting the rationale for tASOs use.

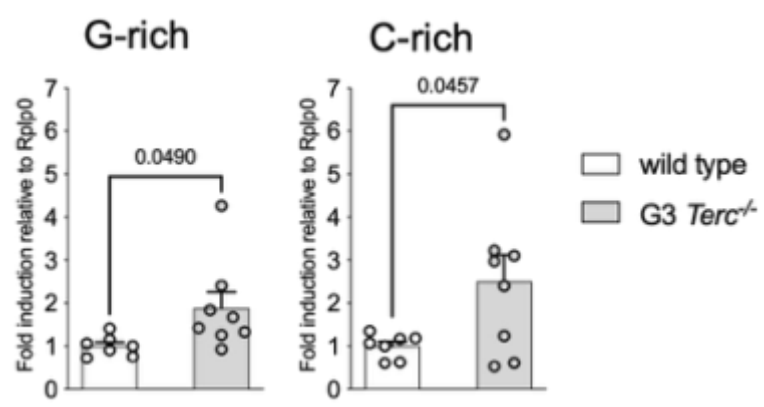

2. Throughout the manuscript, tDDR inhibition is inferred from reductions in γH2AX and 53BP1 foci. However, these are primarily markers of double-strand breaks, not direct indicators of DDR signaling activation. To substantiate the claim that tASOs inhibit tDDR, phospho-specific antibodies against DDR signaling proteins such as ATM (pATM Ser1981) should be included.

We agree with this reviewer that γH2AX and 53BP1 foci are indeed markers of DNA double-strand breaks (DSBs), but it is equally correct to notice that they are widely used as markers of DDR activation, since their formation is fully dependent on upstream protein kinases controlling DDR signaling. Upon induction of DSBs, H2AX is rapidly phosphorylated at Ser139 by the PI3K-like kinases ATM, ATR, and DNA-PKcs to form γH2AX, which acts as a platform for the recruitment and retention of key DDR factors (Kinner et al, NAR, 2008). 53BP1 recruitment is a downstream step in the DSB signaling involving ATM and a key effector of the canonical DDR cascade, recruited to damaged chromatin. Its foci formation is a well-established readout of DDR activation (Panier and Boulton, Nat Rev Mol Cell Biol, 2013). Numerous studies (Carron et al, Cell Rep, 2015; Jun et al, Oncotarget, 2016; Sordet et al, EMBO Rep, 2009), use the reduction of γH2AX and 53BP1 foci as functional readouts of attenuated DDR signaling, supporting their relevance as established markers of DDR modulation. In addition, we have previously demonstrated in independent publications that tASOs treatment reduces several DDR signaling markers, including ATM phosphorylated at serine 1981 (pATM), phospho-Ser/Thr Q motif-containing proteins (pS/TQ), and KAP1 phosphorylated at serine 824

(pKAP1), further supporting a direct impact of tASOs on DDR signaling (Rossiello et al, Nat Comm, 2017, see Fig. 4; Aguado et al, Nat Comm, 2019, see Fig. 2g and 3c). Nonetheless, to further strengthen our conclusion that tASOs impair tDDR signaling also in the settings described in this manuscript, we studied additional DDR markers by immunohistochemical staining, namely pATM, the autophosphorylated and activated form of ATM, and pKAP1, a phosphorylation event specifically mediated by ATM (Goodarzi et al, Nat Struc Mol Biol, 2011). Both markers are increased in G3 Terc-/- lungs and are significantly reduced following tASOs treatment (Fig. 2d-e, also shown below), recapitulating what observed for γH2AX and 53BP1.

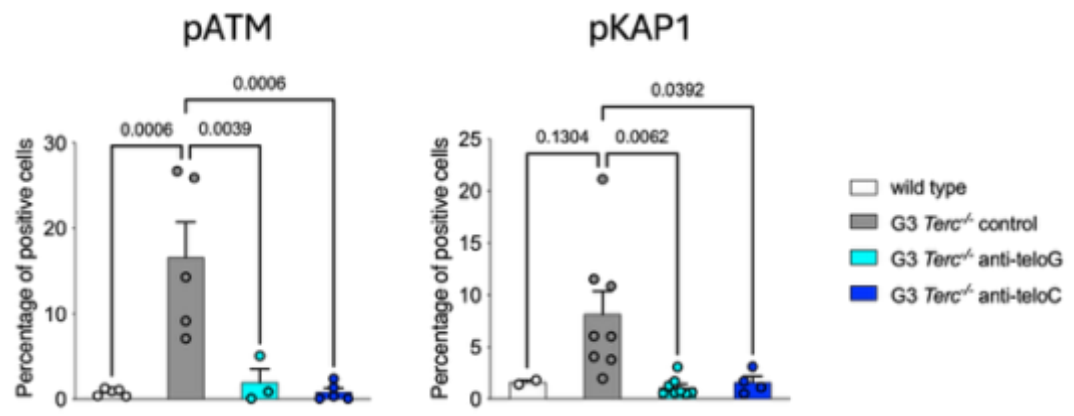

3. The authors should assess whether tASO treatment specifically reduces DNA damage at telomeres of G3 Terc⁻/⁻ mice. This could be achieved by analyzing telomere dysfunction-induced foci (TIFs) in treated and untreated G3 Terc⁻/⁻ lungs.

We agree that directly assessing the reduction of DNA damage at telomeres is important. Indeed, we demonstrated the ability of tASOs to specifically reduce telomere-associated DNA damage in multiple independent experimental systems. In Aguado et al, Nat Comm, 2019, we showed that tASOs treatment markedly reduced TIFs, defined by the colocalization of 53BP1 or pKAP1 foci with the telomeric marker TRF2, in both human and mouse cells, including progerin-expressing human fibroblasts, as well as *in vivo* in mouse epidermis (see Figures 1c, 2g, and 3d)
In addition, in a separate study currently submitted in the same Terc⁻/⁻ model with similar tASO treatments, we directly assessed TIFs in the hematopoietic system. We performed immuno-FISH analyses to quantify γH2AX colocalization with telomeric DNA in bone marrow cells from Terc⁻/⁻ mice treated or not with tASOs and observed that untreated Terc⁻/⁻ mice displayed significantly higher levels of TIFs compared with age-matched wild-type controls, and that tASOs treatment markedly reduced telomere-associated γH2AX signals (see Figure below).

Figure for reviewers removed

To allow TIFs detection also in this tissue, and avoid the high intrinsic autofluorescence that limits standard immunofluorescence approaches, we performed an *in situ* proximity ligation assay (PLA) between the telomeric protein TRF2 and the DDR marker γH2AX in lung sections from G3 Terc⁻/⁻ mice using a chromogenic readout, ensuring robust and

unambiguous visualization of PLA signals. This approach allows the detection of close spatial proximity between telomeric chromatin and activated DDR signaling directly in tissue samples. We observed a marked increase in TRF2-γH2AX PLA signals in the lungs of control G3 Terc⁻/⁻ mice compared with wild-type controls, consistent with enhanced telomere-associated DDR activation. Importantly, tASOs treatment significantly reduced the number of PLA-positive signals (Fig. 2f, also shown below), indicating a robust attenuation of telomeric DDR *in vivo*.

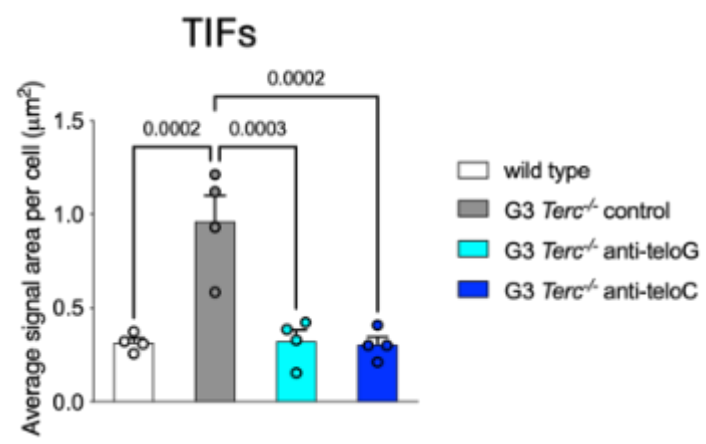

Altogether, these data provide direct *in vivo* evidence that tASOs reduce telomere-associated DDR signaling, supporting a telomere-specific mechanism of action.

4. Given the central role of critically short telomeres in telomere-driven lung dysfunction (PMID: 38424230), telomere length should be measured in the lung parenchyma of WT, G3 Terc⁻/⁻, and tASO-treated G3 Terc⁻/⁻ mice. Southern blot or, preferably, TeSLA that would provide informative data on the shortest telomeres.

We thank the Reviewer for highlighting the importance of measuring telomere length in the lung parenchyma. In addition to the FISH analysis already shown in the manuscript which showed no change in telomere length in the lungs of tASOs-treated mice, we have now included a telomere restriction fragment (TRF) analysis carried out by Southern blot (Fig. EV 3g-h, also shown below).

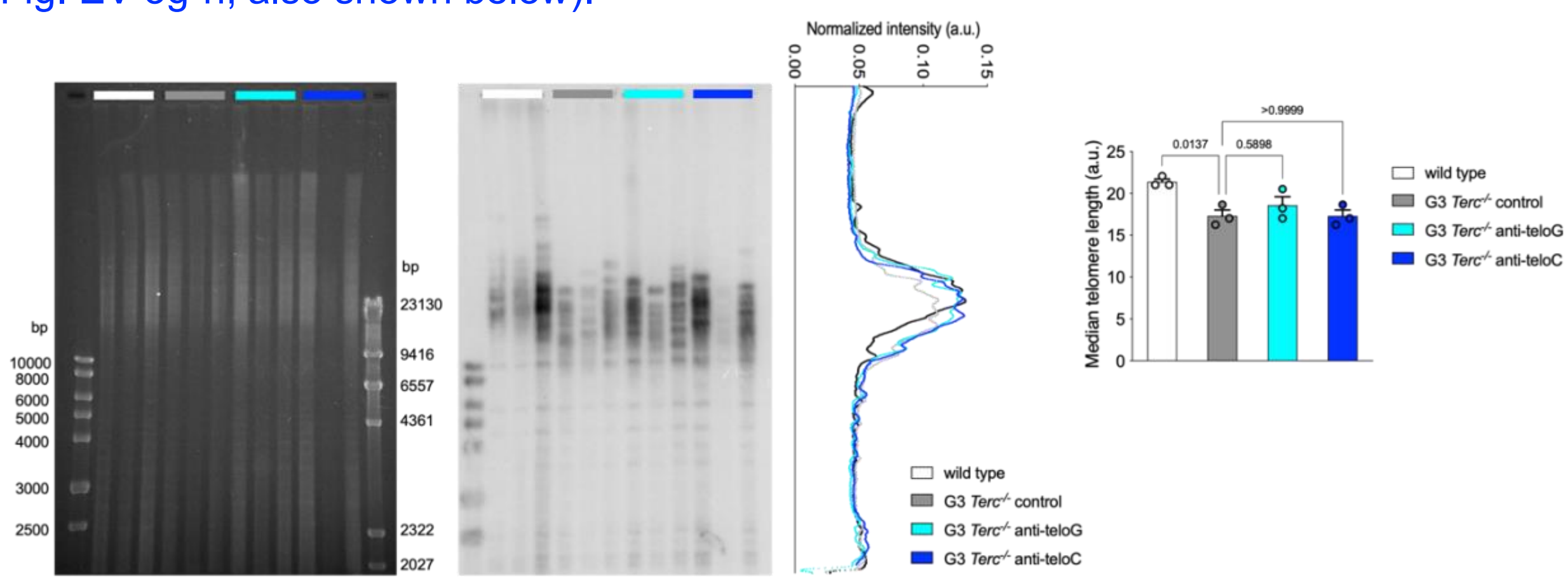

It confirmed and strengthened the FISH results, showing telomere shortening in G3 Terc-/- mice and no alterations induced by tASOs treatments. In addition, to provide a comprehensive and accurate measure of telomere lengths, we also show the full distribution of telomere lengths obtained from the Southern blot analyses. Notably, signals

at the lower end of the gel, corresponding to short telomeres, remain unchanged across conditions.
These consistent results confirm that critically short telomeres are not decreased in the tASO-treated G3 Terc⁻/⁻ mice.

5. Suppression of senescence and inflammation is currently inferred from transcriptomic signatures. These findings would be significantly strengthened by histological or immunohistochemical evidence, such as SA-β-Gal staining, or immunostaining for senescence markers like p21.

We thank the Reviewer for this suggestion. While transcriptomic signatures indicate suppression of senescence and inflammation, we further show that tASOs treatment reduces the fraction of γH2AX- and 53BP1-positive cells, indicating attenuation of DDR signaling. Persistent activation of DDR is a well-established hallmark and driver of cellular senescence (d'Adda di Fagagna, Nat Rev Cancer, 2008; Ogrodnik et al, Cell, 2024); thus, the observed reduction in DDR markers further supports the notion of the suppression of senescence-associated pathways.
As requested by this referee, to further reinforce the transcriptomic evidence, we have now performed immunostaining for the senescence marker p21 (Fig. 5d, also shown below). We observed a clear trend toward an increased number of p21-positive cells in G3 Terc-/- mice compared with age-matched wild type controls, which was significantly reduced by both tASOs.

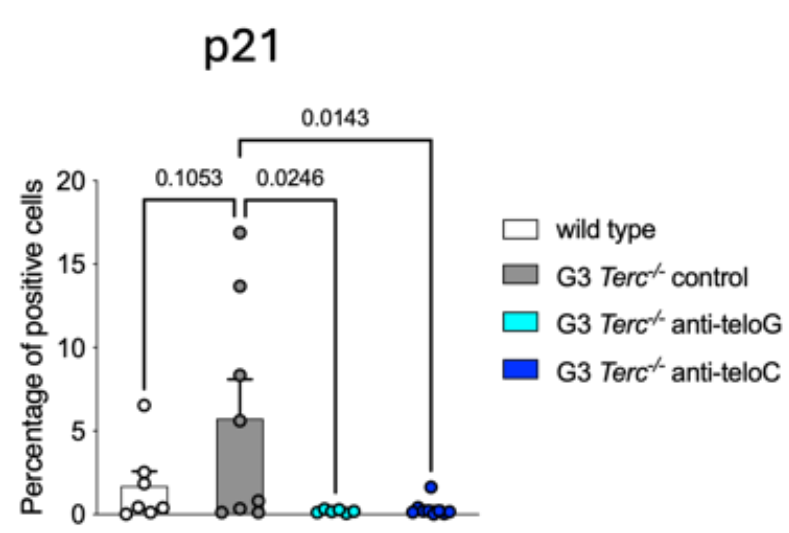

We believe that these findings are consistent with a suppression of senescence and inflammation by tASOs, a conclusion that is strongly supported by the transcriptomic data, which are more robust and sensitive to detect global changes in these pathways.

6. The authors should provide the full list of differentially expressed genes (DEGs) corresponding to Figures 3A, 3B, and 3C in an Excel file. Additionally, volcano plots highlighting the top 20 upregulated and downregulated genes would improve data. In Figure 4, the senescence and inflammation-related genes displayed in the heatmaps should be clearly indicated.

We thank the Reviewer for this suggestion. We have now provided the full list of differentially expressed genes (DEGs) in the comparison old wild type vs adult wild type in the Dataset EV1, and the DEGs in the comparison adult control G3 Terc-/- vs adult wild

type in the Dataset EV2. In addition, volcano plots highlighting the top 20 upregulated and downregulated genes have been included (Fig. EV 4a, also shown below).

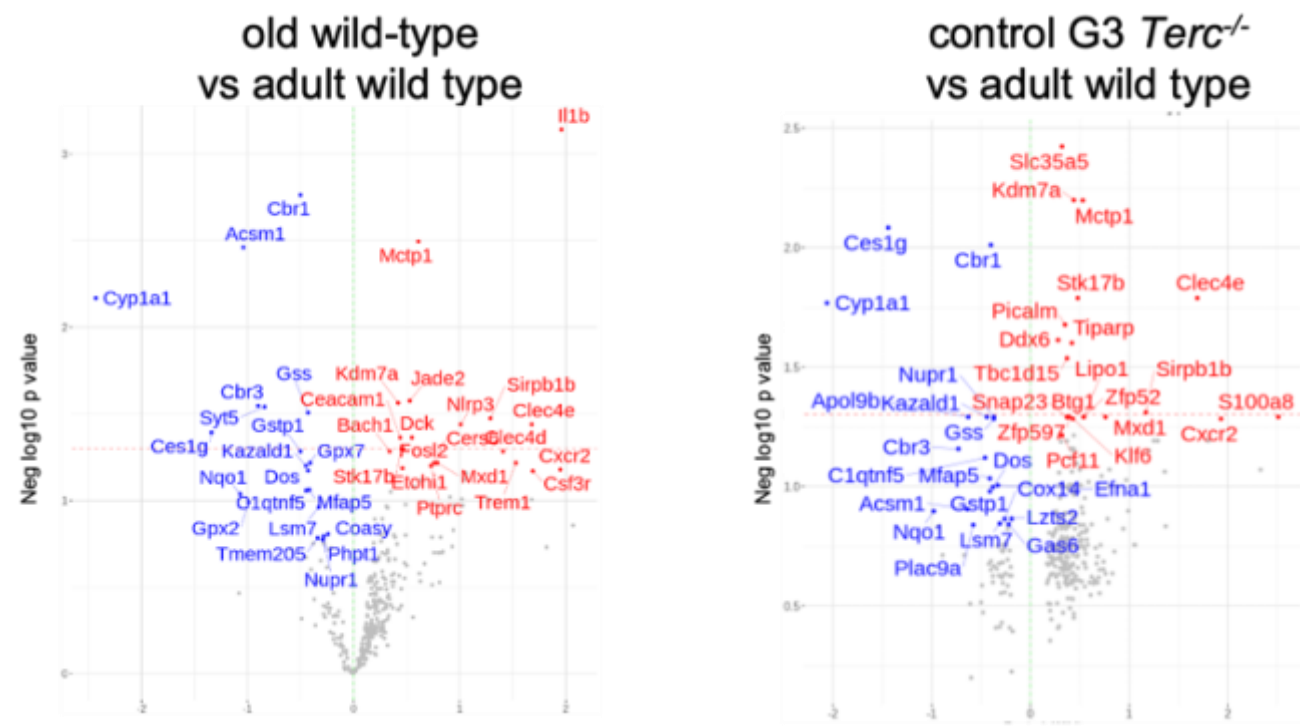

With regard to Fig. 4 (now Fig. 5), the senescence-related genes displayed in the heatmaps are now clearly indicated (Fig. EV 5d, also shown below), improving clarity and interpretability of the data.

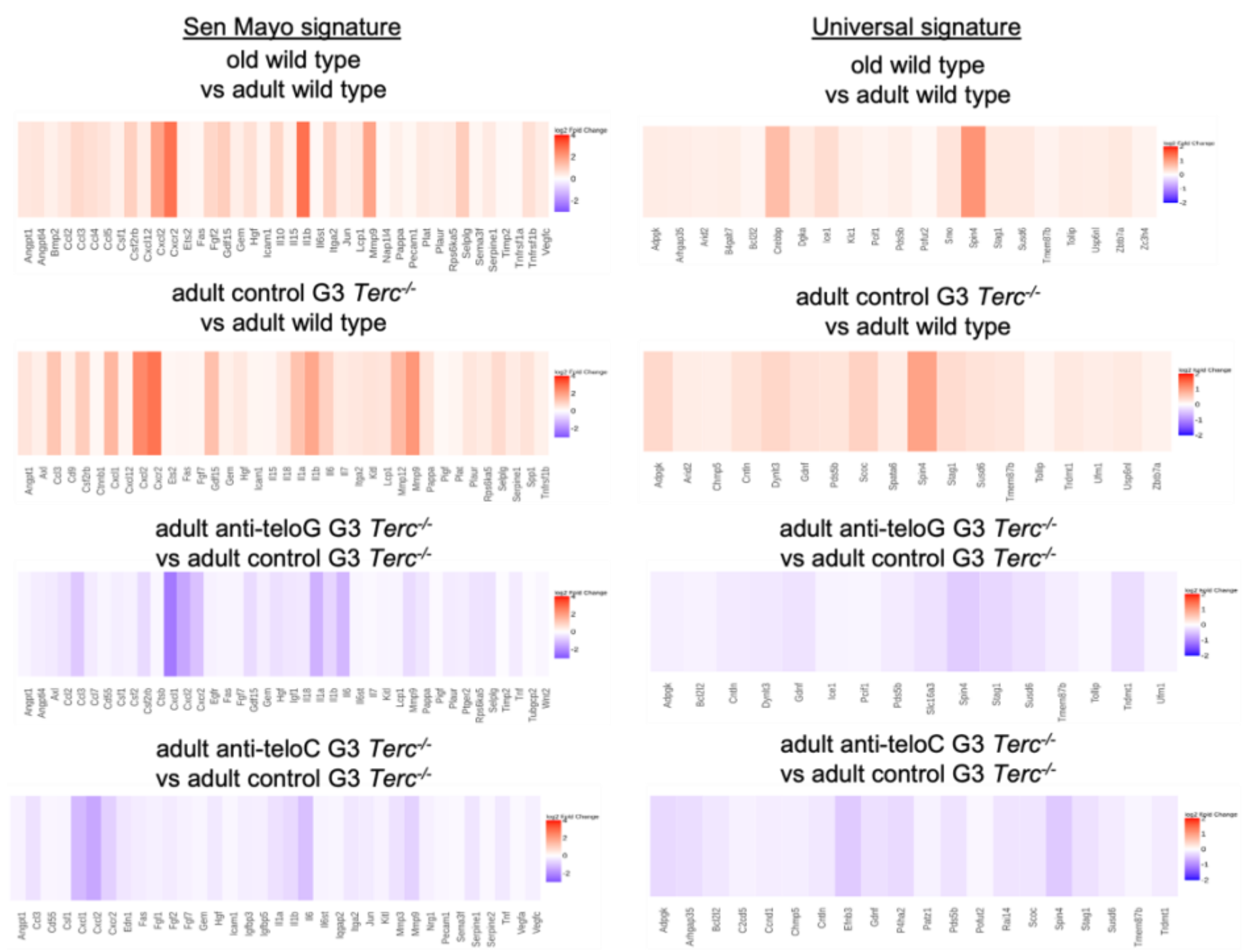

7. Bulk RNA-seq reflects average gene expression across multiple cell types. Could the author perform cell-type deconvolution using available lung single-cell RNA-seq references to identify which cell populations contribute most to the observed DEGs.

We thank the reviewer for this valuable suggestion. To address this point, we performed cell-type deconvolution by using in parallel three independent algorithms: CIBERSORTx, ImmuCC, and xCell. When running CIBERSORTx, we used the single-cell RNA-seq lung datasets from Tabula Muris as the reference matrix. We reported the average deconvolution scores per group (Fig. EV 6a-c, also shown below), providing a consistent estimate of the relative abundance trends of cell types across the experimental

conditions. The average deconvolution scores were relatively homogeneous across groups, except for neutrophil scores, which were lower in the adult wild type group.

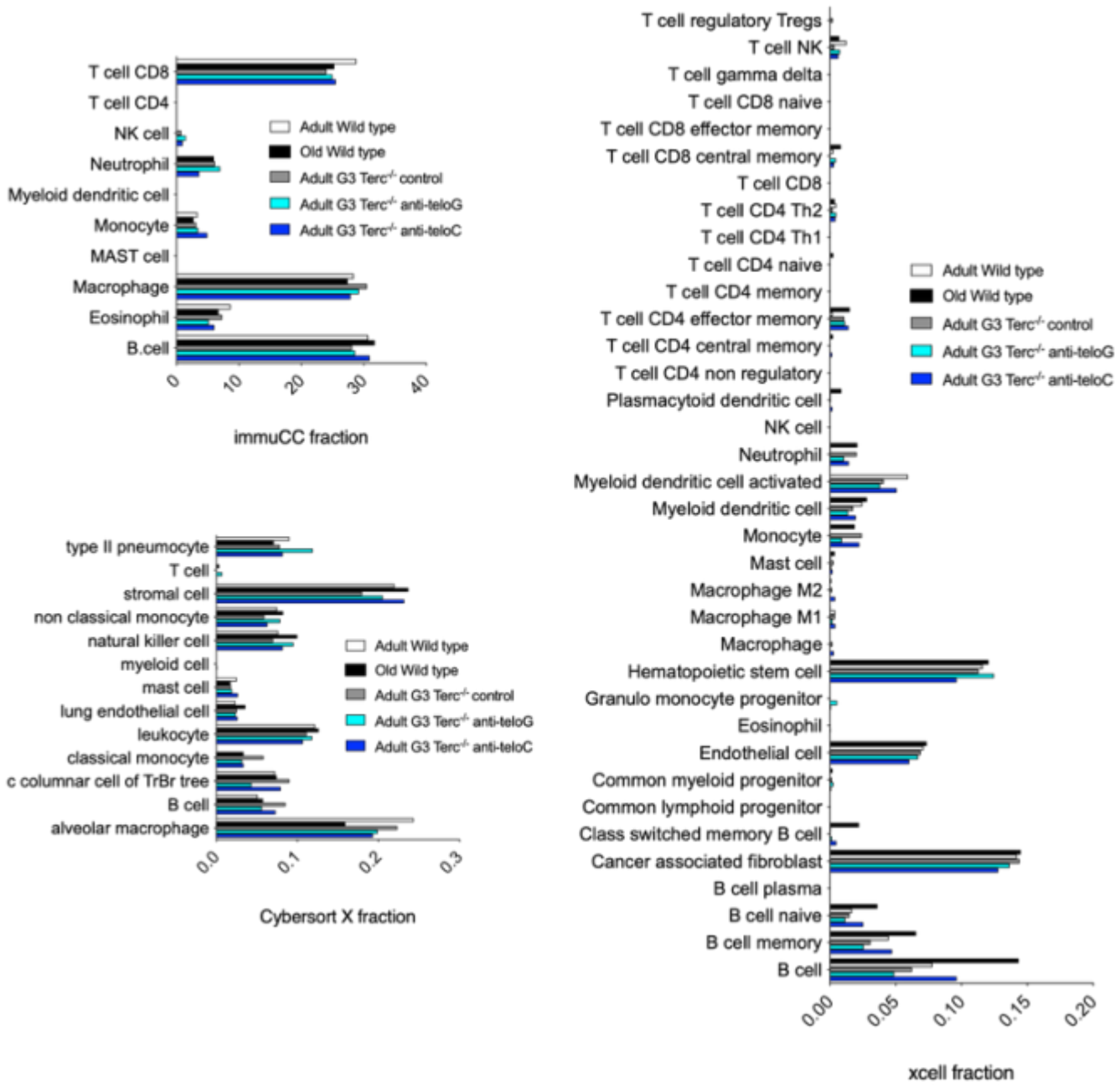

To investigate how specific cell populations relate to the patterns of differential gene expression, we performed a rank-based GSEA using the $\log_2$ fold-change–ranked gene lists obtained from the four pairwise comparisons: old wild type vs. adult wild type, adult control G3 Terc-/- vs. adult wild type, adult anti-teloG G3 Terc-/- vs. adult control G3 Terc-/-, and adult anti-teloC G3 Terc-/- vs. adult control G3 Terc-/-. Cell-type marker gene sets were retrieved from PanglaoDB, focusing on mouse canonical markers associated with the immune system and lung tissue.

In the first two comparisons, the genes upregulated in old wild type and control G3 Terc-/- samples (each relative to adult wild type) were significantly enriched for neutrophil-associated markers, indicating an increased representation or activation of neutrophil-related transcriptional programs (Fig. EV 6d-e, also shown below).

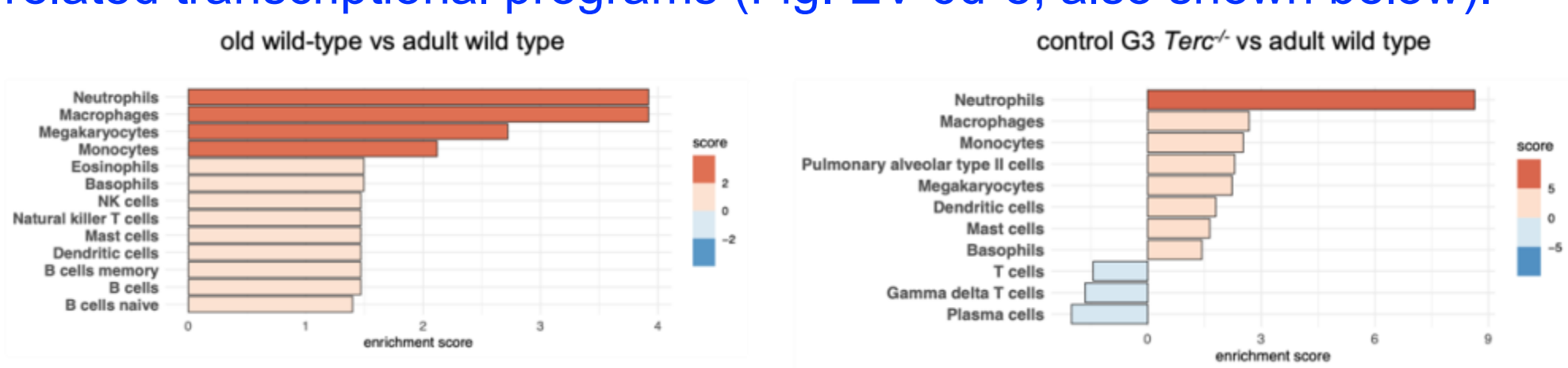

When we extended the analysis to the remaining comparisons, we observed the opposite trend: genes upregulated in adult anti-teloG G3 Terc-/- and adult anti-teloC G3 Terc-/- samples (relative to adul control Terc-/- samples) were negatively associated with the neutrophil signature (Fig. EV 6f, also shown below).

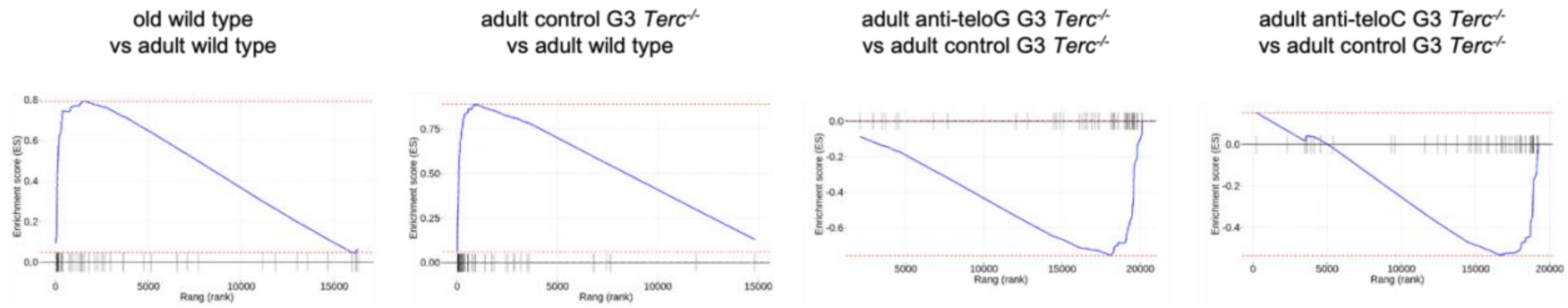

This reversal suggests a depletion or downregulation of neutrophil-related transcriptional activity in the tASOs-treated conditions, and thus a reduction of active inflammation, consistent with the decrease of CD45-positive cells (Fig. 5g).

8. The manuscript shows reduced γH2AX and 53BP1 foci in ATII cells following tASO treatment. It would be very informative to assess whether this reduction is specific to ATII cells or also occurs in other lung cell types, such as endothelial or fibroblast populations.

In order to address this referee's request, we performed double immunostaining for vimentin, a marker of mesenchymal cells, including fibroblasts and endothelial cells, and the DDR marker γH2AX in lung tissue (Fig. EV 7b, also shown below). This analysis revealed a significant increase in γH2AX-positive cells within vimentin-positive cells in G3 Terc-/- mice compared to age-matched wild-type controls, indicating elevated DDR in the mesenchymal compartment. Notably, treatment with tASOs significantly reduced γH2AX positivity, demonstrating an effective attenuation of DDR upon telomere-targeting intervention also in this cell type.

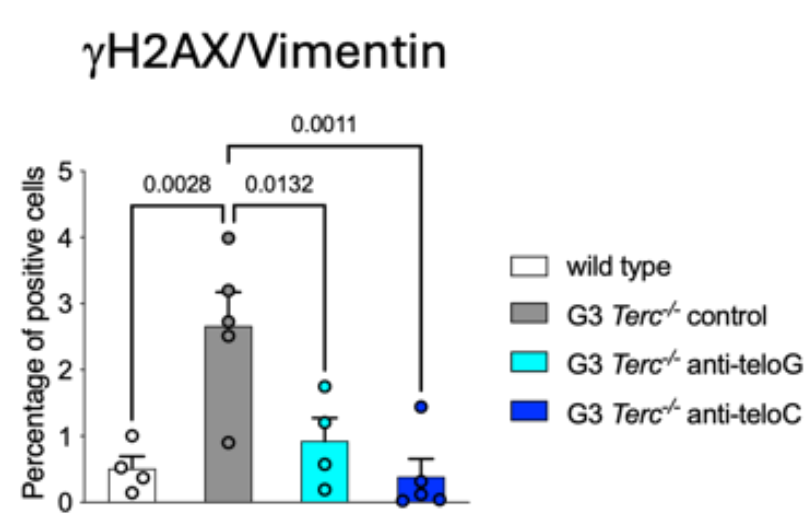

Therefore, tASOs treatment impact is confirmed in other lung cell types, in addition to that reported in ATII cells.

Referee #3 (Remarks for Author):

The manuscript by Rossiello et al., entitled "Telomeric DNA damage response as a target for the treatment of Idiopathic Pulmonary Fibrosis" presents an interesting and well-structured study. The authors demonstrate that activation of the telomeric DNA damage response (tDDR) can lead to the spontaneous development of lung fibrosis and associated pathologies in mice, even in the absence of external/additional injury (e.g., bleomycin-induced lung fibrosis). Using a mouse model lacking the RNA component of

telomerase (Terc), they show that Terc-knockout mice develop fibrosis as early as 3 months of age, a phenotype that persists into adulthood at 12 and 20 months. Furthermore, the authors validate the antifibrotic efficacy of two antisense oligonucleotides (ASOs) in both adult and aged mice. The RNA-seq data are thoroughly analyzed, and the comparison of mice data with human idiopathic pulmonary fibrosis (IPF) datasets reveals notable similarities.

Overall, the manuscript is well-written and the figures are compelling. However, important key RNA-seq datasets and immunofluorescence (IF) quantifications that would strengthen the authors' conclusions are currently missing.

We thank Referee 3 for the encouraging evaluation of our work, and for highlighting both the strengths of the study and areas where additional data could further support our conclusions. In response to the Reviewer's comments, we have conducted additional immunohistochemical stainings, a dedicated RNA-seq experiment, and further transcriptomic analyses to strengthen the mechanistic and pathological evidence presented. These revisions enhance the rigor and transparency of the study. Detailed point-by-point responses are provided below.

Major Comments:

Figure 1:

The authors have validated the effect of Terc-knockout (Terc-KO) in inducing fibrosis in both young and aged mice. However, it is essential to identify the specific lung cell populations affected by Terc-KO.

• Determine whether the frequency of alveolar epithelial type 2 (AT2) cells changes in Terc-KO mice.

We thank the Reviewer for this comment. While telomere damage in alveolar type II (ATII) epithelial cells is well-established as a key driver of interstitial lung fibrosis (Alder et al, PNAS, 2015; Povedano et al, Cell Rep, 2015; Liu et al, JBC, 2019), it was important to assess whether Terc-/- alters the frequency of these cells in our model. In our manuscript, we show that the frequency of ATII (pro-SPC-positive) cells is not significantly different in adult G3 $Terc^{-/-}$ mice compared with age-matched wild-type controls (Fig. EV 7a). We have now extended this analysis to young mice and similarly observed no significant differences between genotypes (see Fig. below).

Figure for reviewers removed

These findings suggest that in this model fibrosis is not driven by changes in ATII cells abundance but rather by the presence of damaged (DDR-positive) ATII cells. Notably, tASO treatment reduces DDR markers in ATII cells (Fig. 6a-b), supporting the conclusion that these cells are a key therapeutic target of our telomere-directed intervention.

• Assess whether Terc-KO mice exhibit elevated TGF-β signaling pathway activity (e.g., IF stains for phospho-Smad2/3).

We thank the Reviewer for this suggestion. To assess whether G3 Terc-/- mice exhibit increased TGF-β signaling pathway activity, we performed immunohistochemical staining for phosphorylated SMAD2/3 (pSMAD2/3), a canonical downstream effector of TGF-β signaling (Massagué, Nat Rev Mol Cell Biol, 2012). We observed a marked increase in pSMAD2/3 staining in lung sections from Terc-/- mice compared to wild type controls, indicating enhanced TGF-β pathway activation. Importantly, treatment with tASOs significantly reduced pSMAD2/3 levels, consistent with an attenuation of TGF-β signaling (Fig. 3e, also shown below).

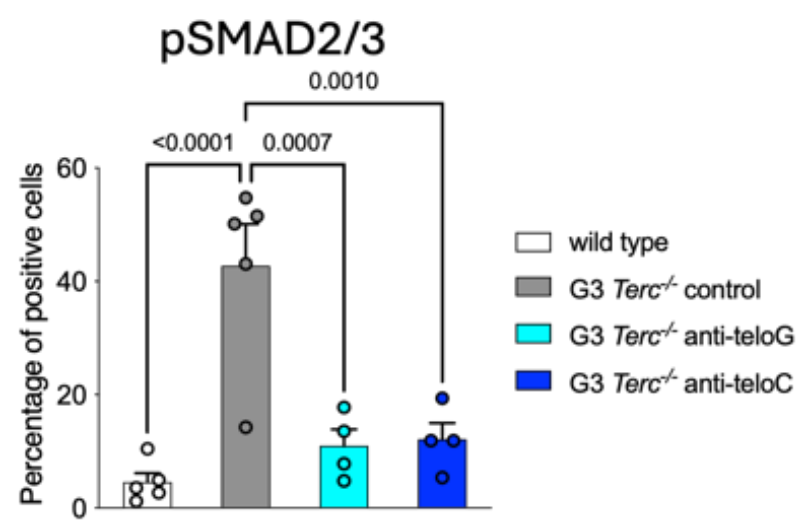

Importantly, these findings were corroborated at the transcriptomic level. GSEA revealed significant enrichment of the TGF-β signaling pathway in Terc⁻/⁻ lungs, which was reduced upon tASOs treatment (Fig. EV 5a, also shown below).

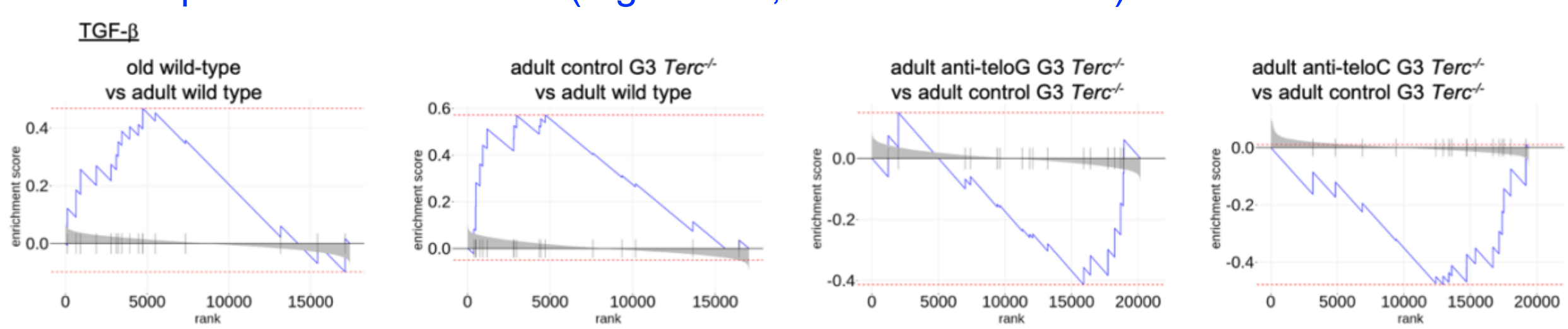

Together, the increased pSMAD2/3 immunostaining and pathway-level transcriptomic enrichment support the conclusion that telomere dysfunction in Terc-/- mice is associated with increased TGF-β activity, which is mitigated upon tASOs treatment.

Figure 2 and EV2 Results Section:
The manuscript refers to signs of "emphysema". This term may be inappropriate in this context. Decreased fibrosis and restoration of alveolar architecture do not necessarily indicate emphysema, particularly without a significant increase in airspace size/number between wild-type controls and ASO-treated mice. Reconsider the terminology used.

We thank the Reviewer for this important clarification and agree that the term “emphysema” may be inappropriate in the absence of dedicated morphometric quantification of airspace enlargement. While our study did not include standardized lung inflation/fixation or whole-lung stereology — methods required for a formal diagnosis of

emphysema — we did quantify airspace number, as reported in the manuscript, and this measurement supports the presence of enlarged and dysregulated alveolar spaces in Terc⁻/⁻ mice. To avoid overinterpretation, we have revised the terminology throughout the manuscript and now refer more cautiously to "emphysema-like" features when describing the morphological changes observed in Terc⁻/⁻ mice. This wording better reflects the descriptive nature of our analysis and avoids implying a formal diagnosis of emphysema without appropriate quantitative measurements.

Figure 2 and EV2:
The data demonstrate attenuation of fibrosis and restoration of airspaces in ASO-treated mice, but information on the cellular outcomes in fibrotic lungs is missing.

• Determine whether activated myofibroblasts (aMYFs) are undergoing apoptosis, as this is one of the key mechanisms in fibrosis resolution.

We agree that aMYF apoptosis can contribute to fibrosis resolution in acute injury models. Prompted by this Referee's suggestion, we performed a double immunohistochemistry for αSMA and Cleaved Caspase-3, which however did not reveal significantly detectable numbers of double-positive cells, indicating an absence of apreciable apoptotis in aMYF at the time points examined. This finding does not exclude a contribution of apoptosis to myofibroblast clearance, rather it suggests that in this model it could be a potential apoptotic wave which remains undetectable given the long intervals between treatment and tissue collection (2 and 9 months).
It is also worth mentioning that apoptosis is only one of several recognized — and not mutually exclusive — mechanisms that can mediate fibrosis resolution. Additional events may involve myofibroblast dedifferentiation, non-apoptotic forms of myofibroblast loss, immune-mediated clearance, enhanced ECM turnover, and upstream suppression of profibrotic signaling that limits sustained myofibroblast activation (Horowitz and Thannickal, Physiology, 2019).

• Include IF staining and quantification of aMYF markers (α-SMA, Col1a1).

As suggested by this Referee, we quantified the levels of α-SMA and Col1a1 by immunohistochemistry in adult mice. Increased α-SMA was observed in G3 Terc⁻/⁻ mice compared with wild-type controls, which was significantly reduced following tASOs treatment (Fig. 3c, also shown below).

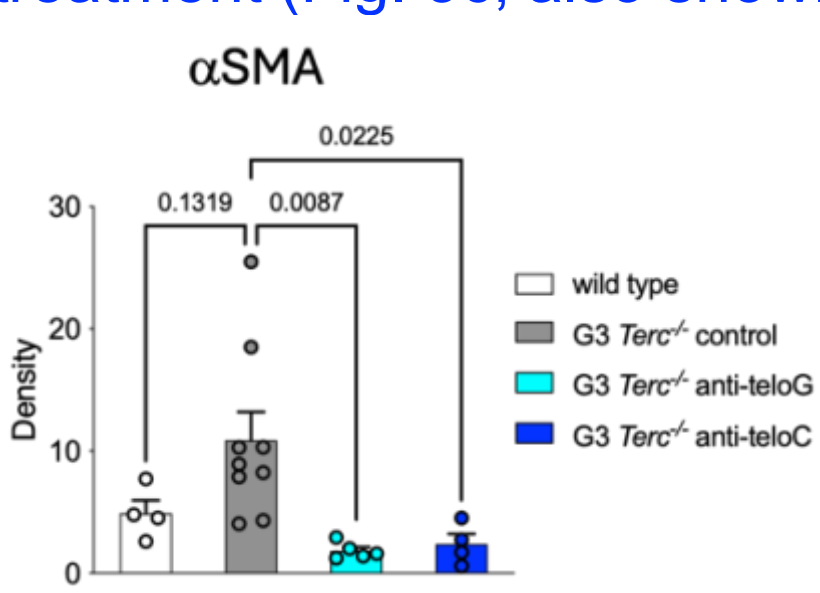

Similarly, Col1a1 levels were markedly reduced upon tASOs administration (Fig. 3d, also shown below), paralleling the changes observed for α-SMA.

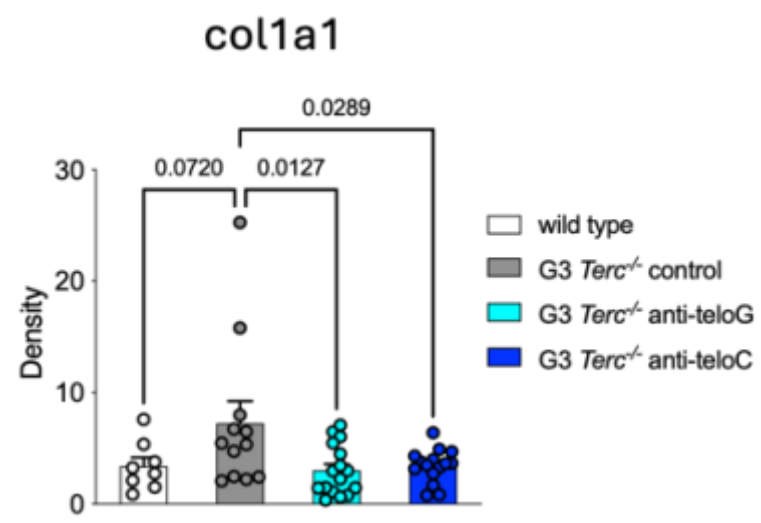

These results provide additional evidence that tASOs attenuate myofibroblast activation and extracellular matrix deposition in the fibrotic lung.

Figures 3 and 4:
Since 3-month-old Terc-KO mice lungs show fibrosis, include a comparison between 3-month-old Terc-KO and age-matched wild-type mice (in panels A or a separate EV figure) to assess aging and senescence signatures as early as 3 months of age.

To directly address this request, we performed a dedicated RNA-seq experiment on lungs from 3-month-old G3 Terc^{-}/$^{-}$ and age-matched wild-type mice. Gene set enrichment analyses (GSEA) revealed a significant enrichment of both the Universal Senescence Signature and the Sen Mayo senescence gene sets in young Terc-/- lungs compared with age-matched controls (Fig. EV 5f, also shown below).

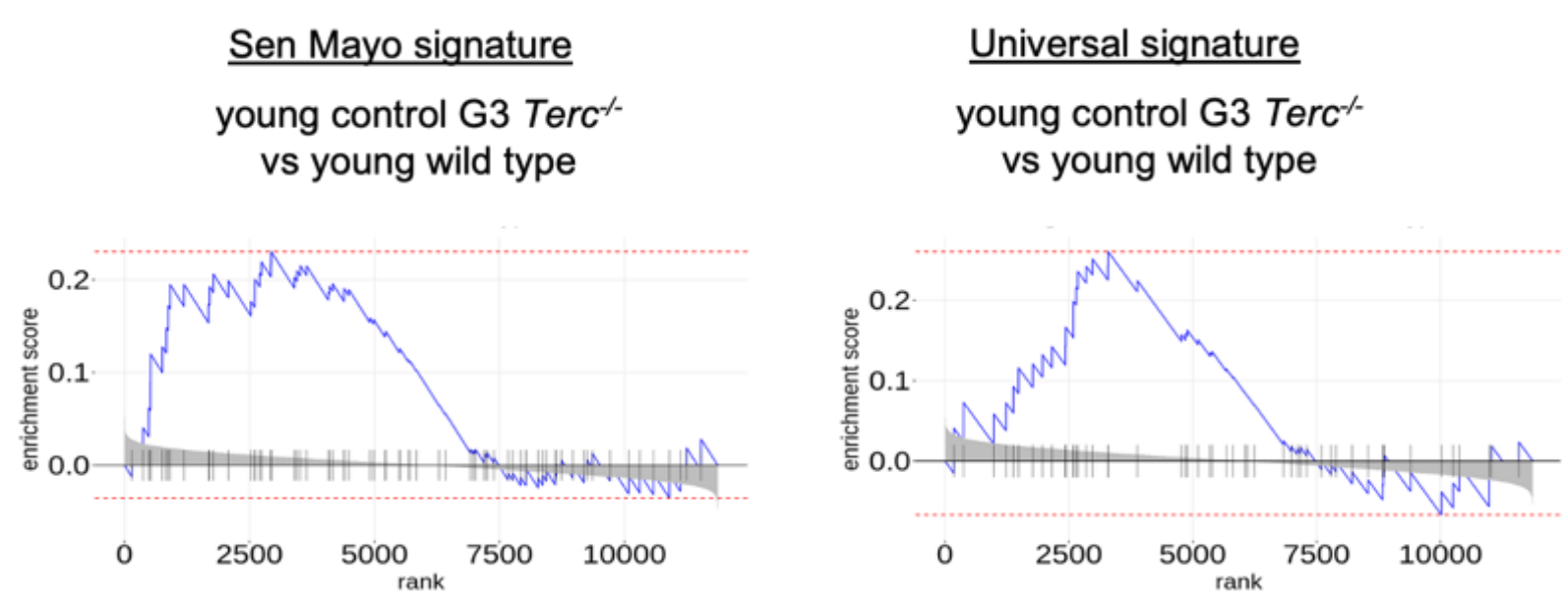

These findings indicate the early activation of senescence-associated transcriptional programs in young G3 Terc-/- mice.

Figure 4:
In addition to the current GSEA plots (e.g., SenMayo, Universal senescence signatures, inflammatory response), include GSEA plots for fibrotic signature (aMYF markers), AT2/AT1 cell signatures, and Krt8^{+} (AT2-AT1) transitional cell's signature.

We thank the reviewer for this valuable suggestion. In addition to the GSEA plots already included in the previous version of the manuscript (Sen Mayo, Universal senescence signatures, inflammatory response), we have now expanded our analysis to include gene set enrichment analyses for fibrotic signatures.

Specifically, we performed GSEA using two fibrotic-related signatures, namely the "lung fibrosis" and "myofibroblast differentiation" gene sets, previously described (Strunz et al, Nat Comm, 2020; Lingampally et al, Aging Cell, 2025; Panagiotidis et al, Front Immunol, 2025). Both fibrotic signatures are significantly upregulated in old and $Terc^{-/-}$ animals compared with adult wild-type controls. Importantly, treatment with tASOs markedly reduced the enrichment of these fibrotic programs, indicating an attenuation of fibrosis-associated transcriptional responses (Fig. EV 5b-c, also shown below).

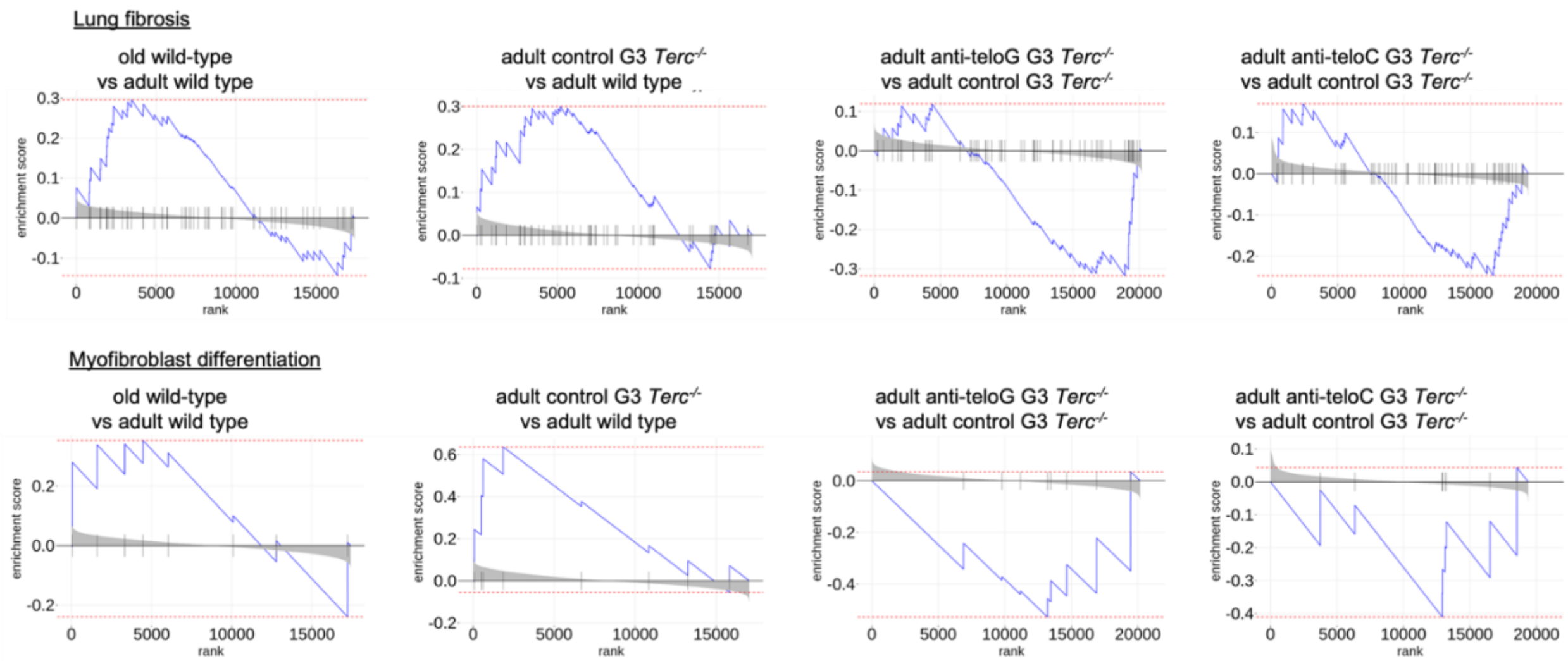

In addition, we examined gene set enrichment for alveolar epithelial cells-related signatures, including ATI, ATII, and Krt8-positive transitional cell programs (Strunz et al, Nat Comm, 2020). The comparison between $Terc^{-/-}$ and wild-type mice did not reveal statistically significant differences in their enrichment scores (ATI p-value adjusted = 0.93; ATII p-value adjusted= 0.93; Krt8+ p-value adjusted= 0.92) (see Figure below).

Figure for reviewers removed

It is also important to note that several of these epithelial cell state signatures were originally derived from bleomycin-induced lung fibrosis models. As such, they may not fully capture or accurately reflect epithelial alterations occurring in a genetic model of telomere dysfunction, where disease onset, progression, and cellular remodeling differ substantially from injury-driven fibrosis models.
Given the lack of robust and consistent transcriptional changes in these GSEA analyses, together with the potential limitations in applying bleomycin-derived signatures to our genetic model, we opted not to include these plots in the revised manuscript. Importantly, alterations in alveolar epithelial populations were clearly and consistently supported by our immunostaining analyses (see our reply below), which we therefore consider a more reliable and informative readout in this context. Consequently, we rely on the

immunostaining data to address epithelial cell alterations associated with telomere dysfunction and tASOs treatment.

Figures 5 and EV4:
AT2 cell frequencies appear unchanged across the experimental groups. However, to better assess potential enhancement of AT2-to-AT1 differentiation, it is important to include analyses of proliferation status, as well as quantification of AT2 cells, Krt8^{+}transitional cells (AT2-AT1 intermediates) and AT1 cells across conditions.

We thank the reviewer for this constructive comment regarding ATII-to-ATI differentiation. In response, we quantified alveolar epithelial populations using established markers. Our data indicate that AT2 cell frequencies remain unchanged across all groups, including WT, G3 Terc^{-}/$^{-}$, and G3 Terc^{-}/$^{-}$ treated with both tASOs (Fig. EV 7a). This suggests that the observed phenotypes are not driven by alterations in ATII cell numbers.
In addition, as suggested by the Reviewer, we assessed the abundance of KRT8-positive transitional cells (ATII-ATI intermediate states) by immunohistochemistry. We did not detect appreciable differences in the percentage of KRT8-positive alveolar epithelial cells between wild type, G3 Terc^{-}/$^{-}$, and tASOs-treated G3 Terc^{-}/$^{-}$ lungs (Fig. EV 7d, also shown below).

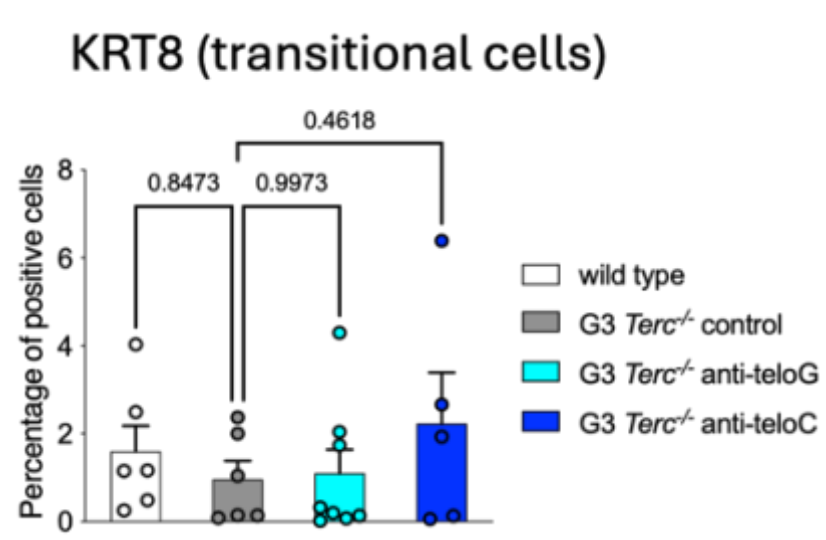

To further investigate potential alterations in the alveolar epithelial compartment, we focused on HOPX as a marker associated with the ATI lineage. HOPX has been widely used to assess activation of ATI-associated transcriptional programs in the adult lung, particularly in the context of epithelial injury and repair. Notably, previous work has demonstrated that HOPX expression is induced during ATII-to-ATI trans-differentiation *in vitro* and is upregulated *in vivo* in response to alveolar epithelial injury, including in murine models of lung fibrosis (Ota et al., Scientific Reports, 2018). In this context, increased HOPX expression reflects activation of an ATI differentiation program under stress or reparative conditions, rather than an expansion of fully differentiated AT1 cells. Consistent with this framework, we observed an increase in HOPX-positive epithelial cells by immunohistochemical staining in G3 Terc^{-}/$^{-}$ lungs (Fig. EV 7c, also shown below), which we interpret as indicative of stress-induced or maladaptive ATI lineage commitment in the setting of telomere dysfunction. Importantly, treatment with both tASOs resulted in a reduction in HOPX-positive cells, suggesting attenuation of this aberrant differentiation response and partial restoration of epithelial homeostasis.

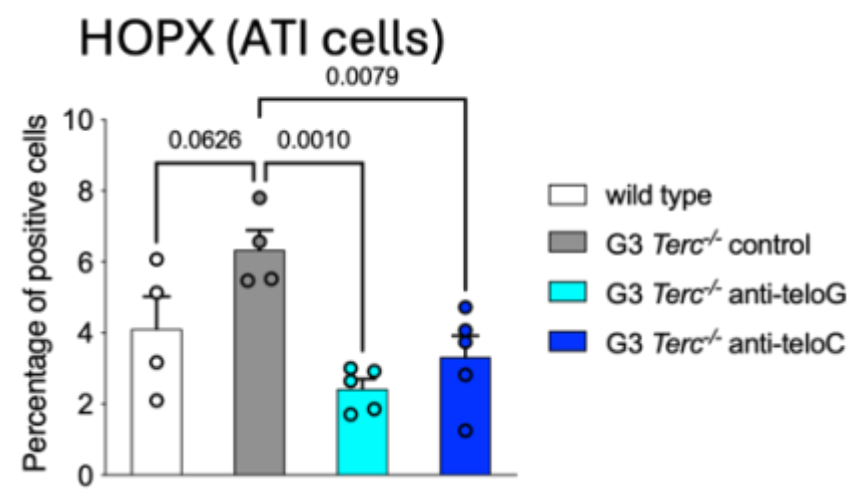

Finally, to address the Reviewer's suggestion regarding proliferation, we assessed alveolar epithelial cell proliferation by immunostaining for Ki-67. Across all experimental conditions, we did not detect any change in the percentage of Ki-67-positive cells (Fig. EV 7e, also shown below).

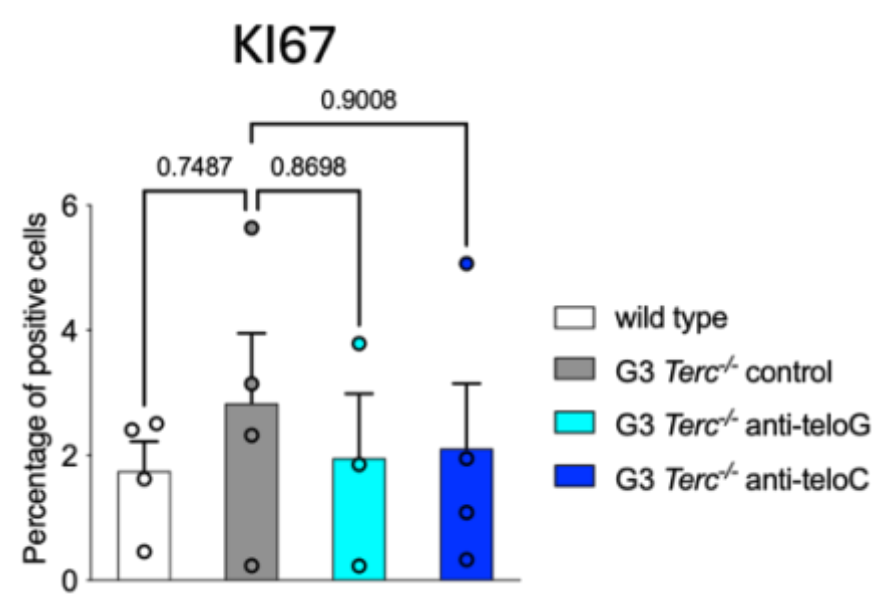

Taken together, these results suggest that telomere dysfunction does not induce major shifts in ATII abundance, ATII-ATI transitional populations, or epithelial proliferation. Instead, the epithelial alterations we observe are largely reflected at the ATI level, where increased HOPX expression indicates a stress-induced, aberrant activation of the differentiation program, which is mitigated by tASOs treatment. This supports the view that the phenotype arises from altered differentiation dynamics rather than from changes in epithelial lineage composition.

Figure 6:
The PCA plot shows that tDDR-treated samples cluster closer to wild-type mice but do not fully correlate or align. Include RNA-seq data from 3-month-old Terc-KO lungs to determine if treated aged mice exhibit similar transcriptomic profiles.

We thank the Reviewer for this suggestion. The RNA-seq datasets were not part of the original PCA analysis of the 12-month-old (adult) mice, and therefore they cannot be incorporated into the existing PCA. Nevertheless, to address this point raised by the reviewer, we instead performed a new PCA including all five available groups (adult wild type, adult Terc-/-, adult Terc-/- treated with tASOs, young wild type, and young Terc-/-), using all parameters that could be measured in all samples (Fig. EV 8a, also shown below).

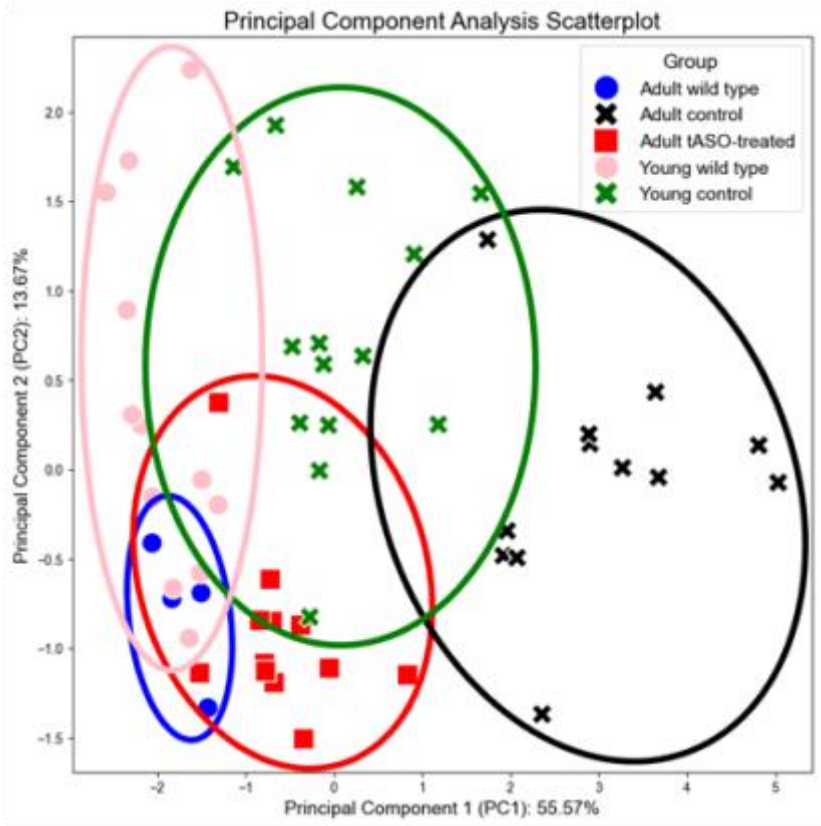

This analysis shows that tASOs-treated adult Terc-/- mice indeed shift their multi-parametric profiles toward the younger groups, indicating a partial rejuvenation effect. Importantly, a confusion matrix analysis confirms the PCA results (Fig. EV 8b, also shown below), demonstrating that the overall phenotypic profiles of tASOs-treated Terc⁻/⁻ lungs are more similar to the age-matched wild type group and the young groups than to untreated adult Terc⁻/⁻ mice.

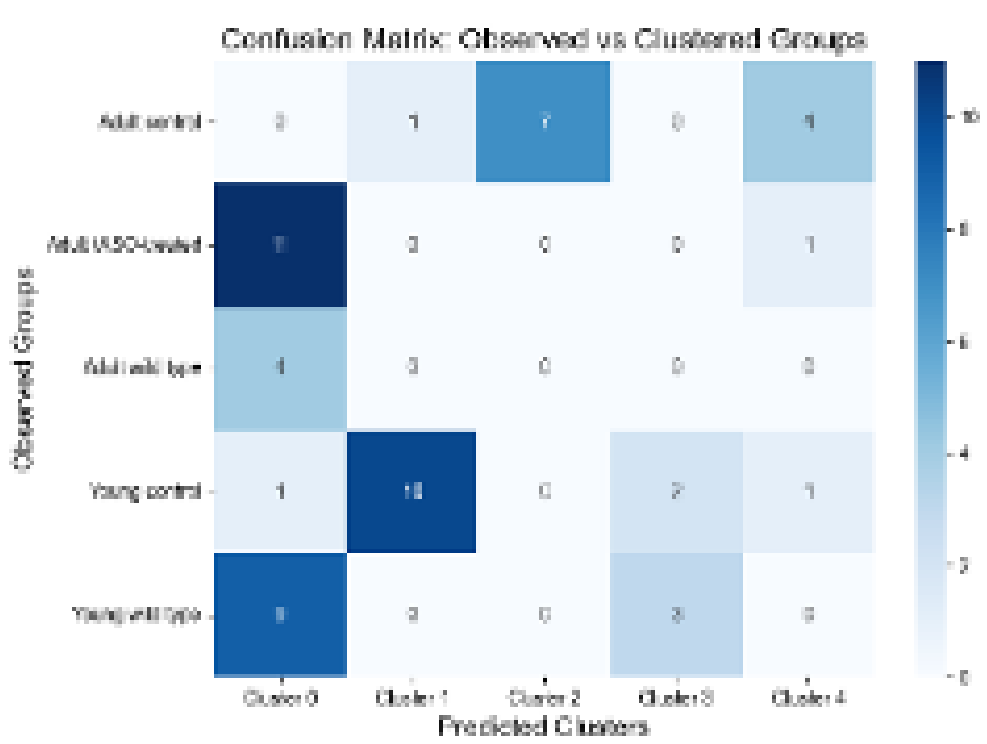

Thus, PCA clearly supports the notion that tASOs treatments mitigates age-associated transcriptional changes in Terc-/- lungs.

Figure 8:
Include a comparison between young Terc-KO and wild-type mice in panel A. Add GSEA plots showing TGF-β signature changes for additional mechanistic insight.

Prompted by the Reviewer's suggestion, we have now performed the comparison between young Terc-/- and wild-type mice and we have included it in the manuscript (Fig. 9a, also shown below).

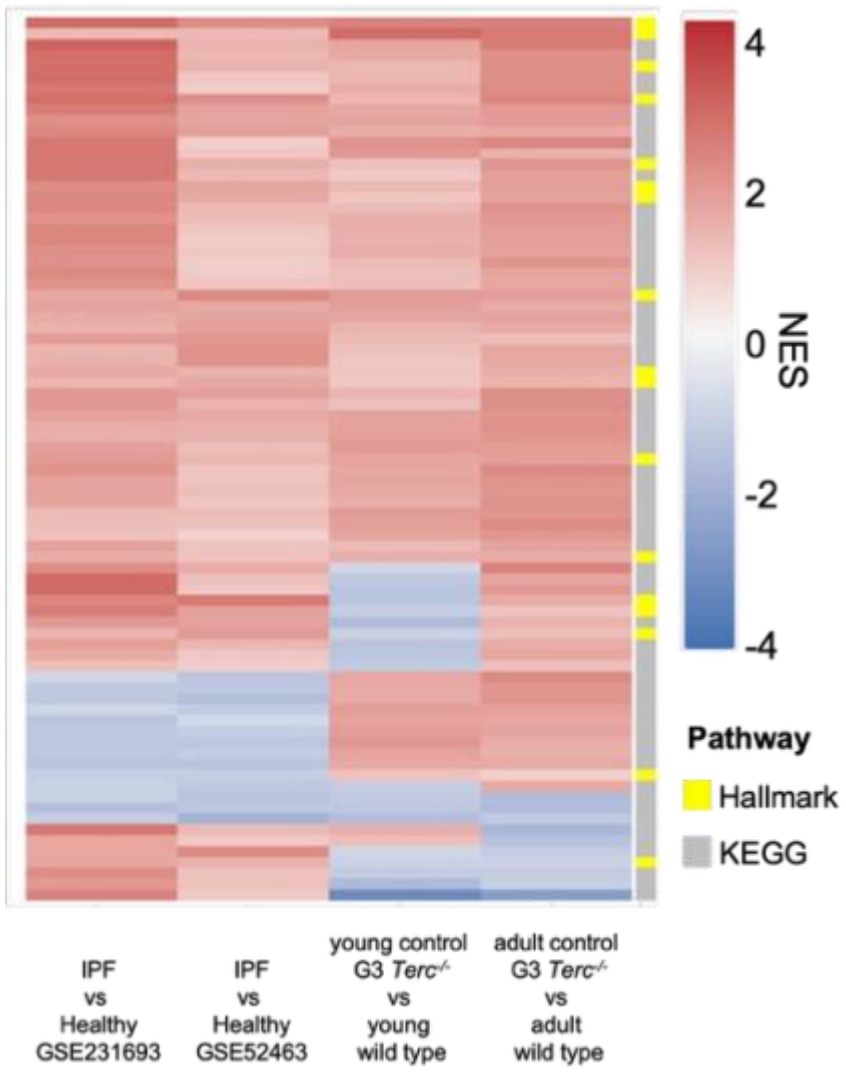

This analysis shows that the lungs of young telomerase-deficient mice already recapitulate most of the differentially-expressed pathways observed in the lungs of human IPF patients.
In addition, we have now included GSEA plots of the TGF-β signaling signature, which show that Terc-/- mice display increased activation of this pathway, and that this enrichment is attenuated following tASOs treatment (Fig. EV 5a, also shown below).

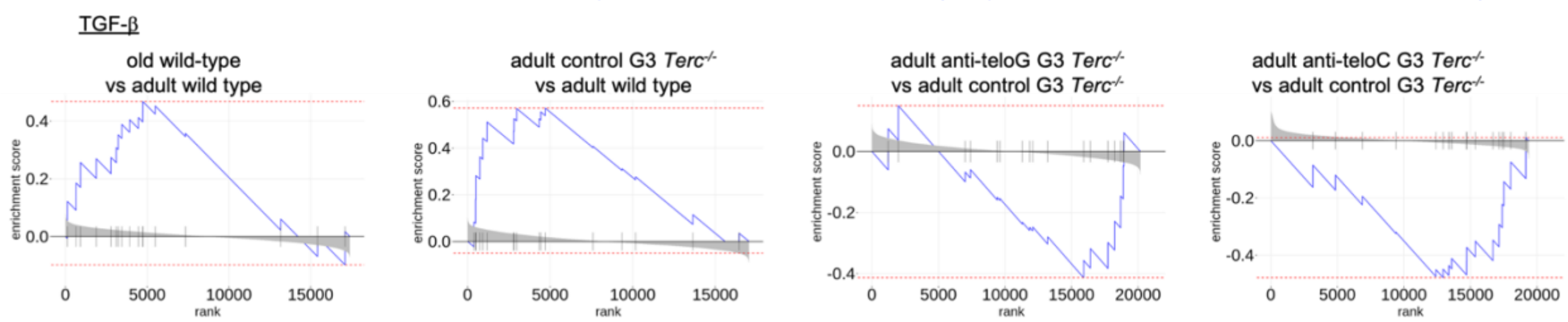

Minor Comments:

• Indicate the number of mice used in each experiment.

We have indicated the number of mice used for each experiment in the figure legends.

• Separate and align the in vivo ASO treatment schematics with the relevant data. Figure 2 uses 12-month-old mice, while Figure EV2 uses 5-month-old mice, schematics should reflect this distinction.

We have updated the schematics to separately reflect the *in vivo* ASO treatments in 12-month-old (Fig. 2a) and 5-month-old mice (Fig. EV 2a), aligning them with the corresponding data.

• Reconsider the use of the term emphysema in the manuscript, as previously noted.

We have reconsidered the terminology and revised the manuscript accordingly.

• Expanding the discussion on the Terc-knockout mouse model would provide valuable insights. Despite developing fibrosis by 3 months, the mice survive up to 20 months, unlike human IPF patients who typically survive 4-5 years post-diagnosis. Discuss possible explanations for this discrepancy.

Although G3 Terc-/- mice exhibit early signs of alveolar degeneration and fibrotic changes around 3 months of age, the development of an established and histologically evident fibrosis occurs only later in life, in line with the progressive nature of telomere-driven pathology. Importantly, as previously reported (Giorgio et al, Aging Cell, 2016), G3 Terc-/- animals indeed do show a reduced lifespan compared with wild-type controls and typically survive only slightly beyond the last time point analyzed in our study (~20 months), thus mirroring the human condition. We have added a brief paragraph addressing this point in the Discussion section.

27th May 2026

Decision on your manuscript EMM-2025-21982-V2

Dear Dr. d'Adda di Fagagna,

Thank you for submitting your revised manuscript to EMBO Molecular Medicine. We have now received feedback from all three original referees.

As detailed below, while Referees #2 and #3 are satisfied with the revisions, Referee #1 continues to raise substantial concerns regarding the robustness of the fibrosis phenotype, the rigor of its characterization, and the interpretation of the reported therapeutic effects.

Given the nature of these concerns, and in keeping with EMBO Press policy to allow only a single round of revisions within a limited timeframe, we regret that we are unable to consider your manuscript further.

While we cannot pursue this manuscript further, we encourage you to transfer your study to our not-for-profit open-access sister journal, Life Science Alliance (LSA). We shared your manuscript and the accompanying reviews with LSA Executive Editor, Tim Fessenden, who is interested in these findings. He is pleased to offer publication of this manuscript at LSA pending the following changes:
-moderation of claim of a promising therapeutic strategy (Reviewer 1, major concern, third paragraph).
-acknowledgement in the ms text of (1) the mild nature of the fibrosis phenotype and (2) lack of whole-lung biochemical validation (Reviewer 1, major concern first paragraph).

We encourage you to contact Dr. Fessenden at t.fessenden@life-science-alliance.org to discuss this work and the revisions requested. You may use the link below to immediately transfer your manuscript to LSA. You do not need to revise the manuscript before transferring it to LSA. Once you transfer, you will receive an invitation to revise and resubmit. Again, please contact Dr. Fessenden if you have any questions about the LSA journal, the transfer process, or the revisions requested

I am very sorry to disappoint you in this occasion, and hope that you will view the option to transfer favorably.

With kind regards,

Lise Roth

Lise Roth
Senior Editor
EMBO Molecular Medicine

***** Reviewer's comments *****

Referee #1 (Remarks for Author):

After reviewing the revised manuscript and the authors' responses, I appreciate the substantial additional work performed by the authors to address the reviewers' comments. The added analyses, including Ashcroft scoring, additional immunostaining, transcriptomic analyses, and mechanistic studies, clearly strengthened the manuscript compared with the original submission.

However, despite these efforts, I continue to have substantial concerns regarding the strength of the fibrosis phenotype, the rigor of the fibrosis characterization, and the interpretation of the therapeutic effects reported.

While the study addresses an important topic and the mechanistic concept of targeting telomeric DDR is interesting, I remain unconvinced that the data sufficiently support the central conclusion that tASOs meaningfully reverse pulmonary fibrosis in this model.

My major concerns are as follows:

The fibrotic phenotype in the Terc-/- model appears relatively modest and relies heavily on histological interpretation and image-based quantification. Although the authors added Ashcroft scoring and additional immunostaining analyses, the overall fibrosis burden remains difficult to appreciate convincingly at the whole-lung level.
Importantly, the authors report no significant increase in several canonical fibrosis-associated transcripts (Col1a1, Col1a2, Acta2/α-SMA, and Fn1) in bulk lung tissue from G3 Terc-/- mice compared with wild-type controls. This substantially weakens confidence that the model exhibits robust fibrotic remodeling at the molecular level.
The hydroxyproline data are also not fully convincing. The authors acknowledge technical limitations related to tissue sampling and note that only one treatment condition achieved statistical significance. Given the central therapeutic claims of the manuscript, stronger and more rigorous whole-lung biochemical validation of fibrosis would be expected.
I remain concerned that emphysema-like changes may confound the interpretation of the histologic and architectural findings. Although the terminology was modified from "emphysema" to "emphysema-like features," the phenotype was not rigorously quantified using standard morphometric approaches. Airspace enlargement and altered alveolar architecture could influence interpretation of fibrosis density and apparent structural improvement following treatment.
Finally, I continue to believe that validation in a more robust fibrotic/injury-driven model would substantially strengthen the manuscript. The absence of combining bleomycin with the injury/telomere dysfunction model limits confidence in the translational significance of the therapeutic conclusions, particularly given the relatively mild fibrotic phenotype observed here.

Overall, although the revised manuscript includes substantial additional experiments and analyses, I do not believe the data sufficiently support the strong therapeutic conclusions being made. In my opinion, the concerns regarding fibrosis severity, orthogonal validation, and potential confounding by emphysema-like remodeling remain unresolved.

For these reasons, I would not support publication of the manuscript in its current form.

Referee #2 (Comments on Novelty/Model System for Author):

The results describe a new context for tDDR activation in a mouse model of pulmonary fibrosis. They demonstrate a new application of tASOs. In this sense, novelty is not the strong point of this manuscript. Nevertheless, this manuscript describes a new application of tASOs for pulmonary fibrosis

Referee #2 (Remarks for Author):

The authors have responded to my comments satisfactorily. The revised version, as a whole, is much improved.

Referee #3 (Remarks for Author):

The authors have satisfactorily addressed my major critiques, and the overall quality of the manuscript has substantially improved. I congratulate the authors on their efforts and have no further comments.

As a service to authors, EMBO provides authors with the possibility to transfer a manuscript that one journal cannot offer to publish to another EMBO publication. The full manuscript and if applicable, reviewers reports are automatically sent to the receiving journal to allow for fast handling and a prompt decision on your manuscript. For more details of this service, and to transfer your manuscript to another EMBO title please click on Link Not Available

Please do not share this URL as it will give anyone who clicks it access to your account.

***** Reviewer's comments *****

Referee #1 (Remarks for Author):

After reviewing the revised manuscript and the authors' responses, I appreciate the substantial additional work performed by the authors to address the reviewers' comments. The added analyses, including Ashcroft scoring, additional immunostaining, transcriptomic analyses, and mechanistic studies, clearly strengthened the manuscript compared with the original submission.

However, despite these efforts, I continue to have substantial concerns regarding the strength of the fibrosis phenotype, the rigor of the fibrosis characterization, and the interpretation of the therapeutic effects reported.
While the study addresses an important topic and the mechanistic concept of targeting telomeric DDR is interesting, I remain unconvinced that the data sufficiently support the central conclusion that tASOs meaningfully reverse pulmonary fibrosis in this model.
We respectfully disagree and would like to emphasize that the conclusion is supported by a consistent and robust rescue across multiple independent and complementary disease-associated readouts. In particular, **tASOs treatment promotes**:

- Reduction in total lung DNA damage response (DDR) markers, alveolar epithelial type II cells-specifc DDR and telomere-specifc DDR
- Reduction in cellular senescence, as assessed by senescence-associated transcriptional signatures and by p21 immunostaining
- Reduction in inflammation, including of inflammatory pathways and immune cell infiltration ($CD45^+$ cells)
- **Fibrotic remodeling, demonstrated by** multiple orthogonal approaches, including **histological analyses (Ashcroft score, pathological scoring, Masson's trichrome staining), immunohistochemistry (COL1A1, pSMAD2/3, αSMA), transcriptomic profiling (TGFβ signaling)**

Across all these readouts, tASOs treatment leads to a consistent attenuation of disease-associated phenotypes.

Our conclusions are supported by a coherent and consistent phenotypic rescue across multiple independent analyses. This is **reinforced by integrative approaches such as principal component analysis (PCA),** which further demonstrate the **robust changes induced by short telomeres and** their quantitative **rescue by tASO treatments**.

My major concerns are as follows:

The fibrotic phenotype in the Terc-/- model appears relatively modest and relies heavily on histological interpretation and image-based quantification. Although the authors added Ashcroft scoring and additional immunostaining analyses, the overall fibrosis burden remains difficult to appreciate convincingly at the whole-lung level.

We respectfully disagree with the interpretation that the phenotype is modest. When evaluated using the **Ashcroft score** – the most widely accepted histopathological metrics for pulmonary fibrosis and specifically requested by this reviewer – the **values were** absolutely **comparable to** those observed in the popular model of **bleomycin-induced lung fibrosis** (see Gilhodes et al, PLOS one 2017; Murgo et al, Front. Pharmacol 2023; Kadam and Schnitzer, Int J Mol Sci 2024) (Fig. 3a and see the comparison below). Notably, the Ashcroft score of our mice is in a **similar** range **to** that observed **in IPF patients** (Testa et al, Front Med 2021, see below).
Thus, our genetic model of lung fibrosis has the same degree of fibrosis severity of the bleomycin model and of IPF patients.

Figure for reviewers removed

In regard to image-based approaches, we intentionally employed them to achieve single-cell resolution to accurately capture pathological changes. To strengthen this, **we complemented histological analyses with transcriptional analyses (RNAseq) and biochemical ones (hydroxyproline).**
All these independent analyses revealed **consistent fibrotic alterations**: **any doubts** on the phenotype of the model we employed are therefore **unjustified**.

Importantly, the authors report no significant increase in several canonical fibrosis-associated transcripts (Col1a1, Col1a2, Acta2/α-SMA, and Fn1) in bulk lung tissue from G3 Terc-/- mice compared with wild-type controls. This substantially weakens confidence that the model exhibits robust fibrotic remodeling at the molecular level.
In our model **we did report increased COL1A1 and αSMA at the protein level**, which is what counts and we consider a more direct and reliable indicator of fibrotic remodeling than bulk transcript measurements
From the comments of our chartered pathologist that evaluated our mosue samples: "in the Terc−/− model, the lung phenotype is characterized by parenchymal alterations that vary in extent and severity across animals, but nevertheless consistently define a tissue state marked by an increased inflammatory set point and fibrotic remodeling. Importantly, this phenotype emerges in the absence of exogenous perturbations to lung homeostasis, such as bleomycin, LPS, or other inflammatory challenges. Thus, the histopathological features we describe reflect the intrinsic consequences of telomerase deficiency itself."
As already emphasized, transcriptional changes in structural components of the tissue, including mesenchymal cells and fibroblasts (e.g., upregulation of collagen transcripts), are readily captured in models of acute stromal remodeling. However, **in the more physiological context that we investigated**, the final histomorphological phenotype represents the **cumulative outcome of temporally distributed events**.

Transcriptional upregulation of classical fibrotic genes is typical of acute injury models and is not consistently observed in chronic settings or in human IPF at the whole-tissue level, as we mentioned already in our rebuttal. In contrast, our model reflects a chronic, telomere-driven and age-associated condition, which more closely parallels the human disease context.
In this context, histological collagen accumulation – such as that assessed by Masson's trichrome staining – reflects integrated, long-term extracellular matrix (ECM) deposition and remodeling, which may not be fully captured by snapshot measurements of profibrotic gene expression in bulk mRNA analyses.
Consistently, **transcriptional upregulation of canonical fibrotic markers such as COL1A1, COL1A2, and ACTA2 has not been uniformly reported in bulk lung tissue from IPF patients**. When detected, these changes are often restricted to specific cellular compartments, such as bronchoalveolar lavage (BAL) cells (Tsitoura et al., 2021), or to defined cell populations identified by single-cell RNA sequencing (Tsukui et al., 2020), rather than being evident at the whole-tissue level.
To further assess this point, **we examined the human RNA-seq datasets included in our manuscript (Fig. 9), comparing IPF and control lung tissue. We observed only modest differences in the expression of COL1A1, COL1A2, ACTA2, and FN1, with a statistically significant increase detected only for ACTA2 in a single dataset, which was not reproduced in an independent cohort.**
Moreover, these findings should be interpreted in the context of a broader, multi-layered characterization of the phenotype, including histological, immunohistochemical, and transcriptomic analyses, all of which independently and consistently support the presence of a fibrosis-like condition that worsens with age and is attenuated upon tASOs treatment.

The hydroxyproline data are also not fully convincing. The authors acknowledge technical limitations related to tissue sampling and note that only one treatment condition achieved statistical significance. Given the central therapeutic claims of the manuscript, stronger and more rigorous whole-lung biochemical validation of fibrosis would be expected.
Again, we respectfully disagree. **Hydroxyproline levels are significantly increased in G3 Terc−/− mice compared with wild-type controls**. This provides independent biochemical validation of enhanced collagen deposition and independently demonstrate a fibrotic phenotype in this model.

Text removed because it referred to a figure intended for reviewers only and to unpublished findings.

Figure for reviewers removed

This results aligns and complement the additional measurements described in our manuscript, including histological (Ashcroft score, pathological scoring, Masson's trichrome staining), immunohistochemical (COL1A1, pSMAD2/3, αSMA), and transcriptomic analyses (TGFβ signaling), all of which independently and consistently support both the presence of a fibrosis-like phenotype and its attenuation upon tASOs treatment.

I remain concerned that emphysema-like changes may confound the interpretation of the histologic and architectural findings. Although the terminology was modified from "emphysema" to "emphysema-like features," the phenotype was not rigorously quantified using standard morphometric approaches. Airspace enlargement and altered alveolar architecture could influence interpretation of fibrosis density and apparent structural improvement following treatment.

We respectfully disagree that this represents a confounding factor and we believe that there may be a **misunderstanding of our quantification strategy**. In fact our quantification strategy was **specifically designed to avoid bias introduced by airspace enlargement and changes in alveolar architecture**. **Rather than relying on field-based measurements** (as often applied in this kind of studies), which are indeed sensitive to airspace expansion, **we instead intentionally quantify signal as density (i.e., signal normalized to tissue area), making the analysis intrinsically independent of airspace expansion and changes in alveolar architecture**.

This approach ensures that readouts such as marker expression and cell-associated **signals are normalized independently of tissue density or airspace size**, thereby minimizing the impact of structural alterations on quantitative outcomes. In this context, airspace enlargement does not artificially inflate or reduce the measured signal, as quantifications are not dependent on the total tissue area analyzed.

Therefore, we do not believe that emphysema-like features confound the interpretation of fibrosis-related measurements in our study.

Nevertheless, as this aspect is not central to the manuscript, **we are willing to remove or further de-emphasize this analysis** to avoid potential ambiguity.

Finally, I continue to believe that validation in a more robust fibrotic/injury-driven model would substantially strengthen the manuscript. The absence of combining bleomycin with the injury/telomere dysfunction model limits confidence in the translational significance of the therapeutic conclusions, particularly given the relatively mild fibrotic phenotype observed here.

We have not included validation in a bleomycin or combined injury model for the reasons already detailed above, including the distinct biological nature of acute injury models compared to chronic, telomere-driven pathology.

Importantly, **this point was clearly and explicitly discussed during the editorial process, and we agreed with our Editor** that inclusion of an additional model was not required.
We note that we have obtained preliminary evidence indicating that tASOs may also be effective in an independent fibrosis model, further supporting the generalizability of our findings.
Overall, although the revised manuscript includes substantial additional experiments and analyses, I do not believe the data sufficiently support the strong therapeutic conclusions being made. In my opinion, the concerns regarding fibrosis severity, orthogonal validation, and potential confounding by emphysema-like remodeling remain unresolved.

We respectfully disagree and summarize below why these concerns are fully addressed by the data presented:

• Fibrosis severity:
The extent of fibrosis in the G3 Terc−/− model, as assessed by Ashcroft scoring, is similar to that reported in belomycin-induced lung fibrosis and IPF patients. Importantly, fibrosis is supported by multiple independent readouts, including histological analyses, increased collagen deposition, and elevated COL1A1 and αSMA at the protein level, as well as a significant increase in hydroxyproline compared to wild-type controls.

• Orthogonal validation:
The phenotype has been extensively characterized using complementary approaches, including histology (Ashcroft score, Masson's trichrome), immunohistochemistry (COL1A1, pSMAD2/3, αSMA, CD45), and transcriptomics (senescence, inflammatory and TGFβ pathways). These alterations show a clear age-dependent progression and are consistently attenuated upon tASOs treatment. In addition, integrative analyses (e.g., PCA) support a coherent and global therapeutic effect across independent readouts.

• Potential confounding by emphysema-like remodeling:
This is mitigated by the quantification strategy, which was intentionally performed at the single-cell level rather than using area- or field-based measurements. This approach minimizes bias introduced by airspace enlargement or changes in tissue architecture, ensuring that fibrotic readouts are independent of alveolar structural alterations.

Taken together, the data provide consistent, convergent and multi-layered evidence supporting both the presence of a fibrosis-like phenotype in this model and its attenuation upon tASOs treatment.

For these reasons, I would not support publication of the manuscript in its current form.

Referee #2 (Comments on Novelty/Model System for Author):

The results describe a new context for tDDR activation in a mouse model of pulmonary fibrosis. They demonstrate a new application of tASOs. In this sense, novelty is not the strong point of this manuscript. Nevertheless, this manuscript describes a new application of tASOs for pulmonary fibrosis

Referee #2 (Remarks for Author):

The authors have responded to my comments satisfactorily. The revised version, as a whole, is much improved.

Referee #3 (Remarks for Author):

The authors have satisfactorily addressed my major critiques, and the overall quality of the manuscript has substantially improved. I congratulate the authors on their efforts and have no further comments.

3rd Jul 2026

Dear Dr. d'Adda di Fagagna,

Thank you for your message requesting that we reconsider our decision on your manuscript. Following peer review and revision, your manuscript was rejected in light of Referee #1's continued concerns regarding the robustness of the fibrotic phenotype. In your appeal, you provided additional arguments in support of the suitability of your model and the conclusions of the study. As indicated in our previous correspondence, we sought independent advice on the manuscript and the referee reports from an expert in the field.

We have now received this assessment, and the advisor is generally supportive of publication of your work. In particular, they conclude that:

"Overall, this is a solid piece of work with multiple validations, and the conclusions of the authors are supported by the experimental data. The experiments seem well designed and conducted, with appropriate power sizes for the experimental groups and statistical analyses."

Regarding the concerns about the fibrosis model, the advisor acknowledges its limitations but notes that these are shared by other established models of pulmonary fibrosis:
"Obviously, there is not a perfect murine model for human IPF; all existing models have pros and cons. However, genetic Terc-/- mice represent a consolidated mouse model that captures the telomere biology of IPF patients, epithelial dysfunction, and some aspects of age-related senescence and lung regenerative failure. It is true that the fibrotic phenotype, as reported by Reviewer 1, can develop slowly or incompletely in this model (typically G3 but sometimes G4 or later). However, bleomycin-induced pulmonary fibrosis models have other limitations too, including the partial resolution of fibrosis and the fact that fibrosis is initiated by an acute insult rather than sustained epithelial dysfunction."

Based on the overall strength of the study, the advisor recommends publication following minor revision, while suggesting that several conclusions be expressed more cautiously and that the limitations of the study be acknowledged more explicitly. Specifically, the advisor recommends moderating statements that imply definitive demonstration of fibrosis reduction (for example, using "as shown" or "as supported by" rather than "as demonstrated") and expanding the Discussion to acknowledge that the validation of fibrosis and the therapeutic effects of tASOs does not include longitudinal imaging (e.g. microCT) or lung function assessments (e.g. FlexiVent forced oscillation technique or plethysmography).

Having carefully considered the appeal together with this independent assessment, we would therefore like to invite you to submit a further revised version of the manuscript addressing the advisor's comments.

Additionally, please address the following editorial matters:
- Please remove the yellow highlights and indicate any new modification in track changes mode.
- Alessia di Lillo is affiliated Thermo Fisher Scientific, this should be mentioned in the Disclosure and Competing Interests
- Funding: Please enter the complete list of funders into the line-by-line list, including the funders listed in the Comments section. The green tick mark does not need to appear.
- Correct the order and headings of the sections in the manuscript text to: Abstract / The Paper Explained / Introduction / Results / Discussion / Methods / Data Availability / Acknowledgements / Disclosure and Competing Interests Statement / References / Figure Legends / Expanded View Figure Legends
- In the methods:
1. Animal experiments: please provide permit number, and indicate the housing and husbandry conditions, and age of the mice at the time of experiments.
2. Statistics: please provide a statement on randomization, sample size, and inclusion/exclusion criteria.
- BIORENDER: remove from Ackn and add to the Methods section, in a paragraph using this format:
Graphics:
(some of the... OR Figure #... OR synopsis) Graphics were created with BioRender.com.
- The section "Declaration of Generative AI and AI-assisted technologies in the writing process" should be listed in the methods, after the Graphics section.
- Data Availability: please add a URL for the E-MTAB-14908 dataset and note that this and the zenodo link must be publicly accessible before acceptance of the manuscript. Remove the following text: "The study includes three biological replicates for old wild-type samples, five replicates for adult wild-type samples, and five replicates each for control G3 Terc-/-, anti-teloG Terc-/-, and anti-teloC G3 Terc-/- conditions. The experimental design is detailed in the Methods section."
- References: to directly cite datasets that were re-used and obtained from public databases, please use the following format:
In the main text, data citations are formatted as follows: "Data ref: Smith et al, 2001" or "Data ref: NCBI Sequence Read Archive PRJNA342805, 2017". In the Reference list, data citations must be labeled with "[DATASET]". A data reference must provide the database name, accession number/identifiers and a resolvable link to the landing page from which the data can be accessed at the end of the reference.

- There are four files uploaded as datasets. The labelling should be checked so that it is congruent with the legends. Are Datasets EV1 and EV2 (uploaded as Dataset EV3 and EV4) source data?
- The visual abstract should be resized to 550 px x 300 px
- The individual panels of figures EV1-EV9 should be referenced in the text.
- Please address the queries from our data editors in the figure legends:
1. Please note that the exact p values are not provided in the legends of figures 1A,C; 3B,E; 5G; 8D; EV-1E; EV-2C.
2. Please indicate the statistical test used for data analysis in the legends of figures EV-4A-C; EV-6D,E.
3. Please note that the scale bar is missing for figures EV-1D,G; EV-3B,E.

I look forward to seeing a revised form of your manuscript as soon as possible.

With kind regards,

Lise Roth

Lise Roth, Ph.D
Senior Editor
EMBO Molecular Medicine

The authors addressed the remaining editorial issues.

23rd Jul 2026

Dear Dr. d'Adda di Fagagna, Dear Fabrizio.

Thank you for submitting your revised files. I am pleased to inform you that your manuscript is accepted for publication and is now being sent to our publisher to be included in the next available issue of EMBO Molecular Medicine.

Your manuscript will be processed for publication by EMBO Press. It will be copy edited and you will receive page proofs prior to publication. Please note that you will be contacted by Springer Nature Author Services to complete licensing and payment information.

You may qualify for financial assistance for your publication charges - either via a Springer Nature fully open access agreement or an EMBO initiative. Check your eligibility: https://link.springer.com/journal/44321/how-to-publish-with-us

Should you be planning a Press Release on your article, please notify the production team via this form in order to coordinate publication and release dates: https://forms.cloud.microsoft/e/eH4A8JVP6g

If you have any questions, please do not hesitate to contact the Editorial Office. Thank you for your contribution to EMBO Molecular Medicine.

With kind regards,

Lise

Lise Roth, Ph.D
Senior Editor
EMBO Molecular Medicine

>>> Please note that it is EMBO Molecular Medicine policy for the transcript of the editorial process (containing referee reports and your response letter) to be published as an online supplement to each paper. If you do NOT want this, you will need to inform the Editorial Office via email immediately. More information is available here: https://link.springer.com/partners/embo-press/editorial-policies#Peer%20review

EMBO Press Author Checklist

Corresponding Author Name: Fabrizio d'Adda di Fagagna	
Journal Submitted to: EMBO Molecular Medicine	
Manuscript Number: EMM-2025-21982-V3-Q	

USEFUL LINKS FOR COMPLETING THIS FORM
The EMBO Journal - Author Guidelines
EMBO Reports - Author Guidelines
Molecular Systems Biology - Author Guidelines
EMBO Molecular Medicine - Author Guidelines

Reporting Checklist for Life Science Articles (updated January
This checklist is adapted from Materials Design Analysis Reporting (MDAR) Checklist for Authors. MDAR establishes a minimum set of requirements in transparent reporting in the life sciences (see Statement of Task: 10.31222/osf.io/9sm4x). Please follow the journal's guidelines in preparing your
Please note that a copy of this checklist will be published alongside your article.

Abridged guidelines for figures
1. Data
The data shown in figures should satisfy the following conditions:
- the data were obtained and processed according to the field's best practice and are presented to reflect the results of the experiments in an accurate and unbiased manner.
- ideally, figure panels should include only measurements that are directly comparable to each other and obtained with the same assay.
- plots include clearly labeled error bars for independent experiments and sample sizes. Unless justified, error bars should not be shown for technical
- if n<5, the individual data points from each experiment should be plotted. Any statistical test employed should be justified.
- Source Data should be included to report the data underlying figures according to the guidelines set out in the authorship guidelines on Data

2. Captions
Each figure caption should contain the following information, for each panel where they are relevant:
- a specification of the experimental system investigated (eg cell line, species name).
- the assay(s) and method(s) used to carry out the reported observations and measurements.
- an explicit mention of the biological and chemical entity(ies) that are being measured.
- an explicit mention of the biological and chemical entity(ies) that are altered/varied/perturbed in a controlled manner.
- the exact sample size (n) for each experimental group/condition, given as a number, not a range;
- a description of the sample collection allowing the reader to understand whether the samples represent technical or biological replicates (including how many animals, litters, cultures, etc.).
- a statement of how many times the experiment shown was independently replicated in the laboratory.
- definitions of statistical methods and measures:
  - common tests, such as t-test (please specify whether paired vs. unpaired), simple χ2 tests, Wilcoxon and Mann-Whitney tests, can be unambiguously identified by name only, but more complex techniques should be described in the methods section;
  - are tests one-sided or two-sided?
  - are there adjustments for multiple comparisons?
  - exact statistical test results, e.g., P values = x but not P values < x;
  - definition of 'center values' as median or average;
  - definition of error bars as s.d. or s.e.m.

Please complete ALL of the questions below.
Select "Not Applicable" only when the requested information is not relevant for your study.

Materials

Newly Created Materials	Information included in the manuscript?	In which section is the information available? (Reagents and Tools Table, Materials and Methods, Figures, Data Availability Section)
New materials and reagents need to be available; do any restrictions apply?	Not Applicable	

Antibodies	Information included in the manuscript?	In which section is the information available? (Reagents and Tools Table, Materials and Methods, Figures, Data Availability Section)
For **antibodies** provide the following information:<br>- Commercial antibodies: RRID (if possible) or supplier name, catalogue number and or/clone number<br>- Non-commercial: RRID or citation	Yes	Reagent table

DNA and RNA sequences	Information included in the manuscript?	In which section is the information available? (Reagents and Tools Table, Materials and Methods, Figures, Data Availability Section)
**Short novel DNA or RNA including primers, probes**: provide the sequences.	Yes	Reagent table

Cell materials	Information included in the manuscript?	In which section is the information available? (Reagents and Tools Table, Materials and Methods, Figures, Data Availability Section)
**Cell lines:** Provide species information, strain. Provide accession number in repository **OR** supplier name, catalog number, clone number, and/**OR** RRID.	Not Applicable	
**Primary cultures:** Provide species, strain, sex of origin, genetic modification status.	Not Applicable	
Report if the cell lines were recently **authenticated** (e.g., by STR profiling) and tested for mycoplasma contamination.	Not Applicable	

Experimental animals	Information included in the manuscript?	In which section is the information available? (Reagents and Tools Table, Materials and Methods, Figures, Data Availability Section)
**Laboratory animals or Model organisms:** Provide species, strain, sex, age, genetic modification status. Provide accession number in repository **OR** supplier name, catalog number, clone number, **OR** RRID.	Yes	Materials and Methods
**Animal observed in or captured from the field:** Provide species, sex, and age where possible.	Not Applicable	
Please detail **housing and husbandry conditions**.	Yes	Materials and Methods

Plants and microbes	Information included in the manuscript?	In which section is the information available? (Reagents and Tools Table, Materials and Methods, Figures, Data Availability Section)
**Plants:** provide species and strain, ecotype and cultivar where relevant, unique accession number if available, and source (including location for collected wild specimens).	Not Applicable	
**Microbes:** provide species and strain, unique accession number if available, and source.	Not Applicable	

Human research participants	Information included in the manuscript?	In which section is the information available? (Reagents and Tools Table, Materials and Methods, Figures, Data Availability Section)
If collected and within the bounds of privacy constraints report on age, sex and gender or ethnicity for all study participants.	Not Applicable	

Core facilities	Information included in the manuscript?	In which section is the information available? (Reagents and Tools Table, Materials and Methods, Figures, Data Availability Section)
If your work benefited from core facilities, was their service mentioned in the acknowledgments section?	Yes	Acknowledgments

Design

Study protocol	Information included in the manuscript?	In which section is the information available? (Reagents and Tools Table, Materials and Methods, Figures, Data Availability Section)
If study protocol has been **pre-registered, provide DOI in the manuscript**. For clinical trials, provide the trial registration number **OR** cite DOI.	Not Applicable	
Report the **clinical trial registration number** (at ClinicalTrials.gov or equivalent), where applicable.	Not Applicable	

Laboratory protocol	Information included in the manuscript?	In which section is the information available? (Reagents and Tools Table, Materials and Methods, Figures, Data Availability Section)
Provide DOI OR other citation details if **external detailed step-by-step protocols** are available.	Yes	Materials and Methods

Experimental study design and statistics	Information included in the manuscript?	In which section is the information available? (Reagents and Tools Table, Materials and Methods, Figures, Data Availability Section)
Include a statement about **sample size** estimate even if no statistical methods were used.	Yes	Materials and Methods
Were any steps taken to minimize the effects of subjective bias when allocating animals/samples to treatment (e.g. **randomization procedure**)? If yes, have they been described?	Yes	Materials and Methods
Include a statement about **blinding** even if no blinding was done.	Yes	Materials and Methods
Describe **inclusion/exclusion criteria** if samples or animals were excluded from the analysis. Were the criteria pre-established?<br><br>If sample or data points were omitted from analysis, report if this was due to attrition or intentional exclusion and provide justification.	Yes	Materials and Methods
For every figure, are **statistical tests** justified as appropriate? Do the data meet the assumptions of the tests (e.g., normal distribution)? Describe any methods used to assess it. Is there an estimate of variation within each group of data? Is the variance similar between the groups that are being statistically compared?	Yes	Materials and Methods

Sample definition and in-laboratory replication	Information included in the manuscript?	In which section is the information available? (Reagents and Tools Table, Materials and Methods, Figures, Data Availability Section)
In the figure legends: state number of times the experiment was **replicated** in laboratory.	Yes	Materials and Methods
In the figure legends: define whether data describe **technical or biological replicates**.	Yes	Materials and Methods

Ethics

Ethics	Information included in the manuscript?	In which section is the information available? (Reagents and Tools Table, Materials and Methods, Figures, Data Availability Section)
Studies involving **human participants**: State details of **authority granting ethics approval** (IRB or equivalent committee(s), provide reference number for approval.	Not Applicable	
Studies involving **human participants**: Include a statement confirming that **informed consent** was obtained from all subjects and that the experiments conformed to the principles set out in the WMA Declaration of Helsinki and the Department of Health and Human Services Belmont Report.	Not Applicable	
Studies involving **human participants: For** publication of **patient photos**, include a statement confirming that consent to publish was obtained.	Not Applicable	
Studies involving experimental **animals**: State details of **authority granting ethics approval** (IRB or equivalent committee(s), provide reference number for approval. Include a statement of compliance with ethical regulations.	Yes	Materials and Methods
Studies involving **specimen and field samples:** State if relevant **permits** obtained, provide details of authority approving study; if none were required, explain why.	Not Applicable	

Dual Use Research of Concern (DURC)	Information included in the manuscript?	In which section is the information available? (Reagents and Tools Table, Materials and Methods, Figures, Data Availability Section)
Could your study fall under dual use research restrictions? Please check biosecurity documents and list of **select agents and toxins** (CDC): https://www.selectagents.gov/sat/list.htm	Not Applicable	
If you used a select agent, is the security level of the lab appropriate and reported in the manuscript?	Not Applicable	
If a study is subject to dual use research of concern regulations, is the name of the **authority granting approval and reference number** for the regulatory approval provided in the manuscript?	Not Applicable	

Reporting

The MDAR framework recommends adoption of discipline-specific guidelines, established and endorsed through community initiatives. Journals have their own policy about requiring specific guidelines and recommendations to complement MDAR.

Adherence to community standards	Information included in the manuscript?	In which section is the information available? (Reagents and Tools Table, Materials and Methods, Figures, Data Availability Section)
State if relevant guidelines or checklists (e.g., **ICMJE, MIBBI, ARRIVE, PRISMA**) have been followed or provided.	Not Applicable	
For **tumor marker prognostic studies,** we recommend that you follow the **REMARK** reporting guidelines (see link list at top right). See author guidelines, under 'Reporting Guidelines'. Please confirm you have followed these guidelines.	Not Applicable	
For **phase II and III randomized controlled trials**, please refer to the **CONSORT** flow diagram (see link list at top right) and submit the CONSORT checklist (see link list at top right) with your submission. See author guidelines, under 'Reporting Guidelines'. Please confirm you have submitted this list.	Not Applicable	

Data Availability

Data availability	Information included in the manuscript?	In which section is the information available? (Reagents and Tools Table, Materials and Methods, Figures, Data Availability Section)
Have **primary datasets** been deposited according to the journal's guidelines (see 'Data Deposition' section) and the respective accession numbers provided in the Data Availability Section?	Yes	Data availability
Were **human clinical and genomic datasets** deposited in a public access-controlled repository in accordance to ethical obligations to the patients and to the applicable consent agreement?	Not Applicable	
Are **computational models** that are central and integral to a study available without restrictions in a machine-readable form? Were the relevant accession numbers or links provided?	Yes	Data availability
If publicly available data were reused, provide the respective **data citations in the reference list**.	Yes	References
